# Molecular Mechanism of Response and Adaptation of Antioxidant Enzyme System to Salt Stress in Leaves of *Gymnocarpos przewalskii*

**DOI:** 10.3390/plants12193370

**Published:** 2023-09-25

**Authors:** Jianwei Qi, Yongzhong Luo, Haixia Huang, Songsong Lu, Fei Zhao, Zhuo Deng, Yingde Qiu

**Affiliations:** College of Forestry, Gansu Agricultural University, Lanzhou 730070, China; xiaoqi_325_qi@163.com (J.Q.); hhx@gsau.edu.cn (H.H.); luss86_gsau@163.com (S.L.); 15085029254@163.com (F.Z.); dzhuo9486@163.com (Z.D.); 18294650813@163.com (Y.Q.)

**Keywords:** *Gymnocarpos przewalskii*, antioxidant enzyme system, gene expression, molecular evolution, protein structure

## Abstract

The antioxidant enzyme system is the main defense system responsible for maintaining cellular reactive oxygen species (ROS) homeostasis and normal plant growth and development after saline stress. In this study, we identified and characterized the members of the *SOD*, *APX* and *CAT* gene families of the antioxidant enzyme system in *Gymnocarpos przewalskii,* using plant physiology and molecular biology methods, and analyzed the pattern of enzyme activity in response to NaCl stress. It was found that seven, six and two genes of *SOD*, *APX* and *CAT* gene families, respectively, were expressed in the leaf tissue of *G. przewalskii*, in which most of the genes were significantly upregulated under NaCl stress, and the enzymatic activities were in accordance with the gene expression. Three positive selection sites in the *GpCAT1* gene can increase the hydrophilicity of the GpCAT1 protein, increase the volume of the active site and increase the affinity for H_2_O_2_, thus improving the catalytic efficiency of GpCAT1. The results of the present study provide new insights for further investigations of the evolution and function of the *SOD*, *APX* and *CAT* gene families in *G. przewalskii* and their essential roles under salt stress, and the findings will be useful for revealing the molecular mechanism of salt tolerance and breeding of salt-tolerant plants.

## 1. Introduction

With the acceleration of global industrial modernization, soil salinization has become more and more serious, and salinity stress has become the most important abiotic stress limiting the growth and development of plants, which seriously restricts the development of agricultural productivity [1]. Biological amendment technology is one of the most important ways to improve saline soils, and the selection and cultivation of salt-tolerant plants are a prerequisite for its success [2]. Thus, the investigation of the physiological, biochemical and molecular response mechanisms of salt-tolerant plants to salt stress and the breeding of salt-tolerant crop varieties are currently of great importance. In recent years, with the continuous improvement and development of high-throughput sequencing technology, revealing the molecular mechanisms of salt-tolerant plants adapting to salt stress has become a hotspot of current research, important findings have been reported in hormone signaling [3], oxidative stress [4], starch-sucrose metabolism [5], etc.

Reactive oxygen species (ROS) are the inevitable products of aerobic metabolism, and under normal physiological conditions, as signaling molecules, they can mediate plants’ stress response to various external stimuli. However, when plants experience both biotic and abiotic stress, organelles such as mitochondria, chloroplasts and peroxisomes, all generate high amounts of ROS, leading to the overaccumulation of intracellular ROS which results in oxidative stress in the plant [6]. To prevent the damage of ROS during aerobic conditions, organisms have formed a variety of defense mechanisms during evolution, including evasion defense, antioxidant defense and post-oxidative defense, and antioxidant defense is the main mechanism for plant organisms to defend against oxidative stress. Antioxidant system is the main pathway of antioxidant defense, including antioxidant enzyme system and antioxidants [7]. Antioxidant enzyme system mainly includes superoxide dismutase (SOD), catalase (CAT) and ascorbate peroxidase (APX). The antioxidative enzyme system is an important pathway for scavenging ROS in plants, which protects plants from ROS during plant growth and development [8]. There is increasing evidence that antioxidant enzymes play a crucial role in maintaining ROS homeostasis in plant cells under salt stress [6,8,9].

SOD is a metalloenzyme that catalyzes the disproportionation reaction of O_2_^-^ and is at the core of the antioxidant enzyme system. A variety of SOD isoenzymes exist in plants, which are classified into three subfamilies, Cu/Zn-SOD (CSD), Mn-SOD (MSD) and Fe-SOD (FSD), based on the metal ions in their active centers [10]. Enhanced SOD activity in plants significantly increases plant resistance to a wide range of adversity stresses. Ma found that SOD activity was significantly elevated in the tolerant lines under salinity stress compared with the sensitive lines of rice, which significantly improved their tolerance to salt stress [11]. APX belongs to the family of peroxidases that catalyze H_2_O_2_-dependent oxidation by the ascorbate-glutathione pathway, which is widely present in the plant kingdom, and plays a crucial role in the regulation of cellular H_2_O_2_ levels [12,13]. Regulation of the *APX* genes expression at the transcriptome level can alter plant tolerance to salt stress. For example, the overexpression of the *Arabidopsis thaliana’s APX1* gene increased salt tolerance in *Brassica juncea* [14]. However, not all *APX* genes can be induced to express under salt stress, for example, pea chloroplast’s *sAPX* expression was upregulated, and *tAPX* gradually decreased under salt stress, but the expression increased in salt-tolerance in pea [15]. CAT is one of the antioxidant enzymes widely found in plants and animals; it is a tetrameric heme enzyme consisting of four identical peptide chain subunits with a conserved active center and ferrous heme-binding site, which is mainly found in peroxisomes, glyoxylate cyclosomes and cytoplasm of the cells, and partially distributed in mitochondria and chloroplasts [16]. Numerous studies have shown that the overexpression of the CAT gene increases plant tolerance to salt stress. The role of externally applied H_2_O_2_ to reverse the growth inhibition of maize seedlings by salt stress was mainly attributed to the increase in CAT activity [17]. Salt-tolerant tomato roots showed low levels of H_2_O_2_, lipid peroxidation and high activities of antioxidant enzymes such as CAT, SOD and APX in mitochondria and peroxisomes [18].

*Gymnocarpos przewalskii* belongs to the genus *Gymnocarpos* of the family Caryophyllaceae and is one of the most important species of rocky desert vegetation with high tolerance to abiotic stresses such as drought, soil infertility, salinity and wind erosion and sand burial, plays a positive role in the prevention of desertification and the maintenance of the balance of desert ecosystems [19,20,21]. *G. przewalskii* belongs to an ancient group of saline plants and is mainly distributed in the desert regions of northwestern China, where precipitation is scarce and soil salinity is high [19]. After a long period of natural selection, *G. przewalskii* shows typical characteristics of adaptation to extreme environments, such as salinity and drought, in terms of physiology, biochemistry and morpho-anatomical structure [19,21,22]. For example, the leaves of *G. przewalskii* are fleshy, subsessile and linear, with typical leaf morphology of saline plants [22]. At present, studies on the adaptation of *G. przewalskii* to salt stress have been reported, but most of them only dealt with the effects of salt stress on seed germination [23] and photosynthetic properties [24], but the response of seed-associated antioxidant enzyme activities to salt stress and its molecular bases have not been investigated. Therefore, the present study was conducted to simulate the salt environment by treating the seedling substrate with different concentrations of NaCl and to investigate the antioxidant enzyme system of *G. przewalskii* in terms of enzyme activity, gene family composition, phylogenetic relationship, gene function, expression at the transcriptome level and protein structure by combining the research methods from the disciplines of plant physiology, genomics, transcriptomics, molecular evolution and computational biology. It provides basic data for studying the molecular mechanism of salt tolerance of *G. przewalskii*.

## 2. Materials and Methods

### 2.1. Experimental Materials

Seeds of *G. przewalskii* were harvested in 2022 from the Botanical Garden of Anxi Extremely Arid Desert National Nature Reserve, located in Guazhou County, Jiuquan City, Gansu Province, China (95°44′12″ E, 40°29′53″ N). The test seeds were sown in substrates (mixture of peat soil and vermiculite in a ratio of 3:1) treated with different concentrations0, 0.5%, 1.5% and 2.5%) and incubated in an artificial climate chamber until the plants were robust (100 d). Then, young, robust, uniformly sized leaves from the same location were collected for transcriptome sequencing and enzyme activity assays. The incubation conditions were 28 °C, 14 h/d of light (h) and 60% humidity (t). The leaves were snap-frozen with liquid nitrogen and stored in an ultra-low temperature refrigerator (−80 °C).

### 2.2. Transcriptome Sequencing and Transcript Assembly

For transcriptome sequencing, 0.6 g (3 biological replicates) leaves of each treatment were collected. Total RNA was extracted and purified with the Invitrogen Trizol reagent (no. 15596-018), and the concentration and integrity of the RNA samples were tested with the Agilent 2100 RNA Nano 6000 test kit (Agilent Technologies, Santa Clara, CA, USA). The samples were sent to Arnold UDA Genentech (Beijing, China) Limited for the transcriptome sequencing. The sequencing platform was Illumina, and the sequencing data size of each sample was 6.0 Gb. Perl scripting was used to filter out the reads with low sequencing quality values in the raw transcriptome sequencing data, under the following conditions: unknown bases (N) exceeding 5% of the total reads and low quality bases (<7) exceeding 65% of the total reads. Transcriptome assembly was performed using Trinity software (2.11.0) with default parameters [25].

For qPCR, total RNA from leaves was extracted using CTAB method and reverse transcribed into cDNA, using a reverse transcription kit (SweScript All-in-One RT SuperMix, G3337, Wuhan Servicebio Technology Co., Ltd., Wuhan, Hubei, China). According to the sequences of the target genes, qRT-PCR primers were designed separately (Table S1). The qRT-PCR was performed according to the reaction system in Table 1, and three technical replicate experiments were carried out. Relative expression analysis was performed using the 2^−∆∆CT^ method.

### 2.3. Enzyme Activity Determination

From each NaCl treatment, 0.2 g of fresh leaves were weighed separately, and the enzyme solution was extracted using phosphate buffer at pH 7.8. SOD [26] activity was determined using the nitrogen blue tetrazolium reduction method, and APX [27] and CAT [28] activities were accomplished by UV spectrophotometry.

### 2.4. Gene Identification

The genomic, proteomic and annotation files of *Chenopodium pallidicaule* (GCA_001687005.1), *Beta vulgaris* (GCF_026745355.1), *Heliosperma pusillum* (GCA_022085475.1), *Silene uniflor* (GCA_018983105.1) and *Spinacia oleracea* (GCF_020520425.1) were downloaded from NCBI database (https://www.ncbi.nlm.nih.gov/ (accessed on 19 July 2022)). Pfam models corresponding to the *SOD*, *APX* and *CAT* gene families were downloaded from the Pfam database (http://pfam.xfam.org/ (accessed on 23 July 2022)), and the Pfam numbers were obtained for SOD (Cu/Zn-SOD: PF00080; Fe-SOD: PF00081; Mn-SOD: PF02777), APX (PF00141) and CAT (PF00199, PF06628).

In this study, the identification of SOD, APX and CAT genes in species of Centrospermae was accomplished in two steps. The first step started with the identification of SOD, APX and CAT genes of *C. pallidicaule*, *B. vulgaris*, *H. pusillum*, *S. uniflora* and *S. oleracea*. Specifically, the protein sequences containing the conserved protein domains of each gene family were obtained by searching the downloaded genome, using the Linux-based program Hmmseach (E-value < 0.01), then the new Pfam models were constructed based on the sequences obtained in the previous step, using the Hmmbuild program, and then the first step was repeated to obtain the candidate members of each gene family. The conserved structural domains were then further analyzed using NCBI-CDD (https://www.ncbi.nlm.nih.gov/cdd/ (accessed on 9 December 2022)) and Pfam (http://www.sanger.ac.uk/software/pfam/ (accessed on 9 December 2022)) to eliminate sequences that did not contain the corresponding conserved structural domains. The sequences obtained were then compared with the genome files, using Blastn to determine whether there were any missing genes (pseudogenes). The second step was to identify SOD, APX and CAT genes expressed in *G. przewalskii* leaf tissues, which was performed by aligning sequences of gene family members identified in the first step to the CDS sequences of SOD, APX and CAT gene family members from *G. przewalskii* leaf tissue transcripts, using native Blastn. When multiple variable shears appeared in the aligned results, the longest CDS sequences were selected based on the homology for the subsequent analyses. The parameters of Blastn (2.12.0) software were set as follows: output format at 7, evalue at 1 × 10^−5^ and other parameters at default. The CDS of the *SOD*, *APX* and *CAT* gene families of *A. thaliana* and *Oryza sativa* were also downloaded. These sequences were used to infer the phylogenetic relationships of the corresponding genes in *G. przewalskii*.

### 2.5. Reconstruction of Phylogenetic Relationships and Analysis of Gene Structure, Conserved Motifs and Conserved Domains of Proteins

Phylogenetic trees for the *SOD*, *APX* and *CAT* gene families of *A. thaliana*, *O. sativa*, *B. vulgaris* and *G. przewalskii* were constructed according to the results of gene identification, using PhyML-3.1 [29] based on the maximum likelihood method. Model prediction was performed using jModelTest (2.1.7) software [30], and the optimal model was calculated according to the Akaike Information Criterion (AICc). The sequence type of PhyML-3.1 was selected as interleaved, the model was selected using the optimal model predicted above, the Subtree pruning and regrafting (SPR) method was selected as the search method for the optimal evolutionary tree, bootstrap was set to 1000 generations, and other parameters were set to default values. Gene structure mapping was performed using the online software GSDS 2.0 [31] (http://gsds.gao-lab.org/ (accessed on 15 March 2023)). Motif prediction was performed using the online website MEME [32] (http://meme-suite.org/tools/meme (accessed on 15 March 2023)), and the length of the motifs was set between 6 and 200. Default parameters were used for the other parameters, and a *p*-value of less than 0.05 was considered a credible result. Domains were predicted using NCBI-CDD. Finally, CFVisual_V (2.1.5) was used to complete the visualization of the above gene information.

### 2.6. Functional Annotation of Genes and Prediction of Protein Interactions

Gene function annotation was performed using the ggNOG-mapper [33] server (http://eggnog-mapper.embl.de/ (accessed on 3 April 2023)) based on the Gene Ontology (GO) terminology of Biological Process, Molecular Function and Cellular Component categories. TBtools [34] was used to organize the annotation results after completion of the annotation. Protein interaction prediction was performed using the STRING [35] server (https://cn.string-db.org/ (accessed on 4 April 2023)), and the *S. oleracea* genome was selected as the background genome with an average confidence of 0.7.

### 2.7. Gene Expression and qRT-PCR Analysis

Gene expression value was calculated using Bowtie2 (2.2.9) [36] to align the CDS of each member of the *SOD*, *APX* and *CAT* gene families of *G. przewalskii* to the leaf transcriptome data (not published), and the number of read segments on the alignment was counted using a custom Perl script. RPKM was expressed as the number of reads per million reads aligned to genes per Kb base. Finally, the significance of differences between treatments (*p* < 0.05) was analyzed by one-way ANOVA, using SPSS (22.0), and plotted using Origin 2021.

### 2.8. Physical and Chemical Properties and Molecular Evolution Analysis of Protein

Protein physicochemical properties of identified protein sequences in the antioxidant enzyme system-related genes of *G. przewalskii* were analyzed using the online tool ExPASy (https://web.expasy.org/protparam/ (accessed on 15 April 2023)).

Selection pressure and positive selection site analyses were performed for each member of *G. przewalskii SOD*, *APX* and *CAT* gene families, using the site model, branch model and branch-site model in PAML4 (v4.9j) [37]. The topological structures of the six species are ((*G. przewalskii* (*S. uniflora*, *H. pusillum*)) and (*B. vulgaris*,(*C. pallidicaule*, *S. oleracea*))). Among the site models, the M0, M3, M1, M2, M7 and M8 models were selected to evaluate the ω values of each site of the CDS. The one-ratio and two-ratio models in the branch model (assuming different ω values for foreground and background branches) were used to calculate the selection pressure for the antioxidant enzyme genes of *G. przewalskii*. Finally, based on the results of the branch model, model A0 and model A1 in the branch-site model (selecting *G. przewalskii* as the foreground branch) were selected to calculate the potential positive selection sites in the antioxidant enzyme genes of *G. przewalskii*, and the probability of positive selection sites was calculated using Bayes Empirical Bayes (BEB). In addition, all three models mentioned above were analyzed for the significance of differences between M3 and M0, M1 and M2, M8 and M7, one-ratio and two-ratio and A0 and A1 models, using likelihood ratio tests (LRTs).

### 2.9. Protein Structural Modeling and Comparison

The homology template search was first performed using SWISS-MODEL [38], and the X-ray diffraction crystal structure (2J2M, 2.40 Å) of the CAT protein of *Exiguobacterium oxidotolerans* was selected as the template for homology modeling based on the search results. Prediction of CAT1 protein homology tetramer structure was accomplished using single template modeling by Modeller [39] with parameters set to predict 100 models and select the optimal model, using DOPE and GA341 scoring models. The quality of the constructed models was evaluated using the SAVES v6.0 [40] (https://saves.mbi.ucla.edu/ (accessed on 12 March 2023)), and the percentage greater than 90% of the amino acid residues falling in the yellow and red regions was considered to qualify the protein structure model. Homologous structure comparison and 3D structure display were performed using UCSF Chimera (1.16) [41] and VMD (1.9.4) [42]. Molecular docking and visualization display were provided using Autodock [43]. We obtained the molecular structure of H_2_O_2_ (7722-84-1) from the PubChem compound database (https://pubchem.ncbi.nlm.nih.gov/ (accessed on 25 March 2023)). Polypeptide chain amino acid residue hydrophilicity was predicted using ProtScale (https://web.expasy.org/protscale/ (accessed on 25 April 2023)), and amino acid residue hydrophilicity was evaluated using Hphob./Kyte & Doolittle method. Potential positive selection sites were predicted for fixed-point mutations, using the online tool Missense3D [44] (http://missense3d.bc.ic.ac.uk/missense3d/ (accessed on 26 April 2023)).

## 3. Results

### 3.1. Gene Identification Results

The results of gene identification showed (Table S2) that all Chenopodiaceae’s *SOD* gene families consisted of two *FSD*, four *CSD* and one *MSD* genes, whereas the composition of the *SOD* gene family members in Caryophyllaceae varied considerably, with the ratios of the *FSD*, *CSD* and *MSD* compositions of the *SOD* gene families of *S. uniflora* and *H. pusillum* being 2:3:2 and 2:3:1, respectively. Seven *SOD* genes, two *FSD*, three *CSD* and two *MSD*, were expressed in the leaf tissues of *G. przewalskii*. The identification of the *APX* gene family members showed (Table S2) that the genomes of the Chenopodiaceae *B. vulgaris*, *S. oleracea* and *C. pallidicaule* contained twelve, eleven and ten *APX* gene family members, respectively, and the genomes of the Caryophyllaceae plants *H. pusillum* and *S. uniflora* contained eleven and ten *APX* gene family members, respectively, whereas a total of six *APX* genes were identified to be expressed in the transcriptome of *G. przewalskii* leaf tissues. The genome of *H. pusillum* contained five *CAT* genes, and the genomes of all other Centrospermae plants contained two *CAT* genes (Table S2).

### 3.2. Genetic Relationship, Gene Structure, Motifs and Domains

**Figure 1:**
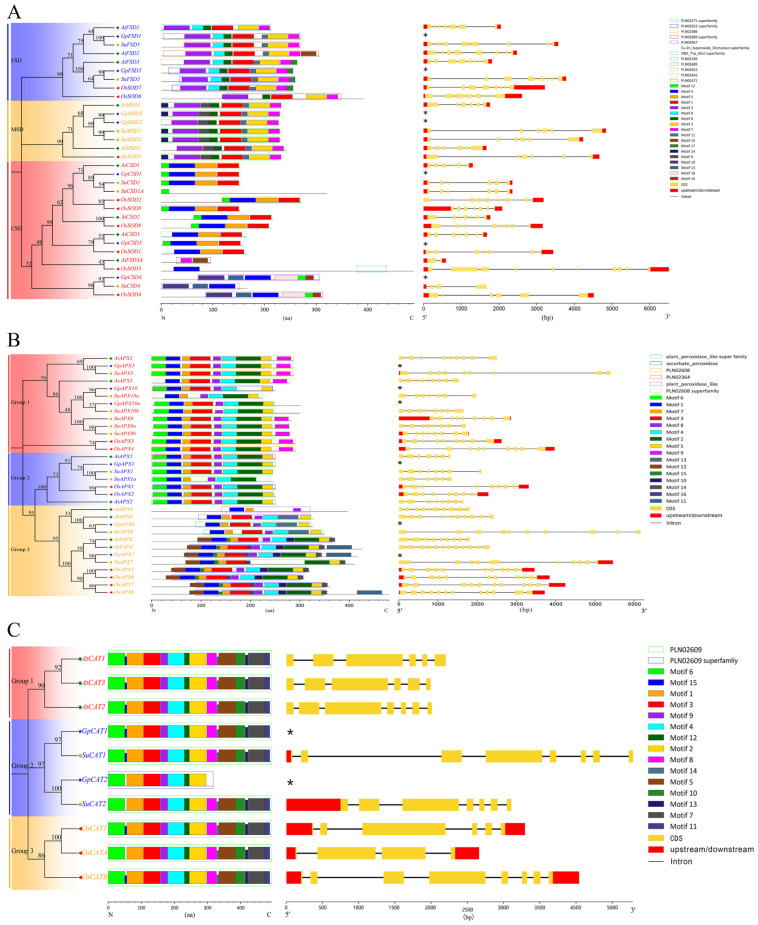
Phylogenetic relationships, gene structure, motifs and domains of the identified genes. (**A**–**C**) show the phylogenetic relationships, gene structure, motifs and domains of SOD, APX and CAT, respectively. * indicates no genetic structure.

In this study, we constructed the phylogenetic trees of the *SOD*, *APX* and *CAT* gene families of *A. thaliana*, *O. sativa*, *S. uniflora* and *G. przewalskii* and analyzed their gene structures, motifs and domains. The results showed that the *SOD* genes of the four plants were clustered into three major branches (Figure 1A). According to the results of previous studies, the *SOD* gene family was divided into three subfamilies, *FSD*, *CSD* and *MSD*, based on the type of metal ions in their active centers, and thus the three branches were defined as *FSD*, *CSD* and *MSD*, respectively, based on the clustering results of the evolutionary tree. The composition ratios of the three subfamilies, *FSD*, *CSD* and *MSD*, of the *SOD* gene families of *A. thaliana*, *O. sativa*, *S. uniflora* and *G. przewalskii* were 3:2:4, 2:1:5, 2:2:3 and 2:2:3, respectively, suggesting that there is a large variation in the composition ratios of the three subfamilies in different plant lineages. The motif and domain analyses showed that motifs and domains of the members of the same subfamily in different plant species were conserved; for example, in the *MSD* subfamily, all members contained Motif 3, Motif 9, Motif 6, Motif 1, Motif 11, Motif 2 and Motif 7, and the relative positions of the seven motifs were the same. The results of the gene structure analysis showed that the members of the same subfamily have a similar or the same number of exons and introns, for example, in the *FSD* subfamily, *AtFSD3* contains eight exons and seven introns, and *SuFSD3* and *OsSOD7*, which are directly homologous to it, also contain the same number of exons and introns. However, *AtFSD1* contains seven exons and six introns, and SuFSD1 contains eight exons and seven introns. According to the clustering results of the phylogenetic tree, the *G. przewalskii’s SOD* genes are closely related to the *S. uniflora’s SOD* genes, and therefore it was inferred that the gene structure of the *G. przewalskii’s SOD* genes is similar to that of the Seaside Flycatcher’s direct homologous genes.

The evolutionary relationships, gene structures, motifs and domains of the *APX* gene family are shown in Figure 1B. The *APX* genes of the four plants were clustered into three major branches, Group 1, Group 2 and Group 3, with high Bootstrap (>70). The *APX* genes of both *G. przewalskii* and *S. uniflora* were clustered together with those of *A. thaliana*, suggesting that the *APX* genes of Caryophyllaceae plants are more closely related to the *APX* genes of *A. thaliana*. Gene structures, motifs and domains are conserved, and genes on the same evolutionary branch have similar or identical gene structures, motifs and domains.

The evolutionary relationships, gene structures, motifs and domains of the *CAT* gene family are shown in Figure 1C. The *CAT* genes of the four plants were clustered into three major branches, namely Group 1, Group 2 and Group 3. In contrast to the evolutionary relationships of the *SOD* and *APX* gene families, the *GpCAT* and *SuCAT* genes are distantly related to both the *AtCAT* and *OsCAT* genes, which are clustered into a single unit (Group 2). Except for *GpCAT2*, which contains only seven motifs, the rest of the *CAT* genes contain fourteen to fifteen motifs, and all of them contain the PLN02609 structural domain. The gene structure analysis showed that *SuCAT1* and *SuCAT2*, which are directly homologous to *GpCAT1* and *GpCAT2*, have an “8-exon + 7-intron” and ”7-exon + 6-intron” structure, respectively, which is different from the gene structures of *AtCAT* and *OsCAT*.

### 3.3. Gene Function Annotation and Protein Interaction Prediction

**Figure 2:**
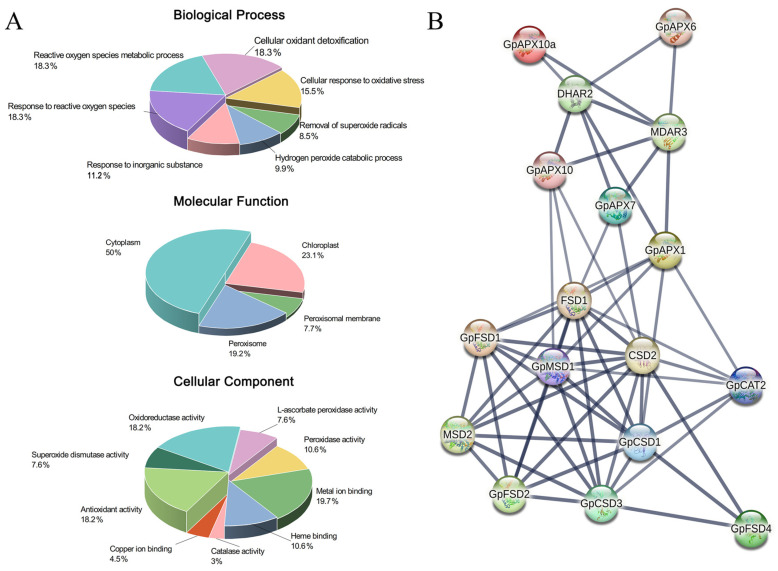
Gene function annotation results (**A**) and protein interaction prediction results (**B**) of the identified genes.

Gene annotation results showed (Figure 2A) that *GpSOD*, *GpAPX* and *GpCAT* were mainly involved in seven biological processes including removal of superoxide radicals (GO:0019430), cellular response to oxidative stress (GO:0034599) and reactive oxygen species metabolic process (GO:0072593). MF annotation results showed that these genes have catalase activity (GO:0004096), superoxide dismutase activity (GO:0004784) and peroxidase activity (GO:0004601). The CC enrichment analysis confirmed that these genes were mainly associated with the cellular components of cytoplasm (GO:0005737), chloroplast (GO:0009507) and peroxisome (GO: 0005777) and other cellular components. The results of the protein interaction prediction showed that there is a very significant interaction among the *GpSOD*, *GpAPX* and *GpCAT* genes (Figure 2B). The above results demonstrated the major role of the antioxidant enzyme genes in response to oxidative stress in *G. przewalskii*.

### 3.4. Physical and Chemical Properties and Subcellular Localization of the Proteins

The analysis of the physicochemical properties of the proteins (Table S3) showed that the *GpSOD* genes encoded 152–306 amino acids with molecular weights ranging from 15109.74 to 32884.70 and isoelectric points ranging from 5.47 to8.94. The GpSOD proteins were all hydrophilic proteins, and most of them were unstable (instability coefficients >30). Subcellular localization showed that the *GpSOD* genes were mainly expressed in mitochondria and chloroplasts. The *GpAPX* genes encoded 250–406 amino acids with molecular weights ranging from 27,488.09 to 44,289.24, isoelectric points ranging from 5.42 to 9.08, and most were alkaline proteins. The GpAPX proteins were all hydrophilic and unstable proteins and were expressed in cytoplasm, peroxisomes, mitochondria and chloroplasts. The two members of the *GpCAT* gene family encode 492 and 317 amino acids, with molecular weights of 56,697.08 and 36,386.21, respectively, and are neutral proteins with high hydrophilicity but poor stability, both are mainly expressed in peroxisomes.

### 3.5. Gene Expression and RT-qPCR Analysis

**Figure 3:**
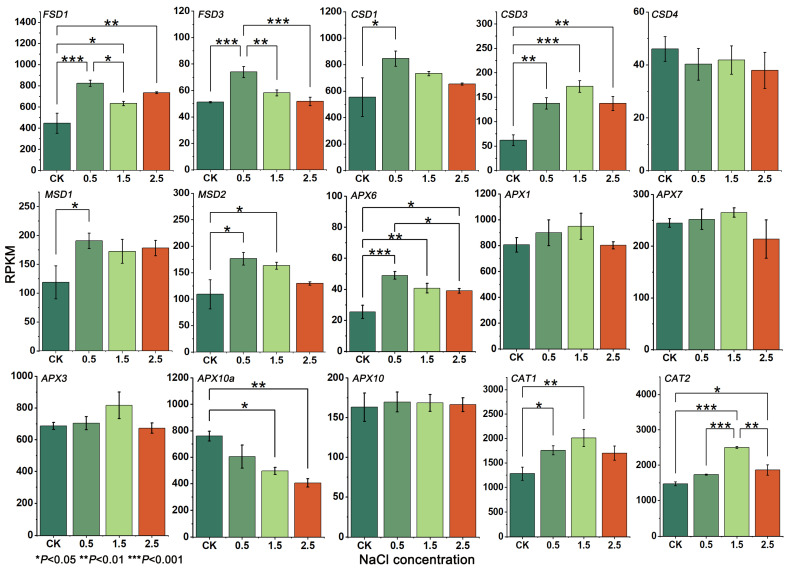
Expression levels (*Y*-axis) of *SOD*, *APX* and *CAT* genes in leaves of *G. przewalskii* treated with different concentrations (%) of NaCl (*X*-axis).

In this study, the expression of *GpSOD*, *GpAPX* and *GpCAT* was analyzed using transcriptomic data from leaf tissues under different NaCl concentrations. The results showed that different gene families of the antioxidant enzyme system of *G. przewalskii* had different modes of regulation under NaCl stress (Figure 3). The analysis of *GpSOD* expression showed that *GpFSD1* and *GpCSD1* had the highest expression, and *GpFSD2* and *GpCSD4* had the lowest. With the increase in NaCl concentration, the *GpCSD1* expression showed a trend of increasing and then decreasing and reached the highest value at a NaCl concentration of 0.5%, which was significantly (*p* < 0.05) higher than that of CK by 52.53%. The regulation pattern of *GpFSD1* was significantly different from that of *GpCSD1*, and the expression of *GpFSD1* showed an up-down-up regulation when stressed by NaCl, where the expression of *GpFSD1* at a NaCl concentration of 0.5% was significantly higher (*p* < 0.05) than that of CK (*p* < 0.05), about 1.84 times higher. *GpCSD3* showed a different expression pattern compared to that of *GpMSD1* and *GpMSD2*, in which the expression of *GpCSD3* was consistently upregulated for CK and a 1.5% NaCl treatment, reaching the maximum at 1.5%, which was significantly (*p* < 0.05) higher than the expression in CK. The expression pattern of *GpMSD1* and *GpMSD2* was similar to that of *GpCSD1*, both showing upregulation followed by continuous downregulation along with an increased NaCl concentration, while *GpFSD2* and *GpCSD4* had the lowest expression, which indicted lower sensitivity to salt stress conditions, and they had no significant difference in the expressions at the four different NaCl concentrations (*p* > 0.05).

The *GpAPX* expression analysis (Figure 3 showed that *GpAPX1*, *GpAPX3* and *GpAPX10a* had the highest expression for CK, among which *GpAPX1* and *GpAPX3* showed a response pattern of continuous upregulation followed by downregulation with the increase in NaCl concentration, and their expression reached the maximum at a 1.5% NaCl treatment, where they were upregulated by 17.87% and 18.92% compared to CK, respectively, but the differences were not significant (*p* > 0.05). The response pattern of *GpAPX10a* was different from that of *GpAPX1* and *GpAPX3*, was consistently downregulated after being subjected to NaCl stress, reaching the lowest when NaCl concentration was 2.5%, with a reduction of 46.58% compared with CK. *GpAPX7* and *GpAPX10* were highly expressed, but not significantly, which might be constitutively expressed in the leaf tissues of *G. przewalskii*. *GpAPX6* had the lowest expression, and with the increase in NaCl concentration, it was firstly upregulated then continuously downregulated, and it reached the maximum at a 0.5% NaCl treatment, which was significantly higher than that of CK (*p* < 0.05). The above results indicated that the members of the *GpAPX* family played different regulatory roles under NaCl stress, and these genes were responsible for maintaining ROS homeostasis in leaf tissues under NaCl stress.

Compared with *GpSOD* and *GpAPX*, all *GpCAT* genes showed the expression trend of continuous upregulation and then downregulation after being stressed by NaCl (Figure 3), and the *GpCAT1* and *GpCAT2* showed the highest expression. The expression of *GpCAT1* was significantly higher (1.57 times) than that in CK at the concentration of 1.5% NaCl and slightly higher than that in CK when the concentration of NaCl reached 2.5%. Similar to the response pattern of *GpCAT1*, the expression of *GpCAT2* was higher than that in CK when the concentration of NaCl reached 2.5%. When the NaCl concentration was 1.5%, the expression of *GpCAT2* was upregulated by 51.09% compared with that of CK. When the NaCl concentration continued to increase, the expression of *GpCAT2* started to decrease from the peak, but it was still higher than that of CK. The above results indicated that the expression of *GpCAT1* and *GpCAT2* are inducible and sensitive to salt stress, and their high expression in CK and upregulation under NaCl stress may play a crucial role in maintaining ROS homeostasis in the leaves of *G. przewalskii*.

**Figure 4:**
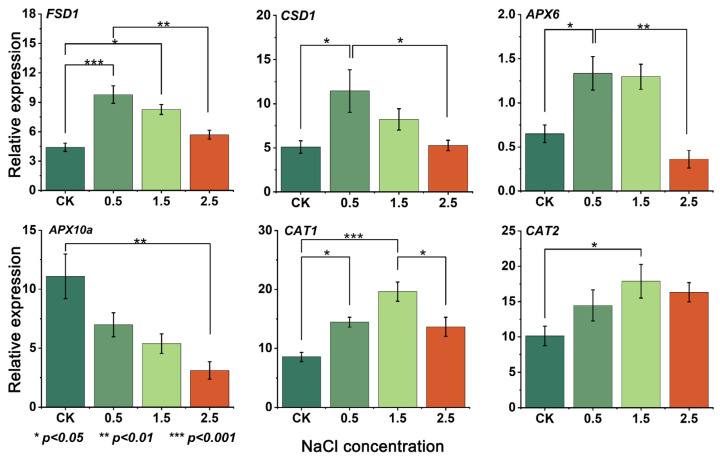
RT-qPCR analysis of antioxidant enzyme genes in *G. przewalskii* under NaCl stress. *X*-axis indicates NaCl concentration (%), while *Y*-axis indicates relative expression level.

To verify the accuracy of the transcriptome data, the RT-qPCR analysis of the *GpFSD1*, *GpCSD1*, *GpAPX6*, *GpAPX10a*, *GpCAT1* and *GpCAT2* genes was performed in this study. The results showed that the expression trends of all six genes were consistent with the transcriptome expression (Figure 4), indicating that the transcriptome data were accurate and credible.

### 3.6. Changes in SOD, APX and CAT Enzyme Activities

**Figure 5:**
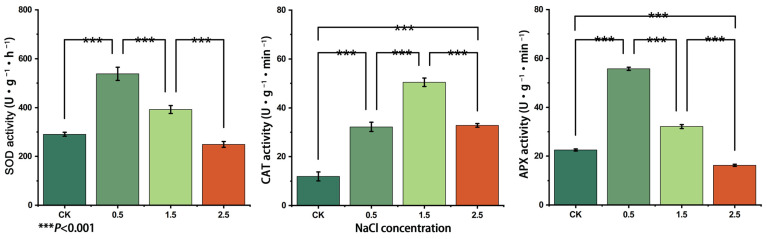
Activities of SOD, APX and CAT enzymes in leaves of *G. przewalskii* treated with different concentrations (%) of NaCl.

The results of enzyme activities of SOD, APX and CAT under NaCl stress (Figure 5) showed that the activities of SOD and APX both reached the maximum value when the NaCl concentration was 0.5%, after which the enzyme activities continued to decrease with the increase in NaCl concentration. When the NaCl concentration reached 2.5%, both SOD and APX activities were lower than CK, indicating that the NaCl concentration at this time had exceeded the range of action of SOD and APX. CAT activity reached the maximum value when the NaCl concentration was 1.5%, and at this time, the enzyme activity was 3.92 times higher than that of CK, and the CAT activity was reduced when the NaCl concentration reached 2.5%, although its activity was still significantly higher than that of CK, indicating that CAT has higher tolerance to NaCl stress compared with SOD and APX. The correlation analysis showed a significant positive correlation between gene expression and enzyme activity (Table S4).

### 3.7. Molecular Evolutionary Analysis

**Figure 6:**
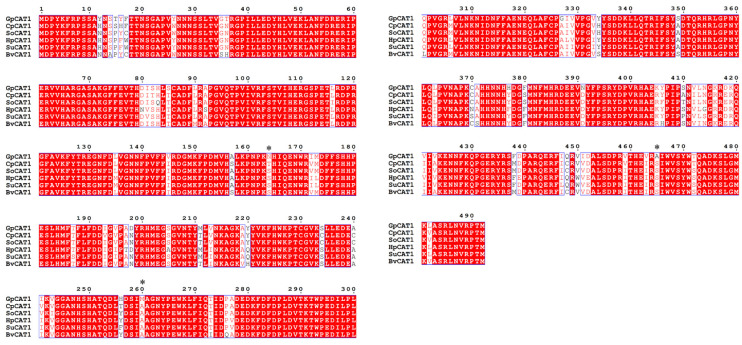
Nucleotide sequence comparison of *CAT1* gene in Centrospermae plants (* indicates a positive selection site).

In this study, the 15 genes identified were analyzed for positive selection pressure, using the site model, branch model and branch-site model of PamL. The results of the locus model (Table S5) showed that the 15 antioxidant enzyme genes from six central seed order plants had different evolutionary rates (M0 model), and all of them were subjected to purification (ω < 1). The results of the M8 model suggest that a few sites in the *APX1*, *APX7*, *APX10*, *APX10a*, *CSD3*, *CSD4*, *FSD1*, *MSD1*, *MSD2*, *CAT1* and *CAT2* genes may be subject to selective pressures from the environment. In the branch model, *G. przewalskii* branch was set as the foreground branch (two-ratio model), and the *GpAPX6*, *GpAPX7*, *GpAPX10*, *GpAPX10a*, *GpCSD4*, *GpMSD1*, *GpMSD2*, *GpCAT1* and *GpCAT2* genes showed a greater evolutionary rate than that of the background branch (ω > ω_0_), and the LRT results showed that there might be a positive selection site for the *GpCAT1* gene (*p* ≤ 0.05). In order to accurately detect the loci of the *GpCAT1* and *GpCAT2* genes under positive selection pressure in naked fruit trees, *G. przewalskii* were set as foreground branches in the branch-site model to detect the few positive selection loci present in the *CAT1* gene. The results showed that the *GpCAT1* gene loci 164 N, 260 M and 465 A were under strong positive selection pressure (Figure 6), with significant LRT results (*p* = 0.03) and high BEB posterior probability confidence levels of 0.950, 0.969 and 0.954, respectively.

### 3.8. Protein Model Construction and Quality Evaluation

To explore whether the presence of mutation sites in GpCAT1 has an effect on the protein structure, we predicted the common ancestor sequence of CAT1 in Caryophyllaceae species and comparatively analyzed the physicochemical properties, active center properties and affinity for H_2_O_2_ binding of GpCAT1 and AsCAT1 proteins. According to the SWISS-MODEL search results and the corresponding protein structure files, the currently available CAT protein tertiary structures (e.g., 4qol, 1e93 and 1h6n) lacked the N-terminal 14 amino acid residue coordinate information, and it was hypothesized that there was a post-translational modification mechanism for CAT. Therefore, the predicted protein tertiary structures of GpCAT1 and AsCAT1 in this study include only amino acid residues 15–478.

The quality of protein models determines the accuracy of their structural and functional analyses. The results of SAVESv6.0 evaluation (Figure S1) showed that 92.2% and 92.6% of the amino acid residues of the GpCAT1 and AsCAT1 protein models, respectively, were in the qualified region, and thus it was concluded that the protein models constructed in this study were of high quality and could be used for the subsequent analyses such as structural comparison and molecular docking.

#### 3.8.1. Comparison of Physical and Chemical Properties of Proteins

The comparison of the physicochemical properties of the proteins showed that both GpCAT1 proteins had higher hydrophilicity and stability compared to AsCAT1 proteins, with hydrophilicity of GpCAT1 and AsCAT1 proteins being −0.522 and −0.518 and instability indexes of GpCAT1 and AsCAT1 proteins being 31.32 and 43.67, respectively (Table 1).

#### 3.8.2. Property Analysis of Active Center

The GpCAT1 protein active center had a stronger hydrophilicity and larger volume compared to that of the AsCAT1 protein, with the former having a hydrophilicity and volume of 0.73 and 0.227 nm^3^, respectively, and the latter having a hydrophilicity and volume of 0.72 and 0.19 nm^3^, respectively (Table 2).

#### 3.8.3. Protein Molecular Docking

**Figure 7:**
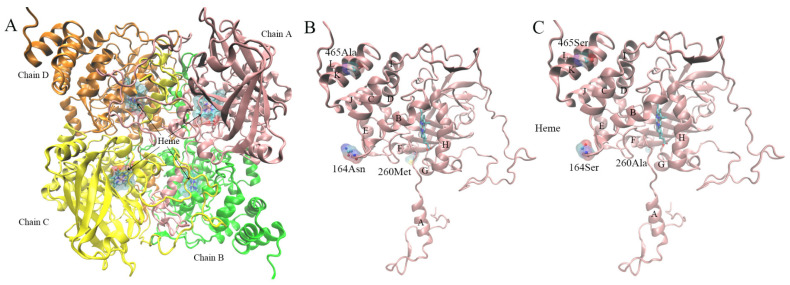
Protein 3D structure diagram. (**A**) is a 3D structural diagram of protein of GpCAT1 and AsCAT1, and (**B**,**C**) are schematic diagrams of mutation sites of GpCAT1 and AsCAT1 protein, respectively. Small letters of A to L in (**B**,**C**) are spiral structures on subunit A of CAT protein.

**Figure 8:**
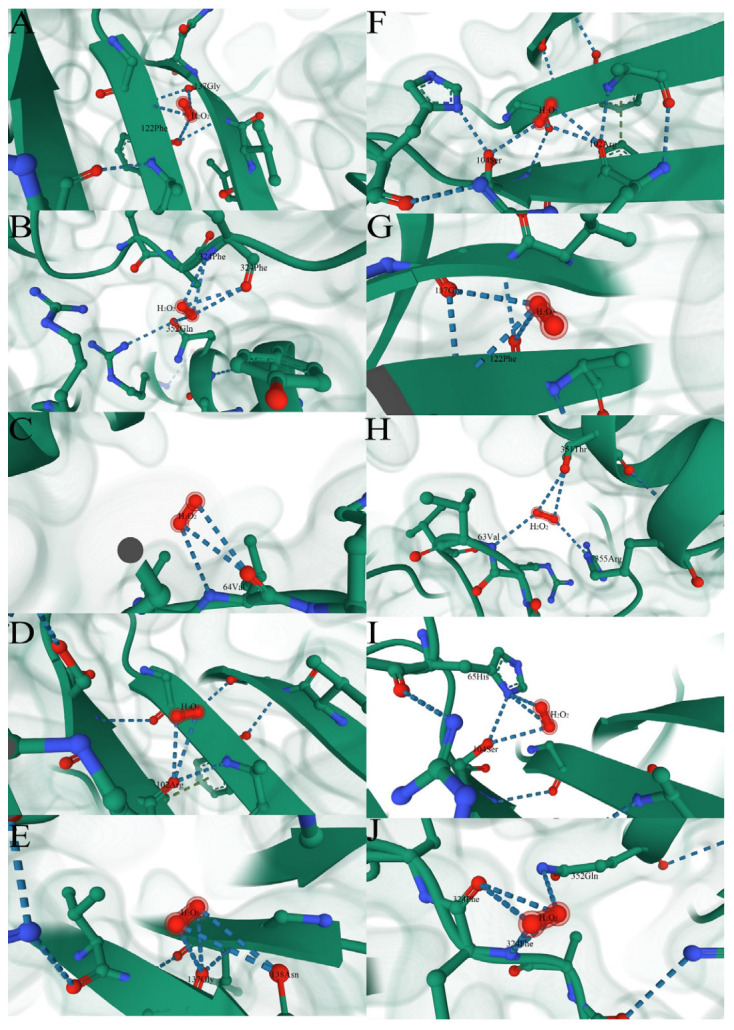
Binding site of CAT1 protein and H_2_O_2_. (**A**–**E**) are the conformations of the first five sites of AsCAT protein binding to H_2_O_2_. (**F**–**J**) are the conformations of the first five sites of AsCAT protein binding to H_2_O_2_.

The comparison of protein structures showed that a few of the positively selected mutation sites of the GpCAT1 protein are located in positions near the active center (near the heme pocket) (Figure 7B,C). Therefore, in this study, we predicted the binding sites of the GpCAT1 and AsCAT1 proteins for H_2_O_2_, and comparatively analyzed the binding energies of H_2_O_2_ with the proteins. The results showed that H_2_O_2_ has nine possible binding sites with CAT proteins, all of which are located in the active center of CAT proteins, and all of which bind to CAT proteins through visible hydrogen bonds and strong electrostatic interactions (Figure 8). In addition, the binding energies of the seven sites of GpCAT1 with H_2_O_2_ were greater than those of AsCAT with H_2_O_2_ (Table S8), indicating that GpCAT1 has a higher affinity for H_2_O_2_.

#### 3.8.4. Prediction of Site Mutation and Changes in Protein Conformation

**Figure 9:**
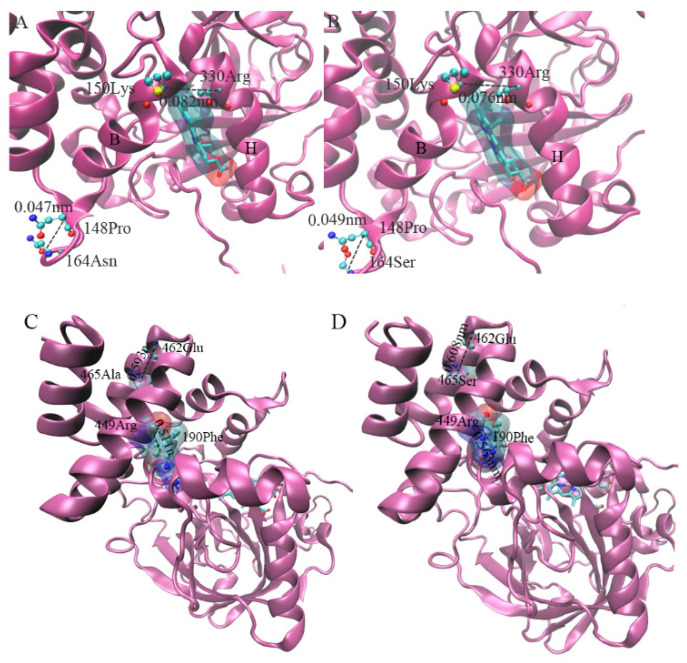
Mutations at amino acid positions 164th and 465th in protein of GpCAT1 and conformational changes in protein. (**A**,**B**) show the mutation of amino acid site 164 and protein conformation of CAT1 protein in *G. przewalskii* and Ancestral, and (**C**,**D**) show the mutation of amino acid site 465 and protein conformation of CAT1 protein in *G. przewalskii* and Ancestral.

The function of proteins is directly related to their structures. In order to elucidate how the above potential positive selection site of the *GpCAT1* gene causes changes in phenotypes such as increased volume of the active center of proteins and higher hydrophilicity through changes in protein structure, this study analyzed the potential positive selection site of the *GpCAT1* gene through amino acid site mutation and a protein structure comparison. The results showed that the mutation at amino acid position 164 of the GpCAT1 protein resulted in the addition of one hydrogen bond between amino acid 164 and amino acid 161, and this force led to the deflection of the B-helix of the nudibranch CAT1 protein in the direction away from the H-helix (the Cα distance between amino acid positions 164 and 148 of the nudibranch and ancestral CAT1 proteins was detected to be 0.047 nm and 0.049 nm), resulting in an increase in the angle between the B- and H-helices. The distance between amino acid 150 in the B-helix and amino acid 330 in the H-helix increased from 0.076 nm in the ancestral the CAT1 protein to 0.082 nm in Naked Nutmeg (Figure 9A,B). Both the B- and H-helices are located at the heme pocket, which is very favorable for increasing the volume of the heme pocket.

**Figure 10:**
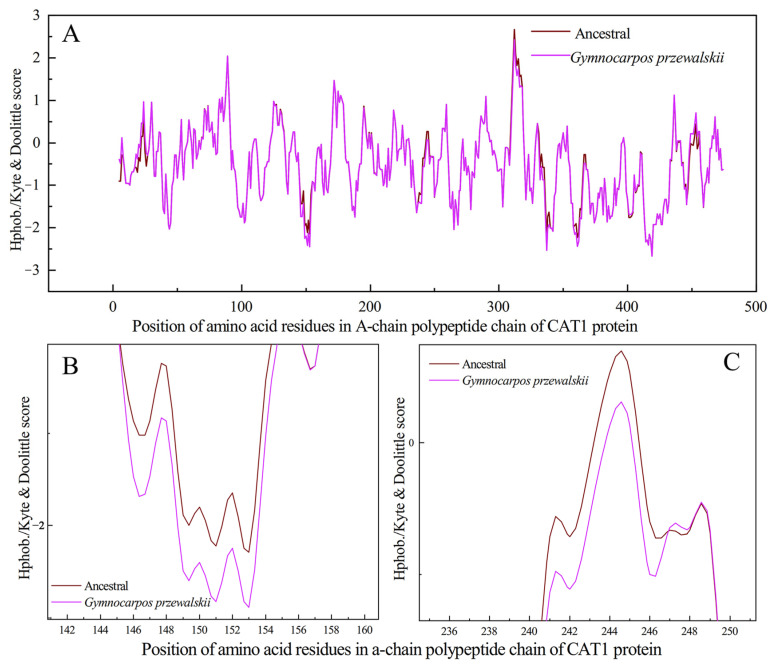
Hydrophilicity distribution of GpCAT1 and AsCAT1 polypeptide chains. (**A**) shows the hydrophilic distribution of amino acid residues in the ancestral CAT1 polypeptide chain of *G. przewalskii* and Ancestral. (**B**,**C**) show the hydrophilic distribution of peptides at amino acid residues 164 and 260 in the ancestral CAT1 polypeptide chain of *G. przewalskii* and Ancestral, respectively.

The single-point mutation results showed that the mutation of amino acid 465 of GpCAT1 resulted in a volume increase of 0.059 nm^3^. Structural comparisons revealed that this mutation enhanced van der Waals forces between neighboring atoms (the distance between amino acid 465 to amino acid 462 was shortened from 0.608 nm in AsCAT1 to 0.593 nm in GpCAT1), and this force may be along the main chain carbon atom conduction, which ultimately led to the shortening of the distance between the J-helix and K-helix from 0.563 nm in AsCAT1 to 0.511 nm in GpCAT1. The D-helix is the main helical structure forming the hemoglobin pocket, and thus this mutation favored the increase in the size of the hemoglobin pocket (Figure 9C,D). In addition, compared with the AsCAT1 protein, amino acids 164 and 260 of the GpCAT1 protein were mutated from hydrophobic amino acids to hydrophilic amino acids, which increased the hydrophilicity of peptide chains in the corresponding region of the protein, which may be the main reason for the increased hydrophilicity of the protein of GpCAT1, and it is favorable for the increase in the solubility of the protein in the solution (Figure 10).

## 4. Discussion

### 4.1. Gene identification Results

As one of the most important enzymes for the scavenging of reactive oxygen species, *SOD* plays an important role in the maintenance of cellular homeostasis and antioxidant responses in plants. With the continuous application and development of sequencing technology, the members of the *SOD* gene family have been successively cloned and characterized in species of different taxonomic status. The *SOD* gene family in *Liriodendron chinense* [45], *Vitis vinifera* [46] and *Brassica napus* [47] contains eight, ten and thirty-two members, respectively, with the *FSD*, *CSD* and *MSD* subfamily composition patterns of 2:5:1, 2:6:2 and 10:12:10, respectively. The *GpSOD* gene family contains seven family members, and the three subfamilies, *FSD*, *CSD* and *MSD*, are composed in a 2:3:2 pattern. Because of the tissue-specific expression of the *SOD* genes, it is not possible to determine whether the genes identified in this study contain all the members of the *SOD* gene family of *G. przewalskii*. The plants of Caryophyllaceae and plants of Chenopodiaceae contain six to seven family members of the *SOD* gene family, in which *S. uniflora* of Staphylinidae contains seven *SOD* family genes with a 2:3:2 pattern of subfamily composition of *FSD*, *CSD* and *MSD*, but plants of Staphylinaceae *H. pusillum* has only six *SOD* family genes and lacks *MSD2* compared to Silene uniflora. The plant genomes of Chenopodiaceae all contain seven *SOD* genes, and the three subfamilies have the same compositional pattern of 2:4:1. It is thus demonstrated that, although the *SOD* gene family consists of three subfamilies, the number of family members and the pattern of the number of subfamilies in different taxa or in the same taxon of phytomass are quite different.

Multiple *APX* isozymes exist in plants and play important roles in maintaining the balance of reactive oxygen species metabolism in plants at different subcellular levels [48]. In this study, six members of the *APX* gene family were identified to be expressed in *G. przewalskii* leaf tissue transcripts, and the low number of the *APX* gene families in *G. przewalskii* compared to other plants of Centrospermae may be related to the tissue-specific expression characteristics of the *APX* genes present [8]. The number of plant *APX* gene family members varies greatly among different lineage species, both *A. thaliana* [49] and *O. sativa* [50] genomes contain eight *APX* gene family members, while recent studies have shown that *Populus trichocarpa* [51] genome contains eleven *APX* gene family members, *Actinidia chinensis* [52] genome contains twenty-nine *APX* gene family members, and this study found that the plant genome of Centrospermae contains ten to twelve *APX* gene family members, and the number of gene family members can reflect the diversity of protein function to a certain extent [48]. Therefore, it is inferred that the species of Centrospermae may have more complex ascorbic acid metabolic pathways. The results of the phylogenetic relationship reconstruction and gene structure analysis showed that the same isoforms of the *APX* gene have similar gene structures, which is consistent with the findings for most plant *APX* genes, suggesting that the *APX* genes are evolutionarily conserved in plants, and although gene duplication and deletion events occur in some plants, resulting in differences in the number of genes between species of the same lineage, in general, the structure of the *GpAPX* genes is more stable and is subject to less environmental pressures, and the members of the *APX* gene family are subjected to purifying selection in most plants [51].

The *CAT* gene family in plants consists of a small number of genes compared to the *SOD* and *APX* gene families. *A. thaliana* [53], *O. sativa* [54] and *Zea mays* [55] each have three *CAT* genes, and *Hordeum vulgare* has two *CAT* genes [56]. While the genome of *Brassica napus* contains 14 *CAT* genes, *B. napus* [57] is an allotetraploid type that undergoes extensive genome doubling and integration events. In this study, a total of two *GpCAT* genes were identified as expressed from the leaf tissues of *G. przewalskii*, and the genomes of plants of Centrospermae contained two *CAT* genes except for the genome of *S. uniflora* which contained five *CAT* genes, which was found to be heavily fragmented during the identification process of *S. uniflora*, so for the five *CAT* genes of *S. uniflora* identified in this paper, more perfect genomic validation is needed.

### 4.2. Expression Patterns of Antioxidant Enzyme-Related Genes in Leaf Tissues

Plant ROS are mainly formed in organelles such as chloroplasts, mitochondria and peroxisomes, and according to the different forms of oxygen present, ROS are generally categorized as superoxide radical (O^2•−^), hydrogen peroxide (H_2_O_2_) and hydroxyl radical (-OH) and singlet linear oxygen (1O_2_) [6]. The pathways of plant O^2•−^ production mainly include leakage of electrons from the electron transport chain (ETC) in the photosystem I (PS I) and photosystem II (PS II) pathways on chloroplasts. The pathways of H_2_O_2_ production mainly include electron chain transfer in mitochondria, chloroplasts and fatty acid β-oxidation. -OH production includes various pathways such as H_2_O_2_ via Haber–Weiss reaction and photolysis of H_2_O_2_ with water. Due to the extremely strong oxidizing properties and the absence of specific mechanisms for scavenging -OH in plants, it is extremely damaging to plant cells, so most plants reduce -OH production through a strong H_2_O_2_ scavenging system [58]. The plant antioxidant enzyme system is the first line of defense against free radical damage, which first disproportions O_2_^•−^ to O_2_ and H_2_O_2_ by SOD during stress and then converts H_2_O_2_ to H_2_O and O_2_ by APX, GR, GPX and CAT. Taken together, the complementary and coordinated mechanism of multiple antioxidant enzymes constitutes the plant antioxidant enzyme defense system, which provides a strong defense against free radical damage in the case of providing a protective barrier when subjected to adversity stress. The GO annotation results and protein interactions of the *GpSOD*, *GpAPX* and *GpCAT* genes in this study also confirmed this point.

The subcellular localization studies of the *SOD* gene family members revealed that *FSD* and *CSD* are mainly expressed in chloroplasts, where antioxidant enzyme system plays a key role under high NaCl stress. The overexpression of *FSD* and *CSD* in chloroplasts was found to increase plant tolerance to oxidative stress in a 1994 study of transgenic tobacco [59]. The results of this study indicated that *FSD1* and *CSD1* were the main expressed genes in the leaves of *G. przewalskii* and were the most sensitive to the changes in NaCl stress; the RPKM values for both reached the maximum at a NaCl concentration of 0.5% and were significantly higher than those of CK, 1.84-fold and 1.52-fold, respectively. In addition, *CSD3* and *MSD1* and *MSD2* changed significantly when subjected to NaCl stress, but CSD4 and FSD2 responded more sluggishly to NaCl stress. This may be related to the existence of a spatiotemporal specific expression of *SOD* genes in plants [60]. The above results indicated that the upregulation of the expression of the *FSD1* and *CSD1* genes may enable chloroplasts to photosynthesize normally under salt stress in *G. przewalskii*, which may be the main reason for the elevated SOD activity.

APX is a potent regulator of ROS, and among several peroxidases, APX has the strongest affinity for H_2_O_2_, which is present in a variety of subcellular organelles and can play a scavenging role under a wide range of stresses [61]. Increasing evidence suggests that the majority of the *APX* genes in plant cells are induced by salt stress, and that rapid upregulation of APX at the transcriptional level maintains high *APX* activity in organelles to protect cellular components from ROS-induced oxidative damage [15]. The results of this study showed that *GpAPX1*, *GpAPX3*, *GpAPX6* and *GpAPX7* exhibited different degrees of upregulation under salt stress, and the expression of all these four genes was downregulated at a NaCl concentration of 2.5%, suggesting that *APX1*, *APX3*, *APX6* and *APX7* in the leaf tissues of *G. przewalskii* play a major role in the H_2_O_2_ scavenging under salt stress. However, high concentrations of NaCl had a significant inhibitory effect on them. *APX10* showed no significant change in expression under salt stress, and it was hypothesized that *APX10* was constitutively expressed in the leaf tissues of *G. przewalskii*. Unlike the other *APX* genes, the *GpAPX10a* gene was repressed under NaCl stress, which is consistent with the findings in most plants, for example, a recent study found that the *APX7*, *APX8* and *APX6* genes expressed in the leaf tissues of *P. tomentosa* were significantly downregulated under 150 mM NaCl stress, which indirectly proves that the *APX* genes in response to environmental salt stress diversify the physiological and biochemical functions [53].

An important function of *CAT* is to scavenge H_2_O_2_ produced during photorespiration in leaf tissue, and *CAT* has been shown to exert a significant antagonistic effect on salt stress in a variety of plants. For example, *CAT1* and *CAT2* in *P. trichocarpa* under salt stress showed higher transcript levels in leaves, which enhanced the resistance to salt stress [17]. Eleven expressions were significantly upregulated in oilseed rape under NaCl stress, suggesting that *CAT* plays an important role in the enhancement of salt tolerance in *B. napus* [57]. In this study, we found that *GpCAT1* and *GpCAT2* showed different degrees of upregulation under NaCl stress, and the expressions of *GpCAT1* and *GpCAT2* were 1.57-fold and 1.51-fold higher than those of CK, respectively, at a NaCl concentration of 1.5%, at which that salt stress induces a large amount of expression of *CAT1* and *CAT2* in the leaf tissues of *G. przewalskii,* and the expression is relatively high. With the increase in the NaCl concentration, the expression of both the *GpCAT1* and *GpCAT2* genes was downregulated at a NaCl concentration of 2.5%, indicating that 2.5% NaCl had an obvious inhibitory effect on them. In addition, the higher expression of *GpCAT1* and *GpCAT2* compared to several other enzyme family members may be attributed to the enzymatic properties of *CAT*, which was found to have a low affinity for H_2_O_2_, so that a large amount of *CAT* was required to achieve the removal of excess H_2_O_2_ [62].

In summary, the transcriptome expression analysis revealed that most of the *GpSOD*, *GpAPX* and *GpCAT* genes were upregulated to varying degrees under salt stress, which may be one of the reasons for the strong salt tolerance of *G. przewalskii*. In addition, there was no significant decrease in the maximum net photosynthetic rate of *G. przewalskii* leaves under low concentrations of salt stress (NaCl ≤ 0.4%) [24]. In this study, we found that genes such as *GpFSD1* and *GpCSD1* localized in chloroplasts were always maintained at a high expression level, both under normal physiological conditions and under NaCl stress. Therefore, it is hypothesized that protecting the normal physiological function of chloroplasts is one of the characteristics of salt stress adaptation in *G. przewalskii*.

### 4.3. Effect of Positive Selection Mutation Site of CAT1 Gene on Protein Structure

Heredity, variation and selection are the main drivers of biological evolution [63]. The results of the molecular evolutionary analysis showed that three amino acid sites of the *GpCAT1* gene were under strong positive selection pressure. The comparison of protein structures showed that mutations at potential positive selection sites in the *GpCAT1* gene resulted in increased affinity of the protein for H_2_O_2_ binding, increased heme pocket size and hydrophilicity and elevated protein hydrophilicity. Alfonso-Prieto [64] et al. used molecular dynamics to model the mechanism of the action of CAT from *Helicobacter pylori* and catalase Cpd I from *Penicillium vitale* with the substrate H_2_O_2_. The Cpd I–H_2_O_2_ complex was found to form a Cpd II complex by transferring a hydrogen atom from hydrogen peroxide to ferrous ions, during which H_2_O_2_ may diffuse into the active center through the protein channel, and thus the increased volume of the protein channel and the active center has a positive effect on enhancing protein activity. Elevated protein–substrate affinity can effectively increase the catalytic activity of the enzyme [65]. The molecular docking results in this study showed that the GpCAT1 protein has higher affinity for H_2_O_2_, so it is hypothesized that the significant elevation of CAT enzyme activity in the leaf tissues of *G. przewalskii* under NaCl stress is related to its mutation site. On the one hand, the elevated hydrophilicity of the protein caused by the mutations will lead to an increase in the solubility of the protein in solution, which is conducive to the accumulation of the protein in subcellular cells; on the other hand, the elevated hydrophilicity and the increase in the volume of the protein’s active center will increase the chances of collision between the substrate, H_2_O_2_ and the active center, which is advantageous for the protein and the two phases of the reaction with H_2_O_2_.

### 4.4. Effect of Positive Selection Mutation Site of CAT1 Gene on Protein Structure

The *GpCAT1* gene seems to have a very important role in the adaptation of *G. przewalskii* to adversity. Firstly, among the three gene families, *GpSOD*, *GpAPX* and *GpCAT*, *GpCAT1* had higher expression under normal physiological conditions and was upregulated at a higher fold under NaCl stress. Secondly, mutations in the GpCAT1 protein locus resulted in an increased affinity for H_2_O_2_. This is consistent with the fact that CAT activity was increased by 4.24-fold under 1.5% NaCl treatment. Compared to salt-sensitive varieties, salt-tolerant varieties CAT enzyme activity increased with increasing salt stress, and this increase was more pronounced in salt-tolerant genotypes [66]. Although the results of this study cannot prove that the elevated CAT enzyme activity of *G. przewalskii* is much higher than that of the salt-sensitive species, we can affirm that the elevation of *G. przewalskii* CAT at the transcriptional level and the increase in the protein affinity for the substrate are beneficial for the survival of *G. przewalskii* under salt stress. As mentioned earlier, most plants reduce -OH production through a robust H_2_O_2-_scavenging system, which reduces oxidative damage to cells by -OH scavenging. Therefore, we hypothesize that *G. przewalskii* can reduce H_2_O_2_ and -OH production through the CAT enzyme, which allows *G. przewalskii* to survive in high-salinity environments.

In summary, the upregulation of the *APX*, *SOD* and *CAT* genes and the changes in the protein structure of CAT1 led to an increase in the activity of their corresponding proteins, which reduced the oxidative damage suffered by the cells of *G. przewalskii* under salt stress and allowed *G. przewalskii* to continue to survive under salt stress.

## 5. Conclusions

**Figure 11:**
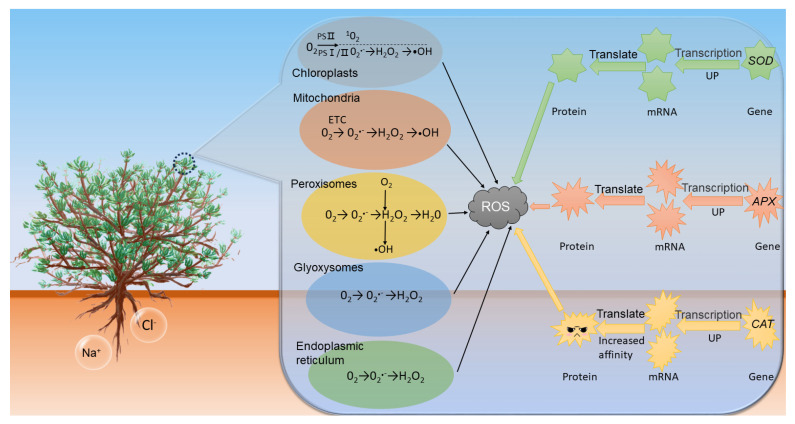
Mechanism of adaptation of antioxidant enzyme system to NaCl stress in leaves of *G. przewalskii.*

In this study, a total of fifteen genes were identified as expressed in the transcriptome of leaf tissues of *G. przewalskii*, including seven *SOD*, six *APX* and two *CAT* genes. Most of the 15 antioxidant enzyme genes showed upregulation under salt stress, suggesting that transcriptional regulation is one of the main strategies of the antioxidant enzyme system of *G. przewalskii* in response to salt stress. The *GpCAT1* gene was subjected to strong positive selection pressure, suggesting that adaptation to salt stress in *G. przewalskii* also involves adaptive evolution of the genetic base material (Figure 11). This study provides new insights for a better understanding of the evolution and function of the *SOD*, *APX* and *CAT* gene families in *G. przewalskii* and their important roles under salt stress.

## Figures and Tables

**Table 1:** Comparison of physicochemical properties between GpCAT1 and AsCAT1 polypeptide chains.

Polypeptide	Number of Amino Acids	Molecular Weight	Isoelectric Point	Average Hydrophilicity	Instability Index
GpCAT1	478	55,052.26	6.75	−0.522	31.32
AsCAT1	478	55,163.38	6.78	−0.518	34.67

**Table 2:** Physical and chemical properties of protein active centers of GpCAT1 and AsCAT1.

Properties	GpCAT1	AsCAT1
Depth	15.47 Å	14.4 Å
Hydrophobicity	−0.73	−0.72
Metal	1	1
Surface	4.18 Å^2^	3.90 Å^2^
Volume	227.33 Å^3^	186.88 Å^3^

## Data Availability

Data is contained within the article and Supplementary Materials.

## Supplementary Materials

The following supporting information can be downloaded at: https://www.mdpi.com/article/10.3390/plants12193370/s1, Table S1: qRT-PCR primer information and reaction system; Table S2: Gene identification results; Table S3: Physical and chemical properties of protein; Table S4: Correlation analysis between gene expression and enzyme activity; Table S5: Molecular evolutionary analysis; Table S6: Molecular docking; Figuer S1: Quality evaluation of protein model.
