# Supplementary material for: Molecular Mechanism of Response and Adaptation of Antioxidant Enzyme System to Salt Stress in Leaves of Gymnocarpos przewalskii
Source: Plants (Basel). 2023 Sep 25;12(19):3370. doi: 10.3390/plants12193370 (PMC10574792; doi:10.3390/plants12193370)
Supplement: Supplementary file 1 [file plants-12-03370-s001.zip › CDS sequence of the gene identified in this paper.pdf]

CDS sequence of SOD, APX and CAT gene family members in *Gymnocarpos przewalskii*

>GpFSD2
ATGAGTATAACCTCACCTTTTCATGCTGCTGCTTTTATTCCCTCAATTAAATTTCCAAGTC
CTTCTTTTCAGCAGAAAAGACGACATCAGGGACTGAGAAGAGGGACTAAAGTTATTGC
TTATTACGGCCTTAAAAAACCTCCATATAACCTGGATGCATTAGAACCATATATGAGCCA
GAAAACACTTGAGTCACACTGGGGGGAGCATCATAAGGGCTACCTGGATGCTCTAAAC
ATACACTTAAGTAGGAGCGACATATTATATGGCTATACCATGGATGAACTTGTGAAAGTG
ACTTATAACTATGGAAATCCTTTGCCTGAATTTAATGCTGCGGCACAGGTCTGGAATCA
CGACTTTTTCTGGGAGTCCATGCAACCTGGAGGCGGGGATTTACCAAAGCTTGGACTT
CTTCAGAAAATTGAGAAAGATTTTGGTTCTTTTGCGAACTTTAAGGAGAAGTTTATAGA
GGCAGCCGTGACATTATTTGGCTCTGGCTGGATTTGGCTTGTTTTGAAGAGGAAAGAG
AAGCGTCTCGCAGTAGTTAAGACATCAAATGCAATCACTCCTATCGTATGGGATGACAT
TCCCCTCATATGCTTGGATATGTGGGAGCATGCTTATTACTTGGATTACAAGAATGATAG
AGCAGAGTATGTAAATGCTTTTATGAACCACCTTGTATCATGGCATGCAGCAACAGCAC
GCATAGCTCGAGCGCAGGCCTTTGTCAATCTAGGAGAACCAAAAATTCCGGTTGCCTG
A
>GpCSD4
ATGAGATTTATGAGGGCAATGTACACCACAACAACCATCGCCATAGCAACAGCATCAG
CTCTCCCTGCTGTTTTTCTCTCCTCATCTTGTTCTTCGCCGGATTCGAAACCGCGTAACT
TGCGATTTCTAACTCCAAATACAATCTTAGTTAAAAATATGAGCACTTCTCCTTCAGCTG
TTCAGATGAAGTCTCCCTTGCAGGATAGTATCTCGTTGCCGGAATTAGTGACGGAGTTT
ATGGTGGACATGAAGTGCGAAAGCTGTGTCGATGCTGTCAGGAGTAAATTAGAGACTG
TGGGAGGAGTGAAGAATGTTGAAGTGGATTTGAGCAATCAAGTTGTTAGGATTCGCGG
TTCTTTACCTGTAAAAAGCATGGAAGAGGCTTTGGCACAAACAGGTCGTAAAGCTAAA
TTAATTGGTCAAGGTGTACCTGAAGATTTCCTTATATCCGCTGCTGTTGCTGAATTCAAA
GGCCCTGATATCTTTGGAGTTGTCCGCTTTGCCCAGGCAAACATGGAATTGGCTAGAAT
TGAGGCCAATTTTAGTGGATTATCACCTGGTAAACATGGTTTGTCTATAAACGAGTTTG
GTGATCTGACTAGAGGTCCAGCCAGCACGGGGAAAATGTACAACCCAACAATCCAAG
GAAGTGAAACAGAGCCATTGGGCGACCTGGGAACATTGGAGGCTGATGAGAGAGGTG
AAGCATACTATTCAGGCATTAAGAAGAAGCTTAGGATAGTAGATTTGATTGGACGGTCC
GTTGTGTTATATGAAACTGAGGACAAGTCAGATCCTGGCCTCGCAGCTGCAGTGATTG
CTAGAAGCGCTGGAGTAGGGGAGAACTACAAAAAGCTGTGCACTTGTGATGGAACCA
CAATTTGGGAGGCCAAACCCGATTTTGTCAGTAGCAGAGTCTAA
>GpCSD3
ATGGGCACACCACTTAAAGCAGTTGCTGTTATCTCTGGCTCTCCTAATGTCAAAGGCTC
TCTTCACTTTATTCAAGACTCTTCTGGAGTAACCCATGTGAAAGGAAGAATTATAGGGC
TAAGCCCTGGACTCCATGGTTTCCATATTCATGCCCTTGGTGATACCACTAATGGCTGCA
ACTCTACTGGGCCACATTTTAATCCAATGAAGAAGAATCATGGAGCTCCTTATGACAAG
GAAAGGCATGCTGGTGATCTTGGTAACGTTGTTGCTGGAAATGATGGTATCGCTGAGGT
CTCTATTAGAGATGCCCAGATACCTCTTACTGGGCAGCATTCAATACTTGGAAGGGCTG
TTGTGGTGCATGCTGACCCAGATGATCTTGGAAAAGGAGGACATGAACTCAGCTTGAC
AACAGGAAATGCTGGCGCGAGAGTAGGATGTGGCGTCATCGGACTACAGTCATCTGTT
TAA
>GpCSD1

ATGGGAAAGGCGGTCGCCGTTTTGAGCAGCAGTGAGGGTGTCAAGGGAACTGTTTTC
TTTGCCCAAGAAGGAGATGGTCCAACAACTGTGACTGGAACTATCTCGGGCCTCAAGC
CCGGGCTCCATGGATTCCATGTCCATGCCCTTGGTGACACAACAAATGGTTGCATGTCA
ACTGGGCCTCACTTCAATCCTGCTGGAAAAGAGCATGGTGCTCCAGAAGATGAGGTTC
GTCATGCTGGTGATCTTGGGAACATTACAGCTGGGGATGATGGTACGGCTACCTTCTCG
ATCATTGACAGTCAGATTCCTCTTTGTGGTGCAAACTCTATTGTGGGTAGGGCTGTTGT
TGTCCATGCTGATCCTGATGATCTTGGAAGAGGGGGGCACGAGCTCAGCAAGACCACA
GGCAATGCTGGTGGAAGAGTAGCGTGCGGTATTATTGGTCTTCAAGGCTAA
>GpFSD1
ATGTCTACAGTTGTTGCAACTTCTCCAGTTTCACTGTCTTCTTCCTTTCTTTCTCATGAT
GTTTATAAGAGATTCAAGTCTACTCCTTTCTTACCTTGGGGTTCTGAAAAGAGGCGAGT
AGGAAGAGCTGGTGCTGCAGTTGTTGCCAAGTTCGACCTGAAGCCTCCTCCATATCCA
TTTGATGCACTGGAGCCTCACATGAGCAAACAGACATTTGAGTTTCATTGGGGGAAGC
ATCATAGAGCCTATGTGGATAACTTGAACAAGCAGATAGTTGGAACAGAATTAGATGCG
ATGTCGCTGGAAGATGTAGTACGTGTTACATATAACAAAGGCGATGTACTTCCATCCTTC
AACAATGCAGCTCAGGCATGGAATCATGAATTCTTTTGGGAGTCCATGAAGCCTGCTG
GTGGGGGAAAGCCGTCAGGGGAGCTTTTGGCTCAAATTGAGAAAGATTTTGGTTCTTT
TGAAGCATTTGTTAATGAATTTAAGTCTGCTGCGGCCACACAGTTTGGTTCTGGCTGGG
CTTGGCTTGTGTACAAAGCAAACGAGGTAGATGTTGGAAATGCTGAGAATCCTAAACC
ATCTGAGGATGACAAAAAGCTTGTCATTGTTAAGAGCCCCAATGCCGTGAACCCTCTT
GTGTTCGATTATTACCCTCTTCTAACTATTGATGTGTGGGAGCATGCATATTACTTGGACT
ATCAGAATCGAAGGCCTGATTATATTTCCATATTCATGGACAACCTTGTTTCCTGGGATG
CAGTCGGTGCAAGATACGAGACAGCTAAGGCCCTTGCTGCATAG
>GpMSD2
ATGGCGCTAAGAACATTGATGACTACCAGGAAAACCCTAGCTTTAACTCCTCTACAATG
GCGGTCTTTACAAACCTTCACTCTCCCCGACCTTCCTTACGACTATGGCGCACTCGAGC
CTGCTATCAGCGGCGAAATCATGCAGCTTCACCACCAAAAACATCACCAGACTTACGT
CACTAACTACAACAAAGCTCTCGAGCAGCTCCACAATGCGACCGAGAAAGGCGATGC
GAGCTCTGTTGTTAAGCTCCAAAGCGCCATCAAGTTTAATGGCGGAGGTCATATTAATC
ACTCAATTTTCTGGCAAAACCTTGCTCCAATTAAGGAAGGAGGTGGCGAGCCGCCAAA
AAGCTCACTAGGATGGGCTATTGATTCAAACTTCGGCTCTCTAGAAGCTCTGATCCAAA
AAATGAATGCAGAAGGAGCTGCCTTGCAAGGTTCAGGTTGGGTGTGGCTAGGACTTG
ACAAGGAGTTGAAGACACTTGTGGTTGAAACCACTGCAAACCAGGACCCGTTGGTGA
CTAAAGGCCCAAGTTTAGTTCCTTTGGTTGGTATAGATGTTTGGGAGCACGCATACTAC
TTACAGTACAAGAATGTCAGGCCTGACTATCTTAAGAACATTTGGAAGGTGATGAACT
GGAAATATGCTAGCGAGGTGTACGAAAAAGAATGCCCTAGCCACTAA
>GpMSD1
ATGGCGTTACGAACATTGTTGACCAGAAAAACCCTACCTTCATCATCATCAGCAGCACT
CCAATGGCGGTCGTTGCAGACCTTCACTCTCCCCGACCTTCCTTACGACTATGGCGCAC
TCGAGCCTGCTATCAGCGGCGAAATCATGCAGATTCATCACCAGAAACATCACCAGAC
TTACGTCACCAACTATAACAAGGCTCTTGAGCAGCTTCATGATGCGTCTGAGAAGGGT
GATGCGACGACTGTTGTTAAGCTCCAGAGCGCCATCAAGTTCAATGGCGGAGGTCATA
TTAATCACTCAATTTTCTGGCAAAACCTTGCTCCAATTAAGGAAGGAGGTGGCGAGCC
GCCAAAAAGCTCACTAGGATGGGCTATTGATTCAAACTTCGGCTCTCTAGAAGCTCTG

ATCCAAAAAATGAATGCAGAAGGAGCTGCCTTGCAAGGTTCAGGTTGGGTGTGGCTAG
GACTTGACAAGGAGTTGAAGAAGCTTGTGGTTGAAACCACTTCAAATCAGGACCCGT
TGGTGACTAAAGGCCCAAGTTTAGTTCCTTTGGTTGGTATAGATGTTTGGGAGCACGCA
TACTACTTACAGTACAAGAATGTCAGGCCTGACTATCTTAAGAACATTTGGAAGGTGAT
GAACTGGAAATATGCTAGCGAGGTGTATGAAAAAGAGTGCCCTAGCCACTAA
>GpAPX6
ATGAGGACCACTTTCTGGGAAACCTCTTCCTTCACTTCCCATTCATCCTCTTTCCTTATT
CTTTCCAAACTTCCCTACACATTTTCACATCCAACTGTCCATTTTCGTGCCAAATTAACT
CCCTCCTTTTCTACTCCACCTTCTTATGATAACTATGCCAATTCTGCATCATGTGGAAGG
AGAGGCCTCTTTTTCCTTTCCGTCACTATGCCGTTTCTTTCCCTCCTGCCAGATTATGTG
AAATACTTTCAAGCTGAAGCCAAAGAGTATGGAACTTCTGAGTACTCTCTAATACAGG
ATGAGATCAGGAAGGTTTTGTCCAAAGGAAAGGCTGCTGGTGTACTTCGTCTTGTCTT
CCATGATGCAGGAACATTTGAAAGCAGTGATAACTCAGGTGGTATGAATGGCTCTGTAG
TATATGAACTTGACAGACCTGAGAATGCAGGGCTCAAAAAATCTCTGAAGATTCTTGA
GAAAGCAAAGAATGTGCTGGATGTGAAACAATCAGTGTCATGGGCAGACCTGATTGCT
GTGGCTGGAGCTGAGGCAGTCTTATTATGTGGGGGTCCAGTGATCCCCATTCGAGTTGG
GAGACTTGATTCACTAATTCCTGATCCCGAAGGGAAACTACCTGAGGAATCACTGGAT
GCACCACGACTGAAGCAATGTTTTCAAAAGAAAGGCTTCTCGACACAAGAACTTGTT
GCACTGTCTGGAGCCCATACTCTTGGTAGCAAAGGTTTCGGAAACCCTATTGTATTTGA
CAATTCATATTACAAAATACTCCTTGAGAAGCCATGGAGGTCATCGGCTGAAATGACTA
GTATGATTGGACTTCCTTCAGATCGTGCACTAGTCGAAGATGATGAATGTTTAAGATGG
ATTACAGAGTACGCCAATGACCAAGATATGTTTTTCGAGGATTTTAAGAATGCCTACATC
AAGCTAGTCAATTCTGGTGTTAGTTGGAGAGCTTCATGA
>GpAPX1
ATGGGAAAGAGTTACCCAACTGTTAGTGAGGACTACCAGAAGGCTATCGAGAAGGCC
AAGAAGAAGCTCAGAGGTCTTATCACTGAGAAGCAATGCGCTCCCCTTATGCTTCGTC
TGGCATGGCACTCTGCTGGTACTTTTGACTGTCAATCAAAGACTGGAGGACCTTTCGG
AACTATGAGGTTCGCTGCAGAGCAGGCTCACGGTGCTAACAATGGTATTGACATTGCC
ATCAGGTTGCTCAATCCCATCAAGGAGCAGTTCCCCACCATTTCTTACGCTGACTTTTAT
CAGTTGGCTGGAGTTGTTGCTGTCGAAGTTACTGGTGGACCTGAAGTTCCCTTCCACC
CTGGCAGAGAGGACAAGACCGAGCCACCACAGGAAGGACGTCTCCCTGATGCCACCA
AGGGTTGTGACCACTTGAGGGATGTCTTCATTAAGCAAATGGGTCTTACTGACCAGGA
CATTGTTGCTCTCTCTGGAGGCCACACTCTGGGAAGGTGCCACAAGGAGCGCTCTGGC
TTTGAGGGAGCTTGGACCTCCAACCCTCTGGTCTTTGACAACTCTTACTTCAAGGAGC
TTCTGACTGGTGAGAAGGAAGGTCTCTTGCAGTTGCCGTCTGACAAGGCTCTTCTGTC
TGACCCTGCCTTCCGCCCATTTGTTGAGAAATATGCAGCGGATGAGGATGCATTCTTTG
CCGACTATGCTGAGGCTCATATGAAGCTTTCTGAGCTTGGGTTTGCTGATGCTTAA
>GpAPX7
ATGAGCGAGAGCCTAAGAATGACAACACCCTTAGCTATCACTGCATCCTCCCGCCTTGT
GCCCTCCGCCGCCTACTCTTCTGTTTCCCGACTATCTCTCCCTTCTTCTTCACTGAAGGT
GAAATGGGGGAGATCAACGGCGCCTCTACTATCAAACCTCCCTTTGCTTAGGAAGACA
AGAGGAGCTAATCTTCACCGGAGTTTCACCACCAAATGTTACGCCTCTGATCCTCAGC
AGCTCCAGAGTGCCCGTCTTGATATCAAGCGCCTCCTCTCCTCCCACTTTTGCCATCCT
ATCTTGGTTCGGTTGGGGTGGCACGATGCTGGTACTTATAACAAGGACATCCCTGATTG

GCCTCAAAGAGGGGGTGCCAATGGTAGTCTCCGCTTCGAGGTCGAGCTCCAACACGG
CGCCAATGCTGGTCTTGTGAATGCCTTGAAGCTACTAGCGCCCATCAAAGACAAGTAC
TCTGGAGTGACATATGCTGATCTGTTCCAGTTGGCTAGTGCTACTGCTATTGAGGAGGC
TGGTGGTCCTAAGATACCCATGAAGTATGGAAGAGTGGATGTTACAGGGCCTGAGCAG
TGTCCTGAAGAAGGAAGGCTTCCTGATGCTGGACCTGCATCACCTGCTCAACATCTGC
GCGATGTTTTCTATAGAATGGGTCTCAATGATAAGGAAATAGTTGCATTATCTGGAGCAC
ATACATTGGGAAGGTCTAGACCTGAACGCAGTGGTTGGGGCAAGCCAGAAACTAAATA
TACGAAAGACGGACCAGGAGCTCCTGGTGGACAGTCATGGACAGTGCAGTGGTTGAA
GTTTGATAATTCCTATTTCAAGGACATCAAAGAAAGGAAAGATGAAGATTTACTCGTTT
TGCCAACTGATGCTGTTCTCTTTGATGATCCATCTTTCAAGTTGTATGCTGAGAAATATG
CTGCTGATCCTGAAGCTTTTTTTTCGGACTATGCTGAAGCACATGCCAAGCTTAGCAAC
CTTGGAGCGAAGTTCGACCCACCTGAGGGTTTTGCCCTAGATGAAGCTTCTGCAGGAG
CAGGTCCGGAGAAGTTTGTAGCAGCCAAGTACTCATCTGGCAAGAAAGAGTTGTCAG
ATTCAATGAGGCAGAAGATTCGTGCAGAATATGAAGCATTGGGTGGCAGCCCTGATAA
GCCTCTCCCGACAAACTATTTCCTAAACATTATGATTATTATAGCTGCTTTGGCAGTTTTA
ACCTATCTTACAGGAAACTGA
>GpAPX3
ATGGCGATGCCGGTAGTAGACACGGAATACCTTAAACAAATAGACAAAGCTCGCCGCG
ATCTCCGTTCTCTTATTTCCTCTCGCAACTGCGCTCCTATCATGCTCCGTCTCGCGTGGC
ACGACGCAGGCACGTATTGCGCGAAGACGAAGACAGGTGGGCCCAACGGTTCCATCA
GGAACGAGGAAGAGTGTGCTCATGGTGCTAATAATGGTCTCAAGAAGGCCATTGATTG
GTGTGAGGAAGTTAAAGCTAAGTATCCAAAGATCACATATGCAGACTTGTATCAGCTTG
CTGGTGTTGTTGCGGTTGAGGTGACCGGAGGGCCAACCGTTGACTTTGTTCCTGGTAG
AAAGGACTCCATGGTATCTCCCAACGAAGGGCGGCTTCCAGATGCCAAACAAGGTGC
ACCACATCTGAGGGACATATTCTATCGGATGGGTCTCACTGACAAAGACATTGTAGCCC
TTTCGGGAGGTCACACCCTGGGGAGGGCTCATCCAGAGAGATCAGGTTTTGATGGCCC
TTGGACCCAAGAGCCCCTTAAGTTTGATAACTCCTATTTTGTTGAACTGCTGAAAGGGG
AATCAGAGGGGTTGCTGCAGCTTCCTACTGACAAAACTCTGGTGGAAGATCCAGCATT
TCGCCCTTATGTTGAGCTCTATGCTAAGGATGAGGATGCGTTCTTCCTCGACTACGCTG
CTTCACACAAGAAACTCTCTGAACTAGGCTTTACTCCTGGTGGGTCAAAGTCAGGTCC
CAAGGACAGCATTGTATTGGCTCAAAGCGCGGTTGGGGTTGCAGTGGCCGCAGCTGTG
GTCATCCTGGGTTACATCTATGAAGTACGCAAAAGAGTGAAGTGA
>GpAPX10a
ATGGCACAAATGGTAAGAAGAGAAGGGCAAATAGTAGATGCAGAATACCTGAAACAA
ATTGAGGAAGCTCATAGAGACCTTCACAATCTACTCTCCCACAACAAGCAGTTTGCTC
CTATTGTGCTTCGAATGGCATTTCATGATGCAGCAACTTATGATGTTAGGACTAACAATG
GAGGTCCAAATGGTTCAATAAGGAATGAGTTGAACAATCCTGCTAACAAAGGGATTGA
GAAGGGAGTTGTTTTTGTTGAACCAGTGAAGAAGAGACACCCTAAGATAACTTATGCT
GATCTTTATCAGCTTGCTGGAATAGTTGCAATTGAGGTCACAGGAGGTCCTAAGATCCC
TTTCACCCCAGGTCGAAAGGATGTTTTGCAACAGCAAGACAAAGGAAGCGTTCCCAA
TCCCAATGGAGGTGCTCAACATTTGAGAGATGTATTTCATCGTATGGGTCTGTCTGACA
TGGATATTGTGGTGCTATCAGGGGCACATGCACTGGGAAGGGCACATAAGGATAGATC
AGGATTTGATGGTCCTTTTACAAGGGACCCTTTGAAATTTGACAATTCCTACTTTGTGG
AGCTTATGAAGGGTGACAGCCCAGGGCTGGTGAAGTTTCCTACTGATAAATCTCTCTTG

AAAGACCCTGTCTTCCGTCCCCTTGTCGAACACTATGCTAAGGATCAGAAAGCATTCTT
CAGAGACTATGCTGCATCACACAAGAAGCTATCAGAGCTTGGGTTCCACCCTCTTGAC
ACTCATTCCCACCCCCACCCACAAACCCATGCACACTCGCACTCACACTCACACCAAC
ACCAGCACCTAACTAGCCACCAGCGCCATGCGCATCCACACTCTCACCACTGCTCACA
GCCAATGCCACCCTGCCATGGATTTGCTTGA

>GpAPX10

ATGGCCATGAAAGTTCCAGTGGTAGATGCTGATTATTTGAAGGAAATTGAGGCTGCTCG
CAATGATCTTCGGTCCCTTATCGCTAACCAGCGTTGCGCTCCTATCATGCTTCGCTTAGC
ATTTCACGATGCAGGAACTTATGACGCGAAAACAAAAACAGGGGGTCCAAATGGTTCA
GTAAGATTTGAGCTAAACAGCCCTGCCAACAATGGCATCAAAGTTGCTGTTGATCTTTG
CGAACAAGTGAAGGCTAAACATCCCAACATTACTTATGCTGACCTCTATCAGCTTGCTG
GAGTTGTTGCAGTTGAAGTTACCGGAGGGCCTGCTATCAATTATGTTCCAGGTCGACCT
GATGTCCTGCAAGCACAAGACAATACCGGAAATCTTCCAGTACCTACTGGAGATGCTC
AGCATTTGAGAGATGTGTTCCACAGAATGGGTCTTAATGACAGGGATATTGTTGCACTA
TCAGGATCACATACTCTGGGAGGAGCGAAGCGGGATAGATCAGGAGTTGAGGGTAATT
TTACCAAGGAGCCTCTGAAGTTTGACAATTCCTATTATGTAGAGCTGAGGAATGCTAAT
CCCCCGCCGGAGCTTGTGAAATTTAACACAGATAAGGTTCTTAAGGAGGATCCTGTATT
CCGTAAATATGTTCAGCTTTATGCTAAGGAGCAACATACATTCTTCACACATTATGCAGA
ATCACACAAGAAGATGTCAGAGCTTGGACGCCCTGTTCACGCTTGA

>GpCAT1

ATGGATCCTTACAAGTTTCGACCGTCAAGTGCTTACAATTCGACATACTTCACAACGAA
TTCGGGTGCTCCTGTTTACAATAACAACTCTTCCTTGACTGTTGGTACCCGCGGTCCCA
TTCTTTTGGAGGACTACCATTTGGTGGAAAAGCTTGCCAACTTTGACAGGGAGCGTAT
CCCAGAACGTGTAGTTCATGCTAGGGGAGCTAGTGCTAAGGGGTTCTTTGAAGTGACC
CACGATATTTCTCACCTTTCATGTGCTGATTTCCTGCGTGCTCCCGGTGTTCAGACACCT
GTCATTGTTCGTTTCTCCACTGTTATTCATGAACGTGGAAGTCCTGAAACACTTAGGGA
TCCTCGTGGTTTTGCTGTAAAGTTTTATACCAGAGAGGGTAACTTTGATCTGGTAGGGA
ACAACTTCCCTGTGTTTTTCGTCCGTGATGGTATGAAGTTTCCTGATATGGTGCATGCTT
TGAAACCCAACCCCAAAAATCACATCCAAGAGAACTGGAGGATCATGGATTTCTTCTC
TCACCATCCAGAGAGCTTGCACATGTTTACTTTTCTCTTTGATGATATTGGTGTTCCAGC
TGACTACAGGCACATGGAAGGGTCTGGTGTTAACACATACATGTTGGTTAACAAGGCT
GGCAAAGCTTATTATGTGAAGTTCCACTGGAAGCCAACTTGTGGCGTGAAGTCCTTGT
TGGAGGATGAGGCCATAAAAGTTGGGGGAGCTAACCACAGCCATGCAACCCAGGACC
TTCATGACTCAATTATGGCTGGGAACTACCCTGAATGGAAACTGTTCATCCAAACAATA
GACCCAGCAGATGAAGATAAATTTGACTTTGACCCACTAGATGTTACCAAGACATGGC
CAGAGGACATTTTGCCCCTACAGCCAGTTGGACGCTTAGTCTTGAACAAGAACATTGA
CAACTTCTTTGCTGAAAATGAGCAGCTCGCATTTTGCCCTGGTATTGTTGTTCCTGGTG
TTTACTACTCAGATGACAAACTGCTTCAAACTCGAATCTTCTCGTACTCTGATACCCAG
AGACACCGTCTTGGTCCCAACTATCTGCAACTTCCAGTTAATGCCCCCAAGTGTGCTCA
TCACAACAATCATCATGATGGCTTCATGAACTTCATGCACAGGGATGAGGAGGTCAAC
TACTTCCCCTCAAGATATGATCCTGTTCGTCACGCTGAGAAATATCCCATTCCATCTAAT
GTATTGAATGGGAAACGTGACAAGCAAGTCATAGTGAAGGAAAACAACTTCAAGCAA
CCTGGAGAGAGATACAGATCATTTGACCCAGCCAGGCAAGAGAGATTCATCCAAAGAG
TGATTGATGCTCTATCTGATCCTCGTGTCACACACGAGGTTCGTGCTATTTGGGTTTCAT

ACTGGACACAGGCTGACAAATCTTTGGGCATGAAACTAGCAAGTAGACTTAATGTGAG
ACCAACCATGTGA
>GpCAT2
ATGGATCCCTACAAGTACCGACCGTCAAGTGCTTACAACACCCCCTCCTTGACCACTAA
GCCTGGTGAACCAGTATACAACGACAATTCATCATTGACAGTTGGTCCTAGAGGTCCA
AGTCTGTGGGAGGATTACCTTCTGATAGAGAAGATCACCACCTGGACTCATGAGCGTAT
ACCAGAGCGTGTTGTTCATGACAGGGGAGCCAGTGCTAAGGGTTTCTTTGAAGTCACC
CATGAAATTACTCATCTCACTTGTGCTGACTTCCTCCGTGCTCCTGGAGTTCAGACTCC
TGTCATCGTTCGTTTCTCCACTGTTATTCATGAGCGTGGTAGCCCTGAAACCCTCAGAG
ACCCTCGTGGTTTCGCTACCAACTTCTACACCCGAGCGGGTAACTTTGACTTGGTGGG
ACCCAAGTTCCCAGTATTCTTCACTCGGGATGCAATGCAATTATTTGATCTTATACGTGC
ATTCAAACCCAACCCGAAATCTCAAATCCAAGAGGACTTGAGGTTTCTAGACCACTGC
TCCTACCTCCCGGAGAGTATGACCACATTCACATTCTTCTATGATGATATGGGTATTCCT
CAGGACTACAGACACATGATAGGTTCTGGTGTCCACACTTTTACTATGCTTAACAAGGC
AGGCAAGGTCACCTATGTCAAGTTCCACTGGCGGCCAACATGTGGTATCAAGAACATG
TTGGAAGAAGAGGCCGCTAAAGTTGTAGCAGGCAACCACAGCCATGCTACGAAGGAT
CTGTGGGACTCAATTGCATCAGGAAACTCCCCTGAATGGAAGCTCTTCATCCAAACAA
TGTATCCAGCTGATGAAGACAAGTTTGATTTTGACCCGCTTGATACTACAAAGACATGG
ACAGAGGACATTCTGCCGTTGCAGCCTGTGGGGCGTATGGTCTTGAACAAGCACATCG
ACAACTTCTTAATGTAG

CDS sequence of SOD, APX and CAT gene family members in *Heliosperma pusillum*

>HpCSD1
ATGGTGAAGGCTGTGGTCGTTTTGAGCAGCAATGAGGGTGTTAGCGGAACTGTCC
ACTTCACTCAAGAAGGAGATGGTCCAACAACCGTAACTGGACACATGACGGGTC
TCAAGCCTGGGCTCCACGGATTCCATGTTCATGCCCTTGGTGACACCACAAACGG
TTGCATGTCAACTGGACCACACTTTAACCCAGCTGGGAAAGAGCACGGTGCTCCA
GAAGATGATGTCCGCCATGCTGGTGACCTTGGAAACATCACAGTTGGAGATGATG
GTAGTGCAACCTTTACAATCATTGACAATCAGATTCCTCTTTGTGGACCCAACTCT
ATTGTGGGAAGAGCTGTTGTTGTTCATGCTGATCCGGACGATCTTGGAAGAGGGG
GTCATGAGCTCAGCAAGAGTACCGGTAATGCTGGTGGAAGAATTGCTTGCGGTGT
AATTGGTCTTCAAGGCAATATCTGA
>HpCSD1a
ATGGGAAAGGCTGTCGTCGTCTTGAACAGCAGTGAGGGTGTCAAAGGAACTGTC
CACTTCAATCAAGAAGGAGATGGTCCAACAATCGTGACTGGAACTATCTCGGGTC
TCAAGCCTGGTCTCCATGGATTCCATGTTCATGCTCTTGGTGACACTACAAACGGT
TGCATGTCTACTGGACCACACTATAACCCTGCTGGGAAAGAGCACGGTGCTCCAG
AAGATGAAGTTCGCCATGCTGGTGACCTTGGAAACATCACAGCTGGGGATGATG
GTACCGCAACCTTCACAATCACTGACTGTCAGATTCCTGTTTCCGGACCACACTCT
ATTGTGGGAAGGGCTGTTGTTGTCCATGCTGATCCTGATGACCTTGGAAGGGGGG
GTCATGAGCTCAGCAAGACCACCGGTAATGCGGGTGGAAGAATTGCTTGCGGTA
AGCTGCTCTTAATCTGCATCTGTTTTTGCTACGTCATTGATCTGTAA
>HpCSD4
ATGAGTTTGCCGGAATTAGTGACGGAGTTCGTGGTGGATATCAAAAGTGAAGAA
TGTCTAACTTGTATCACCAATAAACTAGAGACGGTCCAAGGAGTGAAGAGTATTG

ACGCGGACTTGAGCAATCAAGTCGTTAGAATTCGCGGTTATTCCCCTCTCAAAAC
CATGGAAGAGGCTTTAATTCAAACAGGTCGTACTGCTCGCCTTATTGGTCAAGGA
ATGCCTGATGATTTTCTCGTATCTGCGGCTGTGGCTGAATTCAAAGGCCCTGATAT
ATTTGGAGTTGTTCGTTTAGCCCAGGCAAATATGGAATTAGCTAGAATTGAGGCC
AATTTTAGTGGGTTGCCACCTGGAAAGCATGGTTTCTCAATAAATGAATACGGTG
ATCTGACAAGAGGTCCAGCCAGTACTGGGAAAATGTACAGTCCGCCAATCCAAG
GCAGTGAAACTGAGGTATTCTCCCCTTGTTCAAATAAGATTGATCACCATTTCATT
TAG
>HpFSD1
ATGTCTGCTGTTGCTGCGACTTGTTTCTCATCCTTGTCTTCAACATTTGTTTCTCAT
CATCCAGGCTCTAATTGGGGATCTAAGTCTATCCCACTCACACCTTTGAGATTTAA
GCAAACACATGTAAGAAGAGCTGGAGCTGCTGTAGTAGCCAAGCTTGAGTTGCA
GCCTCCGCCATATCCATTTGATGCCTTGGAGCCTCACATGAGTAAACAGACTTTT
GAGTTTCATTGGGGGAAGCATCATAGATCATATGTTGATAACCTGAACAAACAAA
TAGTTGGAACAGAGTTAGATGGAAAGTCACTTGAAGATATAGTGAGTATAACAT
ATAACAAAGGTGAAATGCTTCCATCTTTCAATAATGCAGCACAGGCCTGGAATCA
CCAGTTCTTTTGGGAGTCGATGAAACCTGGTGGCGGGGGGAAGCCATCTGGAGA
ACTTTTGGCTCAAATTGAGAAAGATTTTGGTTCTTTTGAAGCATTTGTTACTGAAT
TCAAGTCTGCTGCTGCCACACAATTTGGTTCTGGTTGGGCTTGGCTTGCTTACAAA
ACGATGAAGCTGGATGTGGGTAATGCGATAAACCCTAGACCTTCTGAAGATGAC
AAAAAGCTTGTGATTGTTAAGACACCCAATGCTGTAAACCCTCTTGTTTGGGGTT
ATAATCCTCTTCTGACCATTGATGTTTGGGAGCATGCATATTACTTGGACTACCAG
AATCGAAGGCCGGATTACATCTCAATATTCATGGAGAATCTTGTTTCCTGGGATG
CCGTCAGTGCAAGATATGAGGCCGCCAAGGCACTTGCTGCTTAA
>HpFSD2
ATGGGAAGTTGTACTACTACTTTTCATACTGTCTCTTCTCTCCATTGTATTGATTTT
TCTTCCACTAGTAAAAATCCAAGGTTAATTCCTCAGAAGCGTATAGGATGCCTTG
GACGAAGAAGAGGACCTAAAGTAGTTGCGTATTATGGGCTAAAGAAGCCTCCTT
ATAACCTAGATGCTTTGGAACCATATATGAGCAGAAAGGCACTCGAGTTACATTG
GGGAGAGCATCATAAAGGCTATGTGGATGCTTTAAACAGACACCTAAGTAAGAG
TGACATACTATATGGCTATACCATGGATGACCTTGTTAAAGTGACATACAACTAC
GGAAACCCCTCACCGGAATTCAATGATGCTGCACAGGTTTGGAACCACGAATTTT
TCTGGGACTCCATGCAACCCGGAGGCGAGGATTCACCAAAGCTTGGACTGCTTCG
GCATATTGAGGAAGATTTTGGTTCTTTCACAAATTTTAAAGAGAAATTTACCGAG
GCAGCCCTTACATTATTTGGTTCTGGCTGGGTTTGGCTTGTTTTAAAGAAGGATGA
AAAGCGTCTAGTGATAGTTAAAACATCAAATGCAATCACTCCCATCGTATGGGAT
GATATACATGCTTATTATTTGGATTACAAGAACGATAGAGCCAAGTATGTAAATA
TATTCATAAACCACCTTGTATCGTGGCATGCAGCCACTGCACGAATAGCTCGAGC
TCAGTCTTTTGTTAACCTAGGCGAACCTAATATTCCCATCGCTTGA
>HpMSD1
ATGGATGTTAACATAATGTTATTGCAGGAAGGAGGTGGCGAGCCTCCAAAAAGC
GCTTTGGGTTGGGCTATTGATTCAAATTTTGGCTCTCTTGAAGCTCTTATTCAGAA
AATGAATGCCGAAGGAGCTGCCTTACAAGGTTCTGGTTGGGTGGATCCACTGGTA
ACTAAAGGGGCAACTTTGGTTCCTTTACTTGGTATAGATGTCTGGGAGCATGCTT

ACTACTTGCAGTACAAGAATGTGAGACCTGATTATCTGAAGAACATATGGAAGGT
TATGAACTGGAAATATGCCTGTGATGTTTATGAGAAAGAGTGTCCTAGCACTTAA
>HpAPX6
ATGATGACCACCTTCAACAACCCACCATTTTTAAGCTGCTGCTTCACTTCCCTATCTTCT
CCATTCATCACCAACTTCATTTTTACCGCCAAATTTCAACCTCAAACTGTTAATTTTGGC
GCCAAATCAGCTGCATTACTCAAACCCACTTGCGCAATTCAACCATCTTCTGGTGGCTG
TGGGAACCTTGAATCATGTGGAAGAAGAAGACTTCTTTTGCTTTCTGCAACAGTGTCT
TTTCTTATTCATATGCAGGAATACACCAAAGATTTCTATGTTCAAGCGGAAGAGCTGGA
AACTTATGGGGACCAAATTGTAAGAGATGAAGTAAGGAAGGTTTTGACTAAAGGGAA
GGCTGCTGGTATTCTTCGTCTTGTTTTTCATGATGCAGGAACCTTTGAAAGCAACAACA
ATTCAGGTGGTATGAACGGCTCTATAGCATATGAACTTGAAAGACCAGAGAATAAAGG
GCTAAAGAAATCTTTGAAGGCATGCTTTCACACTTCTTACTCCCGACCCCTTTCTCCTC
ATATTATCATTGATGATAGCATTCTTGAGAAAGCCAAGAAAGAGTTGGATATGCGAGAA
TCAGTGTCATGGGCTGACCTGATTGCTGTAGCTGGAGCTGAAGCAGTCTCAATATGTGG
AGGTCCGGTGATTCCTATTCGGGTGGGAAGACTTGATTCTTTGACTCCTGATCCTGAAG
GGAAGCTGCCCGAGGAATCACTAGATGCGCCAGGACTGAAAGAGTGCTTTAAAAGAA
AAGGCTTCTCGACTCAAGAACTTGTGGCGCTGTCTGGGGCCCATACAATTGGTAGTAA
AGGTTTTGGAAGCCCCATTGAATTTGATAATTCATACTACAAAATCCTACTTGATAGGCC
TTGGGCATCAGCAGGTGGAATGTCAGGTATGGTTGGACTTCCTTCAGATCGTGCACTG
GTCGAGGATGAAGAATGCTTAAGATGGATTAAAAAATACGCGAAGGATCAAAATTTGT
TTTTCGATGATTTTAAGGATGCTTACATCAAGTTGGTCGATTCGGGCGCAAGGTGGAAA
TCTTCATGA
>HpAPX7
ATGGAGGATTTTTCTTTTAGAGTTTTTTCCCAAGGAAGGGAACTCGACATCGAACTTTG
GATCTGGTTGCACGAAGCCGCCGTACGGTTAGGATGGCATGATTCAGGTACTTATAACA
AGGATATCCAAGATTGGCCTCAACGAGGTGGAGCTAATGGCAGTCTCCGCTTCGACGT
TGAACTTAAACATGGAGCTAATGCTGGTCTTGTAAATGCCTTGAAACTTATCCAGCCCA
TTAAGAACAAGTATTCCGGAGTCACTTATGCAGATCTATTCCAGTTGGCCAGTGCCACA
GCAATCGAGGAGGCTGGTGGTCCTAAAATACCAATGAAGTATGGAAGAGTTGATGTAA
CAGCACCCGATCAGTGCCCAGAAGAAGGAAGACTTCCTGATGCTGGACCCCCTTCACC
TGCTCAACATCTACGTGACGTCTTCTACAGAATGGGTCTCAGTGACAAGGATATAGTTG
CATTATCTGGAGCACATACATTGGGAAGATCGAGACCAGAACGCAGTGGCTGGGGAAA
GCCAGAGACCAAATACACGAAAGATGGTCCAGGAGCACCTGGTGGACAGTCGTGGAC
AGCGCAATGGTTGAAGTTTGATAATTCTTACTTCAAGGACATCAAAGAAAAGAAAGAT
GAAGATTTGCTTGTCTTGCCAACTGATGCTGCTCTTTTCGATGACCCATCTTTCAAGGT
GTATGCTGAGAAATATGCAGCAGATCAGGAAGCGTTTTTCAAAGACTACGCTGAAGCA
CACGCAAAGCTCAGTAACCTTGGAGCCAAGTTCGACCCTCCTGAGGGTTTCTCGTTGG
ATGAACCTGCTCCAGAGAAGTTTGTCGCAGCAAAATACTCGTCTGGCCAGAAAGAGTT
GTCAGCTGCTATGAAGCAGAAGATTCGCGCAGAATATGAAGGCTTCGGTGGTAGCCCT
GATAGACCTCTTCCAACAAACTATTTTCTTAACATTATGATTGTTGTTGCTGTTTTAGCG
GTTTTATCTTATTTTGCTGGAAACTAA
>HpAPX2
ATGGAGAACAAGTGTTATCCAAAAGTAAGTGAGGAGTATGAGAAAGCAGTTGACAAG
TGTAAGAAGAAGCTTAGGGCTTTGATCCAACAGAAACATTGTGCCCCTATCATTCTTCG

TTTGGCATGGCACGCAGCAGGGACATATGACGTGCACTCGAAAACAGGTGGACCGTTT
GGAACGATAAAGAATCCACAAGAACTTTCTCATGAAGCTAATAACGGTCTTGATATCGC
TGTTCGACTGCTCGAGCCAATCAAAGAGCAATTCCCCATTTTAAGCTATGCTGATTTTA
TCAGCTGGCTGGTGTTGTTGCAGTAGAAGTCACTGGAGGACCTGAAATTCCTTTCCAC
CCTGGCCGACCTGACAAAGCTGAATCGCCCCCAAAAGGCCGATTACCTGATGCCAAGA
AAGGTTCTGAGCATTTGAGGGAAGTATTCCATCGCATGGGTCTATCCGATAAGGACATA
GTCGCTCTTTCGGGTGCTCACACCTTGATTGTGAGTTGTTTAACTGAAAGATTCAAGGG
ATATGACTTGGATACAATCCTCTACAGCAAAGCACAAGAGAAAAAGGATTCAAGACTG
GTTTTAGAGACAGTTTCAAGTGCTACAGCAAGGCACAAACACCCTAGACTAACCTCAA
CAGCAAGGCCAGCTTAG
>HpAPX1
ATGGCAAAGAACTACCCAACTGTGAGCGAGGACTACCAAAAGGCCATTGAGAAGGCA
AAGAAGAAGCTCAGAGGTCTTATCGCTGAGAAGAAATGTGCTCCACTCATGCTTCGTA
TTGCATGGCATTCCGCTGGTACCTTTGATTGCGCATCAAAGACCGGAGGACCTTTCGGA
ACAATGAGGTTCAAGGAAGAGCTGGGTCACGGAGCCAACAACGGGGTTGACATTGCT
GTCAGGTTGTTGGAGCCTATCAAGGAACAATTCCCCACCATCACTTACGCCGACTTTTA
TCAGTTGGCTGGTGTTGTGGCTGTTGAAATTACTGGAGGACCTGAAGTTCCCTTCCAC
CCTGGCCGAGAGGACAAGCCAGAGCCTCCACAGGAGGGTCGTCTCCCTGATGCTACA
AAGGGTTGTGACCATCTGAGAGACGTGTTCGTCAAGCAAATGGGTCTTTCTGACCAAG
ACATTGTTGCTCTCTCTGGAGGCCACACCTTGGGAAGGTGCCACAAGGAGCGTTCGGG
CTTTGAAGGAGCTTGGACCGCTAACCCTCTGATCTTTGACAACACCTACTTCACGGAG
CTTTTGAGCGGTGAGAAGGAAGGTCTCCTGCAACTACCATCTGACAAGGCTCTTCTGG
CAGACCCTGTCTTTCGTCCTCTCGTTGAGAAATACGCAGCGGATGAAGATGCATTCTTT
GCCGACTATGCTGAGGCTCACATGAAGCTGTCCGAGCTTGGGTCTGTTCATATTTGA
>HpAPX1a
ATGATGGCTAAGAACTACCCAACTGTGAGCGAGGACTACCAGAAGGCTATTGAAAAGG
CAAAGAAGAAGCTCAGGGGTCTTATCGCCGAGAAGCAATGTGCTCCGCTCATGCTTCG
TATTGCATGGCACTCTGCTGGTACCTTTGATGTGCAGTCAAAGACGGGAGGACCCTTTG
GTACTATGAGGCACAAGGATGAGTTGGCTCATGGAGCTAACAACGGGCTTGACATTGC
TGTCAGGTTGTTGGAGCCCATCAAGGAACAGTTTCCCACCATCTCTTATGCTGACTTCT
ATCAGTTGGCTGGTGTTGTGGCTGTTGAAGTTACTGGAGGACCAGAAGTTCCCTTCCA
CCCTGGTCGAGAGGATAAGCCAGAGCCCCCACAAGAAGGCCGTCTCCCTGACGCCAC
CAAGGGTTGTGACCACCTGAGAGATGTGTTTGTTAAGCAAATGGGTCTTACTGACCAA
GATATTGTTGCTCTCTCTGGAGGCCACACCTTGGGAAGGTGCCACAAGGAGCGTTCTG
GTTTTGAGGGAGCTTGGACTTCCAACCCTCTCATTTTCGACAACTCGTACTTCAAGGA
GCTTTTGAGTGGTGAGAAGGAAGGTCTCTTGCAGCTGCCATCAGACAAGGCTCTTCTG
TCAGACCCTGTCTTCCGTCCTCTGGTTGCGAAATATGCAGCGGATGAAGATGCATTCTT
TGTCGACTATGCTGTTGCTCACATGAAGCTGTCAGAACTTGGGTTTGCTGATGCTTAA
>HpAPX9
ATGACAACTCGAAAAGGGCAGGTGAACGATGCAGAATACTTGAAAGAAATCGATTGG
GCTCGTCGAGACCTTCGTGCATTAATATTTACCAAGCAATGTGCTCCTTTCATGCTGCGC
CTAGCATGGCATGATGCAGGCACATACGATGTGAAAACAAAGACTGGCGGTCCAAATG
GTTCAATTAGGTACGAGTTGAGCCATGGTGCAAATAGAGGACTCAAAGTTGCCATTGAT
TTCTGTGAAGAAATAAAGGCTAAATACCCCAAGATCACTTATGCTGACCTTTACCAGCT

TGCAGGAGTCGTCGCTGTGGAAGTCTCAGTTGGTCCAACCATCGACTTTGTTCCAGGT
CGAATGGATTGCCCGGGTTCTACTGATGAAGGCCGTCTTCCTGATGCTAATGGAGGTGG
CGAACATTTGAGAGAGATTTTTATCGCATGGGACTTTCCGACAAGGACATCGTTGCAC
TGTCTGGAGCACATACATTGGGTAGAGCACATGTAGAGAGATCAGGTTTCGAAGGCGC
TTGGACACCAGAGCCTCTCAAATTTGATAACTCCTACTTTGTTGAATTGCTGAAAGGAG
GAAAATCAGGGATGTTGAAACTACCTACAGACAAAGTTCTTGTACAAGATCCGGTGTT
CCTTCCCTATGTTGAACTTTATGCCAAGGACGAAGAAGTCTTCTTCAGAGATTATGCAG
ATTCTCACAAGAAACTGTCAGAGTTGGGATTTGCACCACCCAACTCTCGCCCAAATAT
GGTTACGAAGGTTGTGGTTGGCATCGCTGTTGCTATTACCGTTGCTGTCATCAGCTACT
TTTATGAAGTTTGTAAGCAAGCTAAGTAA
>HpAPX3a
ATGGCGATGCCAGTAGTTGACACTGAATACCTCAAACAAATCGACAAAGCTCGTCGTG
ATCTCCGTGCTCTCATCTCTAATCGCAATTGCGCTCCTCTCATGCTTCGCCTCGCGTGGC
ATGATGCAGGAACGTATGATGCGAAGACGAAGACAGGTGGACCGAACGGTTCGATTA
GGAATGAGGAAGAGTTTGCTCATGGTGCTAATAACGGTCTCAAGAAAGCTATTGATTG
GTGTGAGGAGGTGAAGGCTAAATATCCAAAGATCACGTATGCCGACTTGTATCAGCTTG
CTGGTGTTGTTGCAGTTGAAGTCACAGGAGGCCCTACAGTTGATTTTGTTCCTGGTAG
AAAGGACTCCAAGGTATCTCCCAGAGAAGGGCGACTTCCAGATGCCAAACAAGGTGC
ACCACATCTGAAGGACATATTTTATCGGATGGGTCTCAGTGATAAAGACATTGTAGCCT
TGTCAGGTGGACACACCCTGGGAAGGGCACATCCAGAGAGATCAGGTTTTGACGGCC
CATGGACCCAAGAGCCCCTCAAGTTTGACAACTCATATTTTGTAGAACTGATGAAAGG
AGAATCAGAGGGACTGTTGCAGCTTCCGACTGATAAAGCTCTGTTAGATGATCCTGCAT
TTCGCCCTTATGTTGAACTCTATGCTAAGGATGAGGAGGCTTTCTTCCGCGACTATGCT
GCTTCGCACAAGAAACTTTCTGAACTGGGGTTTACTCCTGGCGGGTCCAAGTCAGGGC
CAAAGGACAGCATTATACTGGCCCAAAGTGCGGTCGGGGTTGCAGTTGCTGCAGCCGT
GGTCATCCTGAGCTATATCTATGAAGTTCGCAAGAGAGTATAG
>HpAPX3
ATGGCGATGCCAGTAGTTGACACTGAATACCTCAAACAAATCGACAAAGCTCGTCGTG
ATCTCCGTGCTCTCATCTCTAATCGCAATTGCGCTCCTCTCATGCTTCGCCTCGCGTGGC
ATGATGCAGGAACGTATGATGCGAAGACGAAGACAGGTGGACCGAACGGTTCGATTA
GGAATGAGGAAGAGTTTGCTCATGGTGCTAATAACGGTCTCAAGAAAGCTATTGATTG
GTGTGAGGAGGTGAAAGCTAAATATCCAAAGATCACGTATGCCGACTTGTATCAGCTTG
CTGGTGTTGTTGCAGTTGAAGTCACAGGAGGCCCTACAGTTGATTTTGTTCCTGGTAG
AAAGGACTCCAAGGTATCTCCCAGAGAAGGGCGACTTCCAGATGCCAAACAAGGTGC
ACCACATCTGAAGGACATATTTTATCGGATGGGTCTCAGTGATAAAGACATTGTAGCCT
TGTCAGGTGGACACACCCTGGGAAGGGCACATCCAGAGAGATCAGGTTTTGACGGCC
CATGGACCCAAGAGCCCCTCAAGTTTGACAACTCATATTTTGTAGAACTGATGAAAGG
AGAATCAGAGGGACTGTTGCAGCTTCCGACTGATAAAGCTCTGTTAGATGATCCTGCAT
TTCGCTCTTATGTTGAACTCTATGCTAAGGATGAGGAGGCTTTCTTCCGCGACTATGCTG
CTTCGCACAAGAAACTTTCTGAACTGGGGTTTACTCCTGGCGGGTCCAAGTCAGGGCC
AAAGGACAGCATTATACTGGCCCAAAGTGCGGTCGGGGTTGCAGTTGCTGCAGCCGTG
GTCATCCTGAGCTATATCTATGAAGTTCGCAAGAGAGTATAG
>HpAPX10b
ATGGCACAAATGACAAGATGTGTGCAAATGGTGGATACTGAATATCTGAGACAGATTG

AAGAAGCTCGTACAGACCTTCACAATCTATTATCCCTTAATAAGCAATTTGCTCCTACTA
TTCTTCGCTTAGCATTTCATGATGCAGGAACGTATGATGCGAAAACAAACACCGGTGGT
ACAAACGGGTCAGTTAGGAATGAAATGAGCACCGCGCCTAACGTTGGCATGCAAGCA
GCTCTTGATTTCTGCGAGCAAGTGAAAGTGAGGCACTCCAAGGTCACTTATGCCGATC
TTTACCAGCTTGCCGGAGTTGTTGCAGTAGAGGTGACTGGTGGTCCGAAAATACACTT
CGCCCCTGGTCGGAAGGATGCCCCGCCGCAGCAAGACAAAGGAAATCTTCCGAAACC
TAACGGAGATAGCAAGCATTTGAGAGACATATTTCACAGGATGGGGCTCTCTGATAAG
GATATTGTGGTACTATCTGGGGCTCACACACTGGGTCGGGCACATCAGGATAGATCAGG
CTTTGATGGTCCTTTCACCAGAGACCCGCTCACATTCGACAATTCCTATTATGTGTTGTT
GATGAAGGGCGACACTGCAGGGCTCGTCAAATTTCCCACTGACAACTCTCTCATGCAA
GATCCTACATTCCGTGTCTATGTTCAAATTTATGCTAGGGATCAGAATCTGTTCTTCAAA
GATTATGCAGAGTCACACAAAAAGATGTCAGAGCTTGGATTTCACCCACATAAACATG
CCTCTCACCCGGACCCACACCCACACCCAACCTCACACTCGCATGAGGTACCCCACCA
CAACCAGAACCACAGCCACAACCACAACCACAACCAAAACCACCACGCTCAGCCAC
AGCAACTCCCTCAGTGCCGTCCACGCCCACCTTGTGGATACTTTTGA
>HpAPX10
ATGAGCTGCAATAGTCGTGAGGTCCGTCCTAACAATCGTGAAGACCGTGATATGCAGAT
AGCCAAACAATTGTTATATTCTCAATTTCCTACAAAATTTGTTTGGAAAAAGAAGCAAC
GACAATGGCCTCTTAGAAAAAAAGGGTTTAAGATTGGAAGGTTGCTTCACGTGCCACC
GAGTTGCGGAGAGCTTTATTTTATGAGAATTATGTTGAATCACATTAAGGGTCTCAAAA
GTTTCGAGCACATCAGGACTGTCAATCATGATATTGTTCGTCCGACCTATAGAGATGCAT
GTTACGCACTTGGCTTAATTGGCGACGACAAAGAATATATTGATGTGATCGAAGAAGCA
AGTGATTGGGGATCTGGATCTTATCTTCGACATTTGTTTTCGACTTTACTATTGTCTTGTA
CTTTGTCTATGCCAAGCAGAGTGTGGGACAAAACTTGGCGTCTATTATCTTACAACATC
CTTCATAGGCAGCGTAGAATCCTTAACAATCAAGGTCTACAACTAACAGACGAGGAGT
TACAAAATTACACATTGCTCGATATCGAAAATTTTCTACAGATAAATGGAAGTAGCCTAC
ATAGATTTGAAGGGATGCCCCTACCAGATATATCGGCAACGTCTCATCATCGAAACAAC
TTAGTAATGGATGAGTTGTCTTATGATAGACAGTCATTGCGTGAAGAACATGATAATAAC
TTAGCTATAATGACAGATGAACAGAGGTTGGTCTATAATGAAATTGTAGACGCTGTTTTA
AAGAATAAAGGAGGGGTGTTCTTTGTATATGGGTATGGCGGAACTGGGAAGACTTTCA
TTTGGCGAACTTTGTGTGCTGCTTTTAGATGTAAGGGTGGTATTGTTTTACCAGTGGCAT
CAAGTGGAATTGCAGCAACCTTGATTCCTGGTGATAGAACGGCTCATTCTAGGTTTGGA
ATACCGCTTAGTGTGACCGAAAACTCCACTTGCCCTCGCATTAAACCCGGAAGTGATTT
AACAGAACTTATGAGAATGACAAAACTCATAATATGGGACGAAGCGCCAATGACTCAT
AAACACAGCTTTGAGGCTCTGGATCGAAGCCTAAAAGATGTAATGCGTGTTGTAGACG
AGAGAAATGCAGACGAACCTTTTGGCGGAAAAGTGGTAGTCTTCGGGGGGGATTTTC
GTCAAATTCTACCAGTCGTGCCTAAAGGAATCAGAGCAGATATTGTGCATGCTTCTCTT
TGTTCATCTTATTTATGGTCTTCATGCAAGGTTCTTAAATTGACCAAAAACATGCGTTTG
CAAGTTGGAAGTTCAAGCAACAATGGCGATGACATAAAGAAATTTTCTGAGTGGATCT
TGGAAATTGGAGATGGCTTAGCTGGTGGTGATAATACTGGGGAAGTGGAGCTTCAATTT
CCGCTCGACCTACTCATTGAAGACGAAAGGGCAATCCTTGCACCAACTCACGAGATTG
TTGATGAGGTAAACGATTATGTTTTATCGCTTATTGACAAGGATGAAAGAATATATCTAA
GCTCTGATGAAGTTAGCAAAGACGACACCTCTATTGGTGAGCGTGATCTACATTCCACT
GAATTTCTTAATAGTATTAAATGCTCCGGTCTCCCTAATCATCAGTTGCGTTTGAAGATT

GGTGCTATGGTAATGCTTCTTAGGAACATTGACCAATCACGCGGGTTATGTAATGGCAC
GAGATTGATTGTGACAGCTTTGGGAACGCGTGTTATTAGGTGTACGGTTTTAACTGGTA
GTCGGAAAGGTACATTTGATGCGAAAAAGAACACAGGGGGTGCAAATGGTACAGTTA
GGTTGGAATTAAGCAGCCCCCCTAACAAGGGCATCAAATTCGCTGTTGATGTTTGTGA
ACAAGTCAAGGTTAAACACCCCCGAATTACCTATGCTGACCTTTTCCAGCTTGCCGGA
GTAGTTGCAGTTGAAGCTACAGGCGGACCTATCATCCAATATTCTCCAGGCCGACCTGA
CTCCCCGCAAAAAGACAGTACCGGAAATCTTCCAAACCCTAATGGAGATGCTCGGCAT
TTGAGAGATGTACTCGGCAAAATGGGATTTTCAGATAGGGAAATCGTAGCACTTTCAG
GATCTCATACTTTGGGTCGAGCACATCAAGATAGATCGGGTTTTGACGGTCCTTTTACC
AAAGAACCATTGAAATTTGACAATTCCTACTATGTAGAGTTGTTGCAAGGTGATACCCC
AGGACTAGTGAAGTTCCCCACAGACAAAGTTCTTATTCAGGATCCCGTGTTTCGTCAAT
TTGTTCAGCTCTATGCTCAGGATGAAAAGAAATTCTTTGAAGATTATGCAGAAGCGCAC
AAGAAGATGTCAGAGCTTGGAGTATTCCCACCTTCTGTGGTTGAAGATTGGATGCAAA
GACTCGGGCGAGTGGGAGGAGGAGGAGGTGGTGGAGGCGGCGCAACAGGAGTGGCG
GGGGAAGAGACAGAAACAGAGGAGGAATGA
>HpAPX10a
ATGGCAAAGGTTGATGCAGACTATTTGAAACAAATCGAGGCTGCTCGCAACGAGCTTC
GCAACATTATCCGCTCCCAAAATTGTGCTCCCATTATGCTCCGCTTAGCATTTCATGATG
CAGGGACATTTGATGCGAAAACAAAGACAGGAGGTCCAAATGGTACAATCAGGTTGG
AGTTAAGCAGCCCTCCAAACAATGGCATCAAAGTTGCTGTTGATCTTTGTGAACCAGT
GAAGGCTAAACACCCCAAAATTACCTATGCTGACATTTATCAGGACGCCCCGCAACAA
GACAGTACTGCCGGTCTTCCAAATCCTGGTGGAGATGCTGAACATTTAAGAGATGTATT
CCATAGAATGGGTCTTTCTGACAGGGATATTGTCGCACTTTCGGGTTCTCACACATTGG
GTAGAGCACATCAGGATAGATCAGGCTTCGACGGTCCGTTTACCCCAGAGCCTCTGAA
ATTTGACAATTCATACTATGTAGAGCTAAGGAAAGGTGAATCACCCGGATTGGTGAAAT
TTCCTAGTGACAAGGTTTTGACTCAAGATCCAGTCTTCCGCAAATATGTTCAGATTTATG
CTCAGGACGAGAAGACATTTTTTGCGCATTATGCAGAATCTCACAAGAAGATGTCTGA
GCTTGGATTCCGCCCACCAGTCAAGGCTTAA
>HpCAT1
ATGGATCCCTACAAGTTTCGTCCGTCAAGTGCACACAATTCTCCATTTGGACTACTAAT
TCTGGTGCTCCTGTTTATAACAACAATTCCTCGTTGACCGTTGGTAACAGAGGTCCCAT
TCTTTTGGAGGATTATCATCTAGTGGAGAAACTTGCCAACTTTGATAGGGAGCGTATTC
CAGAACGCGTTGTTCATGCCAGGGGAGCGAGTGCTAAGGGATTTTTTGAAGTCACCCA
TAATGTTTCTCACCTTACATGTGCTGATTTTCTACGTTCCCCTGGGGTCCAGACGCCTGT
TATTGTTCGCTTCTCCACTGTTATTCATGAACGTGGGAGTCCTGAAACACTGAGGGACC
CCCGTGGTTTCGCTGTGAAGTTTTACACCAGAGAGGGTAATTTTGATATGGTAGGGAAC
AATTTCCCTGTGTTTTTCATCCGTGACGGCATGAAGTTTCCTGATATGGTGCATGCGTTG
AAACCCAACCCCAAATCTCACATCCAAGAAAACTGGAGGATCCTTGATTTCTTCTCTC
ACCACCCTGAGAGTTTACACATGTTTACTTTTCTCTTTGATGATATTGGAATTCCTACCG
ACTATAGGCACATGGAAGGGGCTGGTGTCAACACTTACATGCTGATTAACAAGGCTGG
GAAAGCTCAATATGTCAAGTTTCATTGGAAACCAACTTGTGGAGTGAAATCTTTGTTGG
AAGATGAGGCCATAAAAGTTGGTGGGGCCAACCATAGTCATGCAACTCAGGACCTCTA
TGATTCAATCGCTGCCGGGAATTATCCCGAATGGAAATTGTTCATCCAAACAATTGACC
CGGTTGATGAAGATAAGTTTGACTTTGACCCACTAGATGTTACCAAGACATGGCCCGA

GGACATTTTGCCTCTACAGCCTGTTGGACGCCTAGTTTTGAACAAGAACATTGACAAC
TTTTTTGCGGAAAATGAGCAGCTGGCATTTTGCCCTGCACTAGTTGTTCCTGGTATCTA
CTACTCAGATGACAAGCTACTCCAGACTCGAATTTTCTCATATGCCGATACCCAAAGAC
ACCGTCTTGGACCCAACTACCTACAAATTCCTGTTAATGCACCAAAAAATGCTCATCAC
AACAATCACTTTGATGGGTTCATGAACTTTATGCACAGGGATGAGGAGGTTGATTACTT
CCCTTCTAGATATGATCCTGTTCGTCATGCTGAGAAATATCCCATTCCATCAAATGTCTT
GGGTGGTAGACGTGACAGGCAAGTCATTGTAAAGGAGAACAACTTCAAGCAGCCTGG
AGAGAGATACCGGTCCTTTGAACCCGCCAGGCAAGAGAGATTTATCCAAAGATGGGTT
GAGGCGCTATCTGATCCTCGTATCACTCATGAGATTCGCAGCATCTGGGTTTCATACTGG
TCACAGGCTGACAAATCGTTGGGCATGAAGCTAGCAAGTAGGCTTAATGTCAGGCCAA
CCATGTGA
>HpCAT1b
ATGGTAGGGAACAATTTCCCTGTGTTTTTCATCCGTGACGGCATGAAGTTTCCTGATAT
GGTGCATGCGTTGAAACCCAACCCCAAATCTCACATCCAAGAAAACTGGAGGATCCTT
GATTTCTTCTCTCACCACCCAGAGAGTTTACACATGTTTACTTTTCTCTTTGATGATATT
GGAATTCCTACCGACTATAGGCACATGGAAGGGGCTGGTGTCAACACTTACATGCTGAT
TAACAAGGCTGGGAAAGCTCAATATGTCAAGTTTCATTGGAAACCAACTTGTGGAGTG
AAATCTTTGTTGGAAGATGAGGCCATAAAAGTTGGTGGGGCCAACCATAGTCATGCAA
CTCAGGACCTCTATGATTCAATCGCTGCCGGGAATTATCCCGAATGGAAATTGTTCATCC
AAACAATTGACCCGGTTGATGAAGATAAGTTTGACTTTGACCCACTAGATGTTACCAAG
ACATGGCCCGAGGACATTTTGCCTCTACAGCCTGTTGGACGCCTAGTTTTGAACAAGA
ACATTGACAACTTTTTTGCGGAAAATGAGCAGCTGGCATTTTGCCCTGCACTAGTTGTT
CCTGGTATCTACTACTCAGATGACAAGCTACTCCAGACTCGAATTTTCTCATATGCCGAT
ACCCAAAGACACCGTCTTGGACCCAACTACCTACAAATTCCTGTTAATGCACCAAAAA
ATGCTCATCACAACAATCACTTTGATGGGTTCATGAACTTTATGCACAGGGATGAGGAG
GTTGATTACTTCCCTTCTAGATATGATCCTGTTCGTCATGCTGAGAATTATCCCATTCCAT
CAAATGTCTTGGGTGATAGACGTGACAGGCAAGTCATTGTAAAGGAGAACAACTTCAA
GCAGCCTGGAGAGAGAGATACCGGTCCTTTGAACCCGCCAGGCAAGAGAGATTTATCCAA
AGATGGGTTGAGGCGCTATCTGATCCTCGTATCACTCATGAGATTCGCAGCATCTGGGT
TTCATACTGGTCACAGGCTGACAAATCGTTGGGCATGAAGCTAGCAAGTAGGCTTAAT
GTCAGGCCAACCATGTGA
>HpCAT2
ATGGATCCCTACAAGTATCGGCCATCAAGTGCTTACAACACTCCTTACTTAACAACAAA
GACGGGTGAACCTGTTTATAATGACAACTCTGCTTTGACTGTTGGTCCTCGAGGGCCA
ATTCTGTTGGAAGATTACCATCTGATAGAAAAGATAACGACATGGACTCACGAGCGTAT
TCCGGAGCGAGTGGTACACGCAAGGGGAGCCAGTGCAAAGGGTTTCTTTGAAGTCAC
TCATGACATTACTCATCTTACTTGTGCTGATTTCCTTCGTGCTCCCGGTGTTCAAACTCC
TGTCATTGTTCGTTTTTCGACTGTCATTCATGAGCGTGGCAGCCCTGAAACAATCCGTG
ACCCTCGTGGTTTTGCCACCAAGTTCTATACCCGTGAGGGTAACTTCGATTTGGTAGGG
AACAATTTCCCAGTATTCTTCACCCGTGATGCAATGCAGTTCTTTGATTTGATCCGGGCC
TTCAAGCCAAACCCGAAATCCCACATCCAAGAGGACTGGAGGTTCCTGGACCACTGCT
CCTACCTCCCCGAGAGCCTCAACACCTTCACCTTCTTCTATGACGACATGGGTATCCCG
ATCAACTACCGACACATGATTGGGTCAGGTGTCCACACCTTCACTATGTTGAACAAGG
CAGGCAAGGTCACCTATGTCAAGTTCCACTGGAGGCCCACATGTGGTGTCAAGAACAT

GCTTGAGGACGAAGCTGCTAAAGTCGCTGGAGAAAACCACAGTCACGCGACAAAAG
ACCTTTACGACTCCATTGCCGCAGGAAACTACCCTGAATGGAAGTTCTACATCCAAACC
ATGGACCCGGCCGATGTGGATAAGTATGACTTCGACCCGTTGGATACCACCAAGACTTG
GCCTGAGGATATTTTACCGCTGCAGCCCGTGGGACGCATGGTGTTGAACAAGAACATT
GACAACTTTTTCAATGAAAATGAGATGCTTGCGTTTAACCCGGCTGCTATTGTTCCTGG
AATCCATTTCTCTGATGATAAGATGCTTCAGGCTCGGACTTTTGCTTATGCTGATACTCA
GAGATATCGGCTTGGTCCTAATTTCATGCAGCTTCCTGTTAATGCCCCGAAATGCCCTCA
CCACAATAATCATAGAGACGGGCCTATGAACATGGTTTTCAGGGATGAAGAGGTGGAC
TACTTCCCATCAAGATTTGATCCCGTTCGTGAGGCTGAGAGATTCCCCATCCCAAATTT
CGTAGTTCATGGAAGGCGCGAAAGGCAAATCATTCCGAAGGAGAACAACTTCAAGGA
ACCCGGTGACAGATACAGATCATGGGACCCAGCCAGGCAAGAGAGGTTTATTGGGAG
ATTCGTCAAGGCCCTGTCCGATCCTCGTCTAACTTATGAGATTCGCAGCATTTATGTTAA
CTGGTTCACTCAGGCTGATAAATCTCTAGGCATGAAAGTGGCAAGCAGACTCAATGTG
AGACCAACCATGTAA
>HpCAT1a
ATGGATCCCTACAAGTTTCGTCCGTCAAGTGCACACAATTCTCCATTTTGGACTACTAAT
TCTGGTGCTCCTGTTTATAACAACAATTCTTCGTTGACTGTTGGTAACAGAGGTCCCAT
TCTTTTGGAGGATTATCATCTAGTGGAGAAACTTGCCAACTTTGATAGGGAGCGTATTC
CAGAACGCGTTGTTCATGCCAGGGGAGCGAGTGCTAAGGGATTTTTTGAAGTCACCCA
TAATGTTTCTCACCTTACATGTGCTGATTTTCTACGTTCCCCTGGGGTCCAGACGCCTGT
TATTGTTCGCTTCTCCACTGTTATTCATGAACGTGGGAGTCCTGAAACACTGAGGGACC
CCCGTGGTTTCGCTGTGAAGTTTTACACCAGAGAGGGTAATTTTGATATGGTAGGGAAC
AATTTCCCTGTGTTTTTCATCCGTGACGGCATGAAGTTTCCTGATATGGTGCATGCGTTG
AAACCCAACCCCAAATCTCACATCCAAGAAAACTGGAGGATCCTTGATTTCTTCTCTC
ACCACCCAGAGAGTTTACACATGTTTACTTTTCTCTTTGATGATATTGGAATTCCTACCG
ACTATAGGCACATGGAAGGGGCTGGTGTCAACACTTACATGCTGATTAACAAGGCTGG
GAAAGCTCAATATGTCAAGTTTCATTGGAAACCAACTTGTGGAGTGAAATCTTTGTTGG
AAGATGAGGCCATAAAAGTTGGTGGGGCCAACCATAGTCATGCAACTCAGGACCTCTA
TGATTCAATCGCTGCCGGGAATTATCCCGAATGGAAATTGTTCATCCAAACAATTGACC
CGGTTGATGAAGATAAGTTTGACTTTGACCCACTAGATGTTACCAAGACATGGCCCGA
GGACATTTTGCCTCTACAGCCTGTTGGACGCCTAGTTTTGAACAAGAACATTGACAAC
TTTTTTGCGGAAAATGAGCAGCTGGCATTTTGCCCTGCACTAGTTGTTCCTGGTATCTA
CTACTCAGATGACAAGCTACTCCAGACTCGAATTTTCTCATATGCCGATACCCAAAGAC
ACCGTCTTGGACCCAACTACCTACAAATTCCTGTTAATGCACCAAAAAATGCTCATCAC
AACAATCACTTTGATGGGTTCATGAACTTTATGCACAGGGATGAGGAGGTTGATTACTT
CCCTTCTAGATATGATCCTGTTCGTCATGCTGAGAAATATCCCATTCCATCAAATGTCTT
GGGTGGTAGACGTGACAGGCAAGTCATTGTAAAGGAGAACAACTTCAAGCAGCCTGG
AGAGAGATACCGGTCCTTTGAACCCGCCAGGCAAGAGAGATTTATCCAAAGATGGGTT
GAGGCGCTATCTGATCCTCGTATCACTCATGAGATTCGCAGCATCTGGGTTTCATACTGG
TCACAGGCTGACAAATCGTTGGGTATGAAGCTAGCAAGTAGGCTTAATGTCAGGCCAA
CCATGTGA
>HpCAT2a
ATGTTTGGAGATTTAATTTTGGTGCAGTATCGGCCATCAAGTGCTTACAACACTCCTTAC
TTAACAACAAAGACGGGTGAACCTGTTTATAATGACAACTCTGCTTTGACTGTTGGTCC

TCGAGGGCCAATTCTGTTGGAAGATTACCATCTGATAGAAAAGATAACGACATGGACTC
ACGAGCGTATTCCGGAGCGAGTGGTACACGCAAGGGGAGCCAGTGCAAAGGGTTTCT
TTGAAGTCACTCATGACATTACTCATCTTACTTGTGCTGATTTCCTTCGTGCTCCCGGTG
TTCAAACTCCTGTCATTGTTCGTTTTTCGACTGTCATTCATGAGCGTGGGAGCCCTGAA
ACAATCCGTGACCCTCGTGGTTTTGCCACCAAGTTTTATACCCGTGAGGGTAACTTCGA
TTTGGTAGGGAACAATTTCCCGGTATTCTTCACCCGAGATGCAATGCAGTTCTTTGATC
TGATCCGGGCCTTCAAGCCAAACCCGAAATCCCACATCCAAGAGGACTGGAGGTTCCT
GGACCATTGCTCCTACCTCCCCGAGAGCCTCAACACCTTCACCTTCTTCTACGACGACA
TGGGTATCCCGATCAACTACCGACACATGATTGGGTCAGGTGTCCACACCTTCACTATG
TTGAACAAGGCAGGCAAGGTCACCTATGTCAAGTTCCACTGGAGGCCCACATGTGGTG
TCAAGAACATGCTTGAGGAGGAAGCTGCTAAAGTCGCTGGAGAAAACCACAGTCACG
CGACAAAAGACCTTTACGATTCAATTGCCTCAGGAAACTACCCTGAATGGAAGTTCTA
CATCCAAACCATGGACCCGGCCGATGTGGATAAGTATGACTTCGACCCGTTGGATACCA
CCAAGACTTGGCCTGAGGATATTTTACCGCTGCAGCCCGTGGGACGCATGGTGTTGAA
CAAGAACATTGATAACTTTTTCAATGAAAATGAGATGCTTGCGTTTAACCCGGCTGCTA
TTGTTCCTGGAATCCATTTCTCTGATGATAAGATGCTTCAGGCTCGGACTTTTGCTTATG
CTGATACTCAGAGATATCGGCTTGGTCCTAATTTCATGCAGCTTCCTGTTAATGCCCCGA
AATGCCCTCACCACAATGATCATAGAGACGGGCCTATGAACATGGTTTTCAGGGATGAA
GAGGTGGATTACTTCCCATCAAGATTTGATCCCGTTCGTGAGGCTGAGAGATTCCCCAT
CCCAAATTTCGTAGTTCATGGAAGGCGCGAAAGGCAAATCATTCCGAAGGAGAACAAC
TTCAAGGAACCCGGTGACAGATACAGATCATGGGACCCAGCCAGGCAAGAGAGGTTT
ATTGGGAGATTCGTCAAGGCCCTGTCCGATCCTCGTCTAACTTATGAGATTCGCAGCAT
TTATGTTAACTGGTTCACTCAGGCTGATAAATCTCTAGGCATGAAAGTGGCAAGCAGAC
TCAATGTGAGACCAACCATGTAA

CDS sequence of SOD, APX and CAT gene family members in *Beta vulgaris*

>BvCSD1
ATGGGCAAGGCTGTGGTTGTTCTAAACAGCAGTGAGGGTGTTACTGGAACTATCTACT
TCACCCAGGAAGGAGATGGTCCGACTACTGTAACTGGAAACATCTCGGGTCTTAAGCC
TGGGCTCCATGGATTTCATGTTCATGCTCTTGGTGACACGACAAATGGCTGCATGTCAA
CTGGACCACATTATAACCCTGCTGGGAAAGAGCATGGTGCTCCAGAAGACGAGGTTCG
CCATGCTGGTGATCTTGGGAATGTCACAGTTGGGGATGATGGTACTGCGACCTTTACAA
TTATTGACAGCCAGATTCCTCTTTGTGGACCCAACTCAGTTGTGGGGAGGGCTGTTGTT
GTCCATGCTGATCCTGATGATCTTGGAAGGGGGGGACATGAGCTCAGCAAGACCACAG
GCAATGCTGGTGGAAGAGTGGCTTGTGGTGTTATTGGTCTTCAAGGTTAA

>BvCSD3
ATGGGCACAACAGTGAAGGCAGTGGCTGTCATCGCCGGTTCCAATAACGTCAAAGGCT
CTCTCCATTTCATCCAACATTCCACTGGGATAACACATGTCAAAGGAAAAATAACAGGA
CTTACACCAGGTCTCCATGGCTTTCATATCCATGCCCTTGGAGATACCACTAATGGTTGC
AACTCCACTGGGCCTCATTTTAATCCGATGAAGAAGGACCATGGAGCTCCATCTGACA
CGGAACGCCATGTGGGTGACCTGGGAAACATTGTTGCTGGCAGTGATGGGGTTGCTGA
GGTCTCCATTAGTGATTCCCAGATATCACTTAGTGGGCAGCACTCAATACTTGGGAGGG
CTGTTGTGGTGCATGCTGATCCTGATGATCTTGGACGAGGTGGACATGAACTTAGCAAA
GCAACTGGGAATGCTGGTGCTAGAGTAGGATGCGGTGTAATCGGGCTGCAATCATCCG
TTTAG

>BvCSD2

ATGCAAGCAGCATTGGCAGCCATGACAGCGCACACCATTCTCGCTTCCCACCCTACATT
ATCTCTTGTCTCTCCTTTTTCCTCACCCACTACAAGTACAATCTCAACTTCTCTTAACTC
CTCTACCTCTTCCTTCAATGGCCTCTCTTTCAAGCTTTCCTCTCAAATACAATCTCTCTC
TGTGTCTAGTTCTGTGGCCCCTAAACCCCTCACCATTGTTGCCGCCTCCAAAAAAGCTG
TTGCTGTTCTTAAGGGCACTTCCAATGTTGAGGGTGTCGTCACTTTAACCCAAGAAGAT
GATGGACCAACTACAGTCAACGTTCGTATTACTGGACTTACTCCTGGGAAACATGGATT
TCACTTGCATGAATTTGGAGATACAACAAATGGGTGTATTTCTACAGGGCCACATTTTA
ATCCTAGTAGCATGACACATGGTGCTCCGGAGGATGAAATCCGGCATGCGGGTGACCT
GGGAAACATTGTTGCTAACACAGATGGAGTGGCTGAGGCTACCATCATTGATAATCAG
ATTCCGTTGAGTGGAGCAAATTCTGTTGTTGGTAGAGCCCTTGTGGTCCATGAACTTGA
AGATGACCTTGGCAAGGGTGGACATGAACTGAGTCTGACTACTGGGAATGCTGGTGGC
AGATTGGCTTGTGGTGTTGTTGGTTTAACTCCTCTGTGA

>BvCSD4

ATGACGTTTCTAAGGTCAATAGCCACAACCACGACCGCCATAGCAGTCACAGCTCTAC
CTGCATCTTACGTTGCATCTTCTCTCTCTCACTCCTCTTCTTCATCTTCATCTTCATCTTC
TTCTCCATTCTCGAAATTTCTGGACTTCCATTTTCTCTCTCCTCGATCATCTTTGAGCTCC
TGCGCAGGCAATAAATTAGGGCTACTCAAGAATTTGAGCTCACCGCCTTCTGCTGTTCA
AATGGAAAATCCTAAATCTTCTTCTTATGAAAATATGATGTTGCCGGAATTAATGACAGA
GTTCATGGTGGATATGAAGTGTGAAGGCTGTGTAAGTGCTGTCAAGAGTAAACTTGAG
ACTCTAGAAGGAGTTAAGAATGTTGAAGTGGACTTGAGCAACCAAGTTGTCAGAATTC
TTGGTTCTTCACCCGTGATAACCATGGAAAATGCTTTGGAGCAAACTGGTCGTAAAGC
TCGACTGATTGGTCAAGGTGTACTTGAAGATTTCCTTGTGTCCGCGGCAGTTGCAGAAT
TTAAAGGCCTAGATATCTTTGGAGTGGTTCGCTTGGCCCAGACTCACATGGAGTTGGCT
AGAATAGAGGCCAATTTTAGTGGTTTGCCACCAGGGAAACATGGATGGTCTATAAATGA
GTTTGGTGATCTGACTAGAGGTCCAGTCAGCACGGGGAAGGTTTACAATCCACCAATC
CAAGGAAGTGAAAATGAGCCATTGGGTGACCTGGGAACATTAGAGGTTGATGAGAAA
GGTGAAGCATATTATACAGGAATCAAGAAAATGTTAAGGGTTGTTGATTTAATTGGACG
GTCCATTGTCTTGTATGCATCGGAGGACAAATCCGATCCTGGTCTTGCAGCTGCCGTGA
TCGCGAGGAGTGCAGGGGTAGGTGAGAACTATAAGAAGCTTTGCACATGCGATGGAA
CCACAATTTGGGAGGCGAAGCCTGATTTTGTCACTACTCACTAG

>BvFSD1

ATGTTGTGTCCTATAACTCTATACCACGTTGGCACGTTTGGCTTTTATAAGAGTGTGTTC
GGCACAACCAAACTGCTATCTCCTCAAACACAAACACTGCTGACTGCTGTTTTTGATA
CTTCTATCAAAGAAATTATGTCTACTGTTGCTGCAACTTCCGCGATATCTTTGTCTTCAA
ACTTTCTCTCTCATGAAGGATTTAAAGGGGAACTCAATTCTATCTGTGTCTCACCATGG
AGGAAACAAACAAGAGTAGGAAGAGCTGGTGCTGCTGTTGTTACAGCCAAATTTGATT
TGAAGCCCCCACCTTATCCATTTGATGCCTTGGAGCCTCACATGAGCAAGCAAACCTTT
GACTATCATTGGGGGAAGCATCATAGAGCCTATGTGGATAACTTGAACAAGCAAATAGT
TGGAACAGAATTAGATGGATTGCCACTTGAAGAAGTAGTACGTATCACATATAACAAAG
GAGATGTACTTCCTTCTTTTAATAATGCAGCACAGGCCTGGAACCATGAGTTCTTTTGG
GAATCCATGAAGCCTAGTGGTGGGGGAAAGCCTTCCGGCGAGCTTTTGGCGCAAATTG
AAAAGGATTTTGGTTCTTTTGAAGCATTTGTTACTGAATTCAAGACTGCTGGGGCTACA
CAGTTTGGTTCTGGCTGGGCTTGGCTTGTTTACAAAACAAATAAATTGGATGTTGGGAA

CGCAGTAAATCCGAAGCCATCTGAAGATGACAAGAAGCTTGCAGTTGTTAAGAGCCCA
AATGCTGTGAACCCTCTCGTTTGGGACTATTATCCACTTCTTACTGTCGATGTTTGGGAG
CATGCTTATTACTTGGACTTCCAGAACCGAAGGCCTGATTATATCTCAATTTTCATGGAA
AATCTTGTTTCCTGGGATGCTGTCAGTGTAAGATATGAGGCAGCCAAGGCTGTTGCTGC
TGACAGGGAGAAAGAAACAGCACAGAATTAA
>BvMSD1
ATGGCGCTTCGAGTTTTGATGACAAGAAAAACCCTAGCTTCTGCAAAGCAAGGCCTTC
TTCAATGTCGATCATTGCAGACTTTCTCTCTCCCTGACCTTCCTTACGACTATGGTGCTC
TTCAGCCCGCCATTAGCGGCGAAATCATGCAGATTCATCATCAGAAACATCACCAGACT
TACATCACTAATTACAATAAAGCTCTTGAACAACTCGATGATGCTATTGCTAAAGGCGA
CGCTTCTTCTGTCGTTAAGCTTCAAAGCGCCATTAAATTCAATGGCGGAGGTCATGTGA
ATCACTCAATTTTCTGGAAGAACCTTGCTCCAATTAATGAAGGAGGTGGTGAGCCTCC
AAAATCTTCACTGGGTTGGGCTATTGATTCAAACTTTGGTTCTCTGGAAGCTCTTATTC
AGAAAATGAATGCAGAAGGAGCTGCTGTACAGGGTTCTGGGTGGGTGTGGCTTGGAC
TCGACACGCAATCAAAAAAGCTTGTGGTCGAAACCACGCCAAATCAGGACCCACTCG
TAACTAAAGGACCAAGCTTGGTTCCTATACTTGGTATAGATGTATGGGAGCATGCGTAC
TACCTTCAGTACAAGAATGTGAGACCTGATTATCTGAAGAACATCTGGAAGGTTATAAA
CTGGAAATATGCAAGCGAGGTGTATGAAAAAGAGGTCCCCAGCCACTGA
>BvFSD2
ATGGGAAGTTTTAGTGCCATAATTTCTGTTAGCTCACCTCTTCATGCAAATGAATTGTTT
CCCAAGATTAAAAATTTAACCTTTTATAATCAGCAGAAAAGAAGAAATCAAGGACTGA
AAAGAAGGCCTGGAGTTGTTGCATACTATGGGCTGAAAGAGCCTCCCTATAACCTTGAT
GCTCTAGAACCATATATGAGCCAAAAAACACTGGAGTCGCATTGGGGAGAGTATCATA
GAGGTTACTTGGAAAATTTGAATAGACATTTGAGTAGGAGTGACATACTATATGGCTATA
CTATGGATGACCTTGTGAAAGTAACATACAATTATGGAAACCCCTTACCTGAATTCAAC
GATGCTGCACAGGTCTGGAATCACGACTTCTTTTGGGAATCCATGCAACCTGGAGGTG
GGGAGTCTCCAACGCTTGGTCTTCTTCAGCAGATTGAAAAGGATTTTGGTTCTTTTACA
GACTTCAAAGAGAAGTTCATAGAGGCAGCCATGACACACTTTGGTTCTGGCTGGGTTT
GGCTGGTTTTGAAGAGGAAGGAGAAGCAGCTTGCAGTGGTGAAGACCTCGAATGCAG
TAACTCCTATCGTTTGGGATGATATCCCAATCATATGCCTGGATTTGTGGGAGCATGCCT
ACTACTTGGATTATAAGAATGATAGAGCGAAGTATGTTAATGTTTTCATGAACCACCTTG
TATCATGGCATTCGGCAACAGCACGCCTTGCTCGAGCACAGGCTTTTGTGAATCTTGGA
GAACCTAAAATTCCAGTCGCTTGA
>BvAPX9
ATGGTGAATGCAGGATACTTGAAGGAAATAGAGAGAGCTCGTAGAGACCTTCGCGCTC
TCATCTCTGCCAAGCAATGTGCTCCTATCATGCTTCGTTTAGCATGGCACGATGCAGGTA
CATATGATGCAAAGACGAATTCAGGGGGTCCGAATGGCTCAATTAGGAACGAGCACGA
GTTGAGGCACAATGCCAACAATGGCCTCAAAATCGCGGTTGATCTTTGTGAAGAAGTT
AAGGCTAAGCACCCTCAGATTACTTATGCAGACCTTTATCAGCTTGCTGGAGTTGTTGC
TGTGGAAGTCACAGGCGGGCCAACGATTAACTTCGTTCCAGGTCGAGTGGATTCCTCA
GTTTCTCCAGATGAAGGACGTCTTCCAGATGCAAATCAAGGTGCTAGGCATTTGCGAG
ATATATTTCATAGAATGGGGCTCTCTGACAAAGATATTGTGGCACTGTCAGGAGGCCAC
ACACTGGGTAAAGCACATAAGGACAGATCAGGTTTTGAAGGCCCTTGGACCAGCAATC
CATTGAAGTTCGACAATTCATATTTTGTAGAGTTGCTGAAAGGTGAACAATGGGGGTTA

TTGAAACTTCCCACTGATAAGGTTCTTACACAGGATCCTCACTTTCGTCCCTATGTTCAT
CTCTATGCTAAGGATGAAGACGCGTTCTTCAAAGACTATGCAGAATCGCACAAGAAGC
TATCAGAGTTGGGGCTTACTCGATCCTCTTCTTTGAAGATGACTAAGGCTACTATTGGA
GTTCTTGTCACTCTTACCGTAATTATCTTAAGCTACTTGTATGATTTAAATAAACAAGCAT
AA
>BvAPX10
ATGGCACAAGCACAGTTGCACATTGTGGATGCTGAATATTTGAAACAAATTGAAGGGG
CTTGTAAAGAGATTCGCAACCTGCTACAGAACAACAAGCAATTTGCTCCCATCGTGCT
TCGCTTAGCATTTCATGATGCAGGAACTTATGATGCCAAAGCCAAGACTGGAGGTCCA
AATGGTTCCATCAGGAATGAGTTGAGCAACCCTGCCAACAATGGTATCAAAACCGCTA
TTGATTTTTGCGAGCCAGTGAAGGCTAAATATCCTAAGGTTACTTACGCTGACCTTTATC
AGCTTGCTGGTGTAGTTGCAGTTGAAGTTACTGGAGGGCCTATTATCCCCTTTGTTGCG
GGTAGACCAGATGCCCCGGATCAACAAGACAGTGGAGCTCTGCCAAATCCCAATGGA
GATCTCAAGCATGTGAGAGACATTTTCTATCGAATGGGCTTGAACGATAGGGATATCGT
AGTTCTATCAGGGGCTCACACACTGGGTCGAGCACACAAGGATAGATCAGGCTTTGAT
GGTCCTCTCAGCAAAGATCCATTGAAGTTTGACAATTCCTACTTTGTCGAATTGAGGAT
GGGTGATACTCCTGGACTGGTGAAATTCCCAACAGACAAAGCTCTTTTGGAGGACCCT
ATTTTGCGTCGCTATGTTCAAATTTATGCCAAGGATGAAAAGGTATTTTTCGCACATTAT
GCTCAATCACACAAGAAGATGTCTGAGCTCGGGTTTCACCCACCTCGTTGTCCTAAGA
ACGCTTGA
>BvAPX2
ATGGGGAAGAGTTACCCAAGTGTTAGCAAGGAATACAAGGAAGCAGTAGACAAATGT
AAAAGAAAGCTTAGAGCTCTCATCCAAGAGAAGCACTGTGCTCCCATCATTCTTCGTT
TGGCATGGCACGCAGCTGGGACATTTGACGTTCAGTCAAAAACGGGTGGTCCATTTGG
GACAATAAAGAACCCACAGGAGCTGGCACATGTTGCAAATAATGGCTTGGATATTGCT
GTTAGACTACTGGAACCAATCAAAGAGCAGTTCCCAATCCTAAGCTATGCTGACTTCTA
TCAGTTGGCTGGAGTGGTTGCAGTAGAAATTACAGGGGGGCCTGAGATCTTGTTTCAC
CCTGGAAGACCTGATAAAACTGAATCGCCGCCAGAGGGTCGCTTACCTGATGCGAAGA
AAGGTTCTGACCATTTGAGGGAAGTATTTTACCGTATGGGTTTGTCTGACAAGGATATT
GTTGCTCTATCTGGTGCTCATACCTTGGGAATGTGTCATAAGGAGAGATCGGGCTATCA
GGGAGCTTGGACTAACAATCCTTTGATCTTCGATAACTCGTATTTCAGAGAGCTTCTTA
CGGGAGATAAAGAAGGTTTCATTCAGCTGCCATCGGACAAAGCACTCCTTGAGGATGT
AGCATTTCGTCCCTATGTTGAAAAATATGCACAGGACGAGGAAGCTTTTTTTGCGGATT
ATGCTGAAGCACATTTGAAGCTTTCTGAGCTTGGATTTGCAGACGAGGTATGA
>BvAPX6
ATGACGACGGTCGGCGGCGAATCTTCCATTTCACGCTGCTGTTTTGCCACCCAATCATC
ATCTTCTCCCCTCAACAACTTCAAATTTCCCGCCAAATCTTCTCCGTCAACTGTCCAATT
TGGCGCCAAAGCTTCTCCATTTCTCACTCCTACTTTTGCACTTCCACCCATTTCAGATGA
ATGTAGCAATGTCAACTCGCGTGGTAGAAGAGGACTCATTTTCCTTTCTGCCACTCTTC
CTTTTCTTTTTCCTTTGCGAGATTTTGTCGATGATCTCATTGTTGAAGCAGAAGAGCTGG
GAGCTCCTGATTACCTACTTATAAAGGATGAGATAAGGAAGGTTTTGACAAAAGGAAA
GGCAGCTGGTGTACTTCGTCTTGTGTTTCATGATGCAGGAACTTTTGAGCTCAGTGAGA
ACTCAGGAGGTATGAATGGCTCCATAACATATGAACTCGAGAGGCCTGAGAATAAAGG
GCTCAAAAAATCTCTCAAGATTGTTGAAAAAGCGAAAAATGTGGTGGATGAAAGACT

GTCAGTGTCATGGGCAGACATGATAGCTGTAGCAGGAGCTGAGGCAGTCTCGATTTGT
GGAGGTCCAGAGATCCCTCTTCGGTTGGGGAGACTTGATTTGCCGAATCCTGATCCTG
AAGGGAAACTACCTGAAGAATCACTGGATGCGCTAAGGTTGAAGCAGTGCTTTCAAA
GAAAAGGCTTTTCGACACAAGAACTTGTTGCACTTTCTGGGGCTCATACTCTTGGAAG
TAAAGGTTTTGGAAGCCCTGTTGTATTTGACAATTCATATTACAAAATACTCCTTGAGAA
ATCTTGGATGTCATCAGGTCAGCCAGCTGGAATGTCAAGTATGGTTGGACTTCCTTCTG
ATCGTGCACTAGTCGAGGATGATGAATGTTTAAGATGGATTACAGATTACGCGAACGAC
CAGGATAAGTTTTTTGAGGATTTTAAGAATGTTTACATCAAGTTAGTCAATTCAGGTGC
AAGGTGGAAAGCTTTATGA
>BvAPX1
ATGGGAAAGAGCTACCCAACTGTCAGCGAGGATTACCAGAAGTCAATCGAGAAGGCC
AAGAGAAAGCTCAGGGGTTTAATTGCGGAGAAGCATTGTGCTCCTATTATGCTCCGTCT
TGCATGGCACTCTGCTGGTACCTTTGATGTGCAGTCAAGGACTGGAGGACCTTTCGGT
ACAATGAGGCACAAGGGAGAGTTGGCTCATGGGGCTAACAACGGCCTTGATATTGCTG
TTAGGTTGTTGGAGCCCATCAAGGAACAGTTTCCCGAACTATCTTATGCTGACTTTTAC
CAGTTGGCTGGAGTTGTGGCTGTTGAAGTTACTGGAGGCCCTGAAGTTCCCTTCCACC
CAGGAAGAGAGGACAAGCCAGAGCCACCCCAAGAAGGCCGTCTCCCTGATGCCACTA
AGGGTTGTGACCACTTGAGGGACGTCTTCATTAAGCAAATGGGTCTTACTGACCAGGA
CATCGTAGCTCTCTCTGGAGGCCACACCTTGGGAAGGTGCCACAAGGACCGCTCTGGT
TTTGAGGGCCCTTGGACCTCTAACCCTCTGGTTTTCGACAACTCCTATTTTACGGAGCT
CCTAACTGGTGAGAAGGAAGGTCTTTTGCAGCTGCCGTCTGACAAGGCTCTTCTTTCT
GACCCCGTCTTCCGTCCACTGGTTGAGAAATATGCCGCTGATGAGGATGCATTCTTTGC
CGACTATGCTGAGGCTCACCTGAAACTTTCTGAACTTGGGTTTGCCGATGCTTAA
>BvAPX7
ATGACTGATCGTTTATCAATGGCATCTCTCACTACTACTTCCGCCGCCGCTGCAGCAGC
AGCCTCTCGTCTTCTTCCTTCTTCCTCTATCTCTCGTCTTTCTCTCTCCTATTCATCGTCT
TCTTCTTCTTCTCTCAAATGTCTCCGATCATCTCCTCTCGTTTCTCATCTCTTTCTTCGAC
AGAGAGAAGTTTCAGCTTATGTGACGAAGACGAGGTTTAGTACGAAATGCTACGCTTC
GAATCCTGAGCAATTGAAGAATGCTAGGGAAGATATTAAAGAGCTTCTTAAGACCACG
TTTTGTCATCCTATTATGGTTCGCTTAGGTTGGCATGATGCTGGTACTTATAACAAGGAC
ATTGAAGAATGGCCACAAAGAGGTGGAGCCAACGGCAGCCTGAGGTTTGAAGTTGAG
CTCAAACATGGAGCTAATGCAGGTCTTGTAAATGCCTTGAAACTTCTCCAACCCATAAA
AGACAAGTACTCTGAAGTGACATATGCAGATCTGTTTCAGCTGGCTAGTGCTACTGCAA
TAGAGGAGGCTGGTGGTCCTAAGATACCCATGAAGTATGGAAGAGTGGAGGTGACCG
GGCCAGAGCAATGCCCAGAAGAAGGAAGACTTCCTGATGCTGGACCACCTTCACCTG
CTCAACATCTACGTGATGTGTTCTACAGAATGGGTCTAAATGATAAGGACATAGTTGCT
TTATCTGGAGCACATACATTGGGAAGGTCTAGACCTGAACGCAGTGGTTGGGGCAAGC
CAGAGACTAAATACACGAAAGATGGACCAGGAGCTCCAGGAGGACAGTCATGGACGG
CGCAATGGTTGAAGTTTGATAATTCCTATTTTAAGGACATCAAAGAAAAGAGAGATGCA
GATTTGCTTGTTTTGCCAACTGATGCTGCTCTATTTGAAGATCCATCGTTCAAGGTATGT
GCTGAGAAATATGCGGCTGATCAAGAAGCATTTTTCAAGGACTACGCTGAATCCCATGC
CAAACTCAGCAACCTTGGAGCCAAATTTGACCCTCCTGAGGGTTTCTCCTTAGACGGA
AGTCCTGCAGGAGCAGCCCCAGAGAAATTTGTAGCAGCCAAGTATTCATCTGGCAAGA
GAGAGCTTTCGGATTCTATGAAGCAGAAGATTCGCGCTGAATATGAAGGTTTGGGTGG

TAGCCCTGATAAGCCTCTCTCTTCAAACTATTTTCTTAACATTATGATTGTTATTGGCGTT
TTGGCTGTTCTATCATATCTTGCAGGAAATTAA
>BvAPX3
ATGGCGATGCCAGTAGTTAACACAGAGTACCTTAAAGAAATCGACAAAGCTCGTCGCG
ATCTTCGCGCACTCATCTCTAATCGCAATTGCGCTCCTATTATGCTCCGCCTCGCGTGGC
ATGATGCAGGGACGTATTGCGCCAAGACCAAGACTGGTGGTCCGAATGCTTCCATTCG
GAATGATGAAGAGTGCGCTCATGGTGCTAATAATGGTTTGAAGAAAGCTATTGATTGGT
GTGAGGAGGTGAAGTCTAAACACCCAAAAATTACTTATGCAGACCTCTATCAGCTTGC
TGGCGTTGTTGCAGTCGAGGTCACCGGAGGACCTACTGTTGACTTTGTTCCTGGTAGA
AAGGACTCAAATGTGTGTCCCAAAGAAGGACGACTTCCAGATGCCAAACAAGGTGCA
CCACATTTGAGGGACATATTCTATAGGATGGGTCTCACTGACAAAGACATTGTGGCCCT
GTCCGGGGGTCATACCCTGGGAAGGGCACATCCAGAGAGGTCAGGCTTTGATGGCCCA
TGGACCCAGGATCCGCTCAAGTTTGATAACTCCTATTTTCTGGAGCTATTAAAAGGAGA
ATCAGAAGGACTGTTGCAACTTCCTACTGATAAAACTTTGGTGGAGGATCCTGCATTTC
GCCCTTTTGTGGATCTGTATGCTAAGGATGAGGAGGCCTTCTTCCGGGACTATGCTGCC
TCACACAAGAAACTTTCAGAACTAGGATTTACTCCTAGTATTGGGGCCAAGTCTCCCA
AGGACAGCACTGTATTGGCCCAGAGTGCTGTTGGAGTTGCAGTTGCTGCAGCAGTAGT
CATTCTGAGCTACATCTATGAAGCTCGTAAGCGTGTGTAG
>BvAPX10b
ATGGCAGGAGCAAAGTCTATAGTAGATGCAGAATATCTGAAGCAAATTGAGGGTGCTC
GAAAAGAACTTCATAATCTTCTGAAGAACAAGCAATTTGCTCCCATCGTGCTTCGCTTA
GCATTTCATGATGCAGGTACTTATGACGCTAAAGCAAAGACAGGGGGTGCAAATGGTT
CAATTAGGAATGAGTTGAACAGCCCTGCCAACAATGGTATCAAAGTTGCTGTTGACTTT
TGCGAGCAAGTGAAGACTAAATTCCCAAAGGTTACTTATGCTGATCTTTATCAGCTTGC
TGGTATGATTGCAGTTGAAGTTACTGGGGGGCCTATTATCCCTTTTATCCCCGGTCGACC
AGATGCTCCGAAACAACAAGATGGTGGAGCTCTTCCAAATCCCAATGGAGATGCGCAA
CATCTGAAGGATGTATTCTATCGAATGGGCTTGAACGACAGGGATATTGTGGTTCTATCA
GGGTCTCATTCACTGGGTCGAGCAAACAAGGATAGATCGGGATTTGATGGTCCTTTCAC
CCAGGATCCTTTCAAATTTGACAATTCCTACTTTGTAGAGTTGAGGAAGGGTGACAGTC
CTGGACTGGTGAAATTCCCCACAGACAAAGTTCTCGCAGATGATCCTTCTTTCCGTCA
ATATGTTCAACTATATGCTAAGGACGAAAAAGCATTCTTCGCACATTATGCGGAGTCAC
ACAAGAAGATGTCGGAGCTGGGGTTCAACCCACCCAAGTTGCCAGTAAAAGCTTGA
>BvAPX1a
ATGGGAAAGTGCTACCCCACTGTTAGCGAGGATTACCAGAAGTCTATCGAGAAGGCCA
AGAGAAAGCTCAGAGGTTTAATTGCAGAGAAGCATTGTGCTCCTATTATGCTCCGTCTT
GCATGGCACTCTGCTGGTACTTTTGATGTGCAGTCAAGGACTGGAGGACCCTTTGGTA
CAATGAGGCACAAGGGAGAATTGGCTCATGGGGCTAACAACGGTCTTGATATTGCTGT
TAGGTTGTTGGAGCCCATCAAGGAACAGTTTCCCGAACTATCTTATGGCGACTTTTACC
AATTGGCTGGAGTTGTGGCTGTTGAAGTTACAGGAGGCCCTGAAGTTCCGTTCCACCC
AGGAAGAGAGGACAAGCCAGAGCCACCCCAAGAAGGCCGTCTCCCTAATGCCACAAA
GGGTTCTGACCACTTGAGAGACGTCTTCATTAAGCAAATGGGTCTTAATGACCAGGAC
ATTGTAGCTCTCTCTGGAGGCCACACCTTGGGAAGGTGCCACAAGGACCGTTCGGGTT
TTGAGGGTCCTTGGACCTCTAACCCTCTGGTTTTCGACAACTCCTACTTTACGGAGCTT
CTAACTGGTGAGAAGGAAGGTCTCCTACAGCTGCCATCTGACAAGGCTCTTCTTTCCG

ACCCTGTCTTCCGCCCACTGGTTGAGAAATATGCAGCTGACGAGGATGCATTCTATGCC
GATTATGCTGAGGCCCACCTGAAACTGTCTGAACTTGGGTTTGCTGATGCTTAA
>BvAPX10a
ATGGCAAACGCGCAATTGCACATTGTGGATTGTGAATATCTGAAACAAATTGAGGGAG
CTCGTCGAGATCTTCATGATCTGCTGCTGAACAACAAGCAATTTGCTCCTATCGTGCTT
CGCTTAGCATTTCACGATGCAGGTACTTATGACGCCAACGCGAAGACAGGGGGTCCAA
ATGGTTCCATTAGAAATGAGTTGAGCAACCCTCCTAACAATGGTATCAAAAATGCTATT
GATTTTTGCGAGCAAGTGAAGACTAAACACCCCAAGGTTACTTATGCTGACCTTTATCA
GCTTGCGGGTGTAGTTGCGGTTCAAGTTACCGGAGGCCCTTTTATTCCCTTTATCCCGG
GTCGACCAGATGCCCCGGATCAACAAGACAGTGGAGCTCTTCCAAACCCCAATGGAG
ATGGCCAGCATCTCAGAGATATATTTTATCGAATGGGCTTGAACGATAAGGATATCGTGG
CTCTATCAGGGGCTCACTCACTGGGTCGAGCACACCAAGATAGAACACCTGGCTTTGA
TGGCCCCTTCACTGAAGATCCCTTGAAATTTGACAATTCCTATTTTGTAGAGCTGATGA
AGGGTGATACTCCTGGACTGGTAAAGTTCCCTACAGACAAAGTTCTTTTGGAGGACCC
TGCTTTTCGTCAATTTGCTGAACTGTATGCCAAGGATGAAAAGGTGTTCTTCGCAGATT
ATGCACAATCGCAAAAGAAACTATCGGAGCTCGGGTTTAACGCACCACGTTGA
>BvAPX10c
ATGGGGAGAGTACCAGTGGTAAACGAAAACTATTGCAGGGTCATTGAGGCTGCTCGTA
GAGACCTGCGTTCTGTCATCCAAAACAATAAGAACGCAGCACCTATTCTCCTCCGCTTA
TCATTTCATGATGCAGGAAACTATGATGCTAGAGCAAAGCGCGGAGGTGCAAATGGTT
CAGTTAGGTTTACTCAAGAGTTGAACCGTCCCGTCAACAAGGGAATGGAAAACGCTAT
TAGACTTTGTGAACCAGTAAAGCGCAGACACCCTGATATTACATATGCTGACCTTTATC
AGCTTGCGGGAATTGTTGCAGTTGAGGTAACTGGAGGTCCTGCTATTGAATTTGTTCCA
GGTCGAGCTGATATCGATTCACCCGATGCAGACGGAATTCCGAACCCTAGAGGAGGAG
TGGATCATCTGAGAACAACATTCTACCGAATGGGCCTCAATGACAAAGATATCGTGGTA
CTTTCAGGGGCCCATGCACTGGGTGGAGCACATAAGGACAGATCAGGCTTTGATGGCA
ATTTCACCCAAAATCCTCTCAAGTTTGATAACTCCTACTTTGTGGAGCTGCTAAGGGGT
GATACTCCAAGATTGGTGAAACTACCCACAGATAAGGCTCTCTTAAACGATTCCGTCTT
CCGTCGCTTTGTTGAACTTTACGCTAGGGATCAAAGAGCATTTTTCAGAGATTACGCAG
AATCGCACAAGAAGCTATCAGAGCTAGGGTTCAATCATCAAGAATCCGACTTATATGAA
GATGACAGCTGCGACGAGTCGGTTGGTTGTGCGCAGTCCTATGCAGTTGAAGATGGCT
GCTGTCCTGCGCAGTTTGGGTATGCTCGACCCTATGCTGTTGGAAATGGCTGTCCAGCT
AACTATTACTAA
>BvAPX10d
ATGGGGAGAGTACCAGTGGTAAACGAAAACTATTGCAGGGTCATTGAGGCTGCTCGTA
GAGACCTGCGTTCTGTCATCCAAAACAATAAGAACGCAGCACCTATTCTCCTCCGCTTA
TCATTTCATGATGCAGGAAACTATGATGCTAGAGCAAAGCGCGGAGGTGCAAATGGTT
CAGTTAGGTTTACTCAAGAGTTGAACCGTCCCGTCAACAAGGGAATGGAAAACGCTAT
TAGACTTTGTGAACCAGTAAAGCGCAGACACCCTGATATTACATATGCTGACCTTTATC
AGCTTGCGGGAATTGTTGCAGTTGAGGTAACTGGAGGTCCTGCTATTGAATTTGTTCCA
GGTCGAGCTGATATCGATTCACCCGATGCAGACGGAATTCCGAACCCTAGAGGAGGAG
TGGATCATCTGAGAACAACATTCTACCGAATGGGCCTCAATGACAAAGATATCGTGGTA
CTTTCAGGGGCCCATGCACTGGGTGGAGCACATAAGGACAGATCAGGCTTTGATGGCA
ATTTCACCCAAAATCCTCTCAAGTTTGATAACTCCTACTTTGTGGAGCTGCTAAGGGGT

GATACTCCAGGATTGGTGAAACTACCCACAGATAAGGCTCTCTTAAATGATTCCGTCTT
CCGTCGCTTTGTTGAACTTTACGCTAGGGATCAAAGAGCATTTTTCAGAGATTACGCAG
AATCACACAAGAAGCTATCAGAGCTAGGGTTCAATCATCAAGAATCGGACTTATATGAA
GATGACAGCTGCGACGAGTCGGTTGGTTGTGCGCAGTCCTATGCAGTTGAAGATGGCT
GCTGTCCTGCACAGTTTGGGTATGCTCGACCCTATGCTGTTGGAAATGGCTGTCCAGCT
AACTATTACTAA
>BvCAT2
ATGGATCCGACTAAGTATCGCCCGGCAAGTGCTTACAATTCCCCGTACTTAACCAACAA
TAATGGTACACCTGTTTGGAACGATAACTCTTCGTTAACTGTTGGCCCCAGAGGTCCCA
TTCTGTTGGAAGATTATCATTTGATAGAAAAGATAGCAACTTGGACCCATGAGCGTATA
CCTGAACGTGTTGTACATGCTAGGGGAGCAAGTGCTAAGGGGTTTTTTGAAGTTACCC
ATGACATTGCACATCTTACGTGTGCTGATTTCCTACGTTCCCCCGGTGTTCAGACGCCA
GTTATTGTTCGGTTTTCAACTGTTATTCATGAACGTGGTAGTCCGGAAACACTCAGAGA
TCCTCGTGGTTTCGCTGTTAAGTTCTATACTAGAGAGGGTAACTTTGATATAGTTGGAAA
CAACTTCCCAGTGTTTTTCACCCGTGATGCAATGCAATTTTTTGATCTAGTCCGAGCATT
CAAACCCAACCCAAAATCCCACATCCAAGAAAGTTGGAGGTACATGGATTTCTGTTCG
TACCTCCCAGAGTCTCTCAACACCTTCTCATTCTTCTTCGACGACGTTGGTATCCCTCTA
AACTACCGTCACATGAACGGTTCCGGTGTCCACACCTTCACCCTTATCAACAAAGCCG
GCAAAGTAACATACGTGAAATTCCATTGGCGCCCAACTTGCGGTGAAAAGAATTTGCT
CGAGGAAGAAGCCATTCGAGTTGGAGGTGAAAACCACAGCCACGCGACTCAAGACCT
TTACGAGTCGATTGCATCGGGTAACTTCCCAGAATGGAAGCTTTACATTCAGACTATGG
ATCCGAATGATGAGGATAAGTTCGATTTCGATCCACTTGATATGACTAAAACATGGCCT
GAAGATATCTTGCCCTTACAACCTGTAGGACGTCTTGTGTTGAACAAGAATGTTGACAA
CTTTTTTACTGAAAATGAGCAACTGGCTTTTGATCCAGCCCATGTTGTTCCTGGGATTTA
TTTCTCTAATGATAAGATGCTTCAAGCGCGTACTTTTGCGTATGCTGATACACATAGATAT
CGTTTGGGTATCAACTACTTGCAGCTTCCTGTTAATGCTCCTAAGTGCCCGCATACTAAT
ATGCATCGTGATGGTGCTATGAACATGGTGCACAGGGATGAAGAGATTGACTATTTCCC
ATCAAGGTTTGATCCTCTTCGTGAAGCTGAAAGATTCCCTATTCCAACAGCAATCATCC
ATGGAAGGCGTGAAAAGCAAATCATTCCAAAGGAAAACAACTTCAAGGAGCCGGGAG
TGAGATACCGATCATGGGACCCAGCAAGGCAGGACAGATTTGTTGGAAGATTTGTGAA
GGCTTTATCAGATCCTCGCCTTACCTATGAGATCCGTAGCATTTGGGTTTCATACTTGAC
TCAGGCTGACAAATCTCTGGGTATGAAAGTGGCAAGTAGACTCAATATAAGACCAACC
ATGTGA
>BvCAT1
ATGGATCCATACAAGTTTCGACCATCAAGTGCGAACAATGCTCCTTATTGTACCACAAA
TTCTGGTGCACCTGTTTGGAATAACAACTCTTCATTAACTGTTAGTCACAGAGGCCCAA
TCCTTTTGGAGGACTACCATCTGGTGGAAAAGCTTGCCAACTTTGATAGAGAGCGTATA
CCAGAACGTGTTGTTCATGCCAGGGGAGCTAGTGCTAAGGGATTTTTTGAAGTTACTC
ATGATATCTCACACCTCACATGTGCTGATTTCATGCGTGCTCCTGGTGTCCAGACACCT
GTAATTGTTCGTTTCTCCACTGTTATTCATGAACGTGGCAGTCCCGAAACCCTTAGGGA
TCCTCGTGGTTTTGCTGTGAAGTTTTACACCAGAGAGGGTAACTTTGATCTTGTGGGGA
ACAATTTCCCTGTGTTTTTCATTCGTGATGGAATGAAATTCCCGGATATGGTGCACTCCC
TCAAGCCCAACCCCAAATCTCACATTCAAGAAAATTGGAGGGTCATGGATTTCTTCTCT
CACCATCCTGAGAGTTTGCACATGTTCACTTTCCTCTTTGATGATATTGGAGTTCCTGCA

AACTACAGGCATATGGAAGGTTCTGGTGTTAACACATATACTCTGATTAACAAGGCTGG
CAAAGTACACTACGTGAAGTTCCACTGGAAGCCAACATGTGGAGTGAAAAGCCTGTT
GGAGGATGAGGCCATAAAAGTTGGAGGAGCTAACCACAGCCATGCTACTCAGGATCTG
TATGACTCAATTGCTGCTGGCAACTATCCTGAATGGAAACTCTTCATCCAGATCATAGA
CCAAGCTGATGAAGACAAGTTTGACTTTGACCCGCTTGATGTTACAAAGACATGGCCT
GAGGACATCTTGCCTCTACTGCCAGTTGGCCGTATGGTCTTGAACAAGAATATCGACAA
CTTTTTTGCAGAAAACGAACAACTGGCATTTTGCCCGGCTATCGTTGTTCCTGGAGTCT
CCTACTCCGATGACAAGCTTCTTCAGACTCGAATCTTCTCCTATTCTGATACCCAAAGA
CACCGTCTTGGTCCCAACTACCTGCAACTTCCAGTTAATGCCCCCAAGTGCTCTCATCA
CAACAATCACTATGATGGTTTCATGAACTTTATGCACAGAGATGAGGAGGTCGACTACT
TCCCATCAAGATATGATCCTGTTCGTCACGCTGAGAGACATCCCATTCCTTCAAACGTC
ATAAATGGAAAGCGTGAAAGGCAAATCATTGCGAAGGAAAACAATTTCAAGCAGCCT
GGAGAGAGATACAGATCAATGGACCCAGCCAGGCAAGAGCGATTCATCTGCAGAGTG
GTTGATGCTTTATCTGATCCTCGCATCACTCATGAGATCCGCAGCATATGGGTTTCATAC
TGGTCTCAGGCTGATAAATCTCTGGGTATGAAAGTAGCAAGTAGACTTAATGTGAGGCC
AACTATGTAA

CDS sequence of SOD, APX and CAT gene family members in *Chenopodium pallidicaule*

>SoCSD3

ATGGGCGGCAGTTTGAAAGGAGTGGCTGTCATCTCCGGCGTCAATAATGACATCAAAG
GCTCTCTTCACTTCATCCAACACCCCACCGGGATAACACATGTAAATGGAAGAATCACT
GGGCTTACACCTGGCCGCCATGGCTTCCATATTCATGCTCTTGGTGATACCACTAATGGT
TGCAACTCCACTGGGCCTCATTTTAATCCAATGAAGAAGGATCATGGAGCACCATCTGA
TGCAGAACGACATGCGGGTGACCTGGGAAACATTGTTGCTGGCAGTGATGGGGTTGCT
GAGATTTCCATTAGTGACTTCCAGATACCACTAAGTGGGCAACACTCAATTCTTGGAAG
GGCTGTTGTGGTGCATGCTGATCCAGATGATCTTGGAAGAGGTGGGCATGAACTCAGC
AAGACAACAGGGAATGCTGGTGCTAGAGTAGGATGCGGTGTAATTGGGTTGCAATCGT
CTGTTTAA

>SoCSD1

ATGGGAAAGGCTGTGGTTGTTCTAAGCAGCAGTGAGGGTGTTTCTGGAACAGTCTACT
TTGCCCAGGAAGGAGATGGTCCAACAACTGTTACTGGAAATGTCTCCGGTCTCAAGCC
TGGGCTCCATGGATTTCATGTTCATGCCCTTGGTGACACAACAAATGGCTGCATGTCAA
CTGGGCCGCACTATAACCCAAATGGAAAAGAGCATGGTGCTCCAGAAGACGATGTTCG
CCATGCTGGTGATCTTGGAAACATCACAGTTGGGGATGATGGTACTGCTACCTTTACCA
TCATTGACAGTCAGATACCTCTTTCTGGACCCAACTCTATTGTGGGGAGGGCCGTTGTT
GTCCATGCTGAACCCGATGATCTTGGAAGGGGTGGACATGAACTCAGCAAGACCACA
GGCAATGCTGGTGGAAGAGTGGCTTGTGGCATTATTGGTCTTCAAGGTTAA

>SoCSD2

ATGCAAGCAGCATTGGCAGCCATGGCCGCACACACCATTCTCGCATCTGCACCTTCTCA
TACAACATTCTCTCTCATCTCTCCCTTCTCTTCCACGCCCACAAACGCCCTTTCATCTTC
CCTTCAATCTTCTTCCTTCAATGGTCTCTCCTTCAAGCTAAGCCCAACCACCCAATCCCT
CTCTCTTTCTACTTCCGCGGCCTCGAAACCACTCACCATTGTTGCTGCTACCAAGAAAG
CTGTTGCTGTTCTTAAGGGTACTTCTAATGTTGAGGGCGTCGTCACTTTGACCCAAGAA
GATGATGGTCCAACTACAGTCAATGTCCGTATTAGTGGACTTGCTCCTGGGAAGCATGG
ATTTCACTTGCATGAGTTCGGAGATACAACAAATGGGTGCATGTCTACAGGTCCACATT

TTAATCCTGATAAGAAGACACATGGTGCTCCGGAAGATGAAGTCCGGCATGCGGGTGA
CCTGGGAAACATTGTTGCTAACACAGATGGAGTGGCTGAGGCTACCATTGTTGACAAT
CAGATTCCATTGACTGGACCAAATTCAGTTGTCGGTAGAGCCCTTGTGGTTCATGAACT
TGAGGATGACCTTGGAAAAGGTGGACACGAACTTAGTCCGACCACTGGGAATGCTGG
TGGAAGATTGGCATGTGGTGTGGTTGGATTAACTCCAGTGTAG
>SoCSD4
ATGACGATTCTGAGGTCAATAGTTACAACAACAACCGCCATAGCAGCCACTGCTCTGC
CTGCAGCTTACGTCGCATCGTCTCTCTCTCACTCTTCTTCATCTCCGACCTCAAAATCTC
TGAACTTCCGCTTTCTCTCTCCTCAATCAGCTTCGAGCTCCATCGCTGGCACTAGATTG
GGGCTTGTAAAGAACTTCAGCTCTCCGCCTTCTGCTGTTCAAATGGAAATTCCCAAGTC
TTCTCCTCAAGAAGATTTGAAGTTGCCGGACTTAATGACAGAGTTCATGGTGGATATGA
AGTGTGAAGGCTGTGTTAGCGCAGTCCAGAGTAAACTAGAGACTCTAGAAGGAGTGA
AGAGTATTGAAGTGGACTTGAGCAATCAAGTTGTTAGGATTCTTGGTTCTTCGCCCGTG
ATAACTATGGAAACTGCTTTGGAGCAAACTGGCCGTAAAGCTCGACTGATTGGTCAAG
GTGTACCTGAAGATTTCCTTGTGTCTGCGGCTGTTGCCGAATTCAAAGGCCCAGATATC
TTCGGAGTAGTTCGCTTTGCTCAGACGCACATGGAATTGACTAGAATCGAGGCCAATAT
TAGTGGGTTGCCATCAGGGAAACATGGATGGTCTATAAACGAGTTTGGTGATCTGACTA
GAGGTCCAGCTAGCACAGGGAAAGTGTACAATCCGCCAGTCCAAGGAAGTGGAAGTG
AGCCGTTGGGTGACCTGGGAACATTAGAAGTCGATGAGAAAGGTGAAGCTTATTACTC
AGGAATCAAGAAGATGATGAGGGTCTTTGATTTAATCGGACGGTCAATTGCATTGTATG
CATCGGAGGACAAGTCTGATCCTGGCCTTGCAGCTGCAGTGATTGCTAGGAGTGCGGG
AGTAGGTGAGAACTATAAGAAGCTTTGCACATGCGACGGAACTACGATTTGGGAGGCA
AAGCCTGATGCTGTCACTAGCAAAGTCTGA
>SoFSD1
ATGTCTGCTGTTGCTGCTCCTTCCGCTATTTCCATTTCTTCCTCATTTCTCACTCATCCAG
TTATGTTTGCAGGGTTTAAAGGGGGGCTCAACTCTACCCGCTTCTCACCATGGAAACTC
AAGGAAACAAGAGTAAGAAGAGCCAGTGCTGATGTTGTTACAGCCAAGTTTGATTTG
AATCCCCCACCTTATCCAATTGATGCCTTGGAGCCTCACATGAGCAAACAGACATTTGA
ATATCATTGGGGGAAGCATCATAGAGCATATGTGGATAACTTGAACAAGCAAATAGTTG
GAACAGAATTAGATGGATTACCTCTCGAAGAAGTAGTACGTATCACATATAATAAAGGG
GACATGCTTCCTGCATTTAATAATGCAGCACAGGCCTGGAACCACGAGTTCTTTTGGGA
GTCCATGAAGCCTGGTGGTGGGGGAAAGCCTTCTGGCGAGCTTTTGGCACAAATTGAA
AAGGATTTTGGTTCTTTTGAAGCATTTACTACTGAATTCAAGACTGCTGCAGCCACACA
ATTTGGTTCTGGCTGGGCTTGGCTTGTTTATAAAGCAAATAAATTGGATGTCGGAAATG
CAGTAAACCCGAAGCCTTCCGAAGATGACAAGAAGCTTGTAGTTGTCAAGAGCCCAA
ATGCTGTGAACCCTCTCGTTTGGGATTATTATCCAATTCTTACTGTTGATGTTTGGGAGC
ATGCGTATTACTTGGACTTCCAGAATCGAAGGCCTGATTACCTCTCGATCTTCATGGAA
AACCTTGTTTCCTGGGATGCTGTCAATGCAAGATATGAGGCAGCCAAGGCTTTTGCTAC
CGAGAGAGAGGAAGAGGCAGCGCAGAATTAA
>SoMSD1
ATGGCGTTTCGAACACTGTTGACCCGAAAAGCCCTAACTTCTGCATTTTCAAAGCAAG
GTACTCTTCTCCAATGTCGATCGTTGCAGACTTTCTCTCTCCCTGATCTTCCGTACGACT
ATGGCGCTCTTGAGCCCGCCATTAGCGGCGAAATCATGCAGATCCATCATCAGAAGCAT
CATCAGACTTACATCACTAACTACAACAAAGCTCTTGAGCAGCTCGATGATGCTATGGC

TAAAGGCGATGCATCTACTGTTGTCAAGCTTCAGAACGCCATTAAATTCAATGGCGGAG
GTCATGTAAATCACTCAATTTTCTGGAAGAACCTTGCTCCAATTAATGAAGGAGGTGGT
GAGCCTCCAAAATCTTCATTGGGTTGGGCGATTGATTCAAATTTCGGTTCTCTAGAAGC
TCTTATCCAGAAAATGAATGCTGAAGGAGCTGCCTTACAAGGTTCTGGGTGGGTGTGG
CTTGGACTAGATACACAGTCGAAGAAGCTTCTTGTTGAAACCACTCCAAATCAGGACC
CACTGGTAACTAAAGGACCCAGCTTGATTCCTATACTCGGTATTGATGTGTGGGAGCAT
GCATACTACCTGCAGTACAAGAATGTGAGACCTGATTATCTGAAGAACATCTGGAAGG
TTATGAACTGGAAATATGCAAGCGAGGTGTATGAGAAAGAGTGCCCCAGCCACTAA
>SoFSD2
ATGCTGCTAACTAGTAAGGGAGTTGGAAAATCAGAAATGGGAAGCTTAACTGCCACTA
ATCCTGTTAGCTCACCTCTTCATGCCAATGGCTTATCTTCCAGGATTAAATGCCCAACTT
TTTTTAATCAGAAAAGAAGAGATGAAGGTTCGAAAAGAAGGCCTGGAGTTGTTGCTTA
CTATGGGCTGAAAAAGCCTCCCTATAACCTTGATGCATTAGAACCATATATGAGCCAGA
AAACTCTGGAGTCACATTGGGGAGTGCAACATAGAAATTACTTGGAAGCTTTGAATAG
ACATTTAAGTAGAAGCGACACACTATATGGCCATACTATGGATGAACTTGTGAAAGTAA
CCTACAATTATGGAAATCCCTTGCCTGAATTCAATGATGCTGCTCAGGTCTGGAATCATG
ACTTTTTCTGGGAATCCATGCAACCTGGAGGCGGGGAGTCTCCAACACTGGGTCTTCT
TCAGCAGATAGAAAAAGATTTGGTTCTTTCCCAAACTTTAAAGAGAAATTTATAGAGG
CAGCCATGACGCTCTTTGGTTCTGGCTGGGTTTGGCTTGTTTTGAAGAGGAAGGAGAA
GCAACTTGCAGTTGTTAAGACATCCAATGCAGTCACTCCTATTCTCTGGGATGATATCCC
CATCATATGCTTGGATTTGTGGGAGCATGCATACTACTTGGATTATAAGAATGATAGAGC
GGAATATGTAAATGTTTTCATGAACCACCTTGTATCATGGCATGCAGCAACAGCACGCC
TTGCTCGGGCACAGGCTTTTGTGAATCTTGGAGAACCTAAAATTCCAGTTGCTTAA
>SoAPX9a
ATGCGAAGAACGAAAAAACAGTTGGTGAATGCAGAATATTTGAAGGAAATCGAGAGA
ACACGAAGAGATCTCCGTGCTCTCATCTCTACCAAGCAATGTGCTCCTATCATGCTTCG
CTTAGCATGGCACGATGCGGGTACATATGATGCAAAGACAAATTCAGGGGGTCCAAAT
GGTTCAATTAGGAACGAGCACGAGTTGAGATACAAAGCCAACAGTGGCCTCAAGATT
GCTCTTGAACTTTGTGAAGAGGTTAAGGCTAATCAACCAAAGATCACATATGTGGATCT
TTATCAGCTTGCTGGGGTTGTTGCGGTTGAAGTCACAGGAGGGCCAACCATCGACTTT
GTTCCAGGAAGAATGGATTGCGCAGATTCTCCTGATGAAGGACGCCTTCCTGATGCAA
ATCAAGGTGCCGGACATTTGAGAGACATCTTTTACAGAATGGGGCTTTCTGACAAGGA
TATTGTCGCGCTTTCAGGAGGCCACACTCTGGTATATTTATCACCATTTGTTGGGACATA
A
>SoAPX10
ATGGCAGCAACAACAAAGAGCATTGTAGATGTTGAATATCTGAAACAAATTGAGGGAG
CTCGTAAAGACCTTCTTGATCTTCTTCAGAAGAACAAGAAGTTTTCTCCCATTGTGCTT
CGTTTAGCATTTCATGAAGCGGGTACTTATGACGCCAAAACTAAAACAGGAGGTCCAA
ATGGTTCGATTAGGAATGAGTTGAACAACCCTGCCAACAACGGTATCAAAGTTGGAGT
TGATTTTTGCGAACAAGTGAAGGGTAAACACCCCAAAGTTACTTATGCTGACTTTTACC
AGCTTGCTGGAGTAATTGCTGTTGAAGTTACTGGAGGCCCTAAGATCCCATTTTTCCCG
GGTCGACCCGATGCGCCAAGTCAACAAGACAGTGGAGCTCTTCCAAACCCCAATGGA
GATGCCAAGCATCTTAGGGATATATTTTATCGTATGGGGTTGAACGATAGGGATATCGTT
GCTCTATCAGGATCTCACACACTGGGTCGAGCAAATCAGGATAGATCAGGTTTTGATGG

TCCTTTCACCGAGGATCCTTTCAAATTCGATAATTCGTATTTTGTAAACTTGAGGAAGA
GTGACACCCCTGGACTGGTGAAATTCCCCACTGACAAAGTTCTTGTGGAAGATCCTGC
TTTCAGTCGCTATGTTCAACTCTATGCTAAGGATGAAAAGACTTTCTTCGCACACTATG
CGGAATCACACAAGAAGATGTCGGAGCTCGGGTTTCGCCCACCAAGCCCGGTCGTGA
AAGCTTGA
>SoAPX3c
ATGTCACACGCACAGTTGAAAGCAGAGCAGATTGTGGATGAAGAGTACTTGAAACAA
ATTGAAGGAGCTCGTAGACATCTTCATTGTCTTCTGCAAAACGATAAGCAGTTTGCTCC
AATCTTGCTTCGCTTAGCATTTCATGATGCGGGTACTTATGACGCGAAAACAAAGACAG
GAGGTCCAAATGGTTCGATTAGGAATGAGTTGAGCAATCCTCCCAACAATGGTGTTAA
AAATGCTATTGATTTTTGCGAACAAGTGAAGGTCAAGTACCCCAAGGTTACTTATGCTG
ACCTTTACCAGCTAGCTGGTATGGTTGCAGTTGAAGTTACTGGAGGTCCGATTATCCCT
TTTGTCCCCGGCCGTCCGGATGCCCCGAATGAACAAGACAGTGGAGCTCTTCCAAATC
CCAATGGAGGATCCAATCATCTGAAAGATATATTTACCGAATGGGCTTGAACAATAGG
GATATTGTTGTTTTATCTGGAGCTCACACACTGGGTCGAGCACACAAGGATAGGTCAGG
TTTTGATGGTCCTTTCACCGAACAACCTTTGAAGTTCGACAATTCCTATTATGTAAACTT
GAGGAAGCCTGACATTCCTGGACTGGTGAAATTCCCCTCGGATAAAGTTCTCGCTGAG
GATCCTGATTTCCGTCGCTATGTTCAACTGTATGCTAAGGATGAGAAGGTGTTCTTCGC
ACACTATGCTGAGTCACACAAGAAGATGTCGGAGCTCGGGTTTCACCCACGTGCACCT
TGCTCTTGCTCTGCTATGAAAGCTTGA
>SoAPX7
ATGACTGATCGTCTAGCAATGGCATCCTTCACTACCACCACCGCCGCCGCTGCATCTCG
TCTGCTTCCTTCTTCTTCCTCCTCCATCTCTCGACTTTCTCTCTCCTCTTCTTCCTCCTCC
TCCTCCTCACTCAAATGTCTCCGATCATCTCCACTCGTCTCTCACCTCTTCCTTCGACAG
AGAGGAGGTTCAGCTTATGTGACGAAGACGAGGTTTAGCACGAAATGCTACGCTTCTG
ATCCTGCGCAGCTGAAGAATGCTAGGGAAGATATTAAAGAGCTTCTTCAATCTAAGTTC
TGTCATCCTATTATGGTTCGCTTAGGTTGGCACGATGCCGGTACTTATAATAAGGACATT
AAAGAATGGCCACAAAGAGGTGGAGCCAATGGCAGTCTGAGGTTTGATGTTGAGCTC
AAGCATGGAGCTAATGCAGGTCTTGTTAATGCCCTGAAACTTCTACAGCCCATAAAAG
ACAAGTACTCTGGAGTTACATATGCAGATCTATTCCAGCTGGCTAGTGCTACTGCAATA
GAGGAGGCTGGTGGTCCAACAATACCCATGAAGTATGGAAGAGTGGATGCCACAGGG
CCGGAGCAGTGCCCAGAAGAAGGAAGGCTTCCTGATGCTGGACCTCCTTCACCTGCTC
AACATCTACGTGATGTTTTCTACAGAATGGGTCTTGATGATAAGGATATAGTAGCATTAT
CTGGAGCACATACGTTGGGAAGGTCTAGACCTGAACGCAGTGGTTGGGGCAAGCCAG
AGACTAAATACACGAAAGATGGACCTGGAGCTCCAGGAGGGCAGTCATGGACTGCGG
AGTGGTTGAAGTTTGATAATTCCTATTTCAAGGACATCAAAGAAAAGAGAGATGCAGA
TTTGCTTGTTTTGCCAACTGATGCTGCTCTTTTCGAAGATCCGTCTTTCAAGGTATATGC
AGAGAAATATGCAGCTGACCAAGAAGCATTTTTCAAGGATTACGCTGAAGCCCATGCC
AAACTCAGCAACCAAGGAGCCAAATTTGACCCTGCTGAGGGTATCACTCTTAATGGAA
CCCCTGCCGGAGCAGCTCCAGAGAAGTTTGTAGCAGCCAAGTACTCATCTAACAAGAG
ATCAGAGCTTTCGGATTCTATGAAGGAAAAGATTCGCGCTGAATATGAAGGTTTTGGAG
GTAGCCCTAATAAGCCTCTACCAACAAACTACTTCCTAAACATTATGATTGTGATTGGAG
TTTTGGCAGTTCTATCATATCTTGCGGGAAATTGA
>SoAPX10a

ATGGCAGCAACAACAAAGAGCATTGTAGATGTTGAATATCTGAAACAGATTGAGGGTG
CTCGTAAAGACCTTCTTGATCTTCTCCAGAAGAACAAGAAGTTTTCTCCCATTGTCCTT
CGTTTAGCATTTCATGAAGCAGGTACTTATGACGCTAAAACAAAAACAGGAGGTCCAA
ATGGTTCGATTAGAAATGAGTTGAACAACCCTGCTAACAACGGTATCAAAGTTGGTATT
GATTTTTGTGAGCAAGTGAAGGGTAAATACCCCAAGGTTACTTATGCTGACTTTTACCA
GCTTGCTGGAGTGCTCGCTGTTGAAGTTACTGGAGGACCTAAGATCCCGTTTTTCCCG
GGTCGACCTGATGCCCCAAATAACCAAGACAGTGGAGCTCTTCCAAACCCCAATGGAG
ATGCCAAGCATCTCAAGGATATATTTTACCGAATGGGCTTGAACGATAGGGATATCGTTG
TTCTATCAGGGTCTCACACACTGGGTCGAGCAAATCAGGATAGGTCAGGATTTGATGG
GCCATTCACAGAGGATCCTTTCAAATTCGATAATTCCTACTATGTAAACTTGAGGAAGA
GTGATACCCCTGGACTGGTGAAATTCCCGACTGACAAAGTTCTGGTGGAGGATCCTTC
TTTCAGTCGCTATGTTCAACTCTATGCTAAGGATGAGAAGGCTTTCTTCGCACACTATG
CTGAATCACACAAGAAGATGTCGGAGCTCGGGTTTCGCCCACCTACCCCTGCAGTCAA
AGCTTGA
>SoAPX9
ATGATGGGAAGAACGAAAAAACAGTTGGTGAATGCAGAATATTTGAAGGAAATTGAG
AGAACACGAAGAGATCTCTGTGATCTCATCTCTACCAAGCAATGTGCTCCTATCATGCT
TCGCTTAGCATGGCACGATGCAGGCACATATGATGCAAAGACAAATTCAGGGGGTCCA
AATGGTTCTATTAGGAACGAGCACGAGTTGAGACACAAAGCCAACAGTGGCCTCAAG
ATTGCTGTTGAACTTTGTGAAGAGGTTAAGGCTAATCACCCAAAGATCACTTATGCGGA
CCTTTATCAGCTTGCCGGGGTTGTTGCGGTTGAAGTCACAGGAGGGCCAACCATCCAC
TTTGTTCCAGGAAGAATGGATTGCGCAGATTCTCCTGATGAAGGACGCCTTCCTGATGC
AAATCGAGGTGCTGAACATTTGAGAGAAATCTTTTACAGAATGGGGCTTTCTGACAAG
GATATTGTCGCGCTTTCAGGAGGCCACACTCTGGGTAAGGCACATAAGGACAGATCAG
GTTTCAAAGGCCCTTGGACCAAAGAGCCATTCAAATTCGACAATTCCTATTTTATTGAG
TTGCTGAAAGGTGAACAATGGGGGTTACTGAAGCTTCCAACTGATAAGGTTCTCATAG
AGGATCCAAGATTCCGTCCCTTCGTTGAGCTTTATGCCAAGGATGAAGAGGCGTTCTTC
AAAGATTATGCAGAATCACACAAGAAGCTCTCAGAGTTGGGGTTCACTCCACCCACCA
CCAACAGTTTGGGGATGATTACTAAAGCTACACTTGGAGTTGTGGTCACTCTTACTGTA
ATTATCTTAAGCTACCTGTATGAAGTTAATAAACAAGGCTAA
>SoAPX1
ATGGGAAAGAGCTACCCAACTGTCAGTGAGAACTACCAGAAATCTATTGAAAAGGCCA
GGAGAAAGCTCAGGGGTTTGATCGCAGAGAAGCAATGTGCTCCTCTTATGCTTCGTCT
TGCATGGCACTCTGCTGGTACCTTTGATTGTACTTCAAAAACTGGAGGTCCCTTTGGTA
CAATGAAGCACCAGGCAGAGCTGGCTCATGGGGCTAACAATGGGCTTGTTATTGCTGT
TAGGCTGTTGGAACCCATCAAGGAACAATTCCCCGAAATTACTTATGCTGACTTTTACC
AGCTGGCTGGAGTTGTGGCCGTTGAAGTTACTGGAGGACCTGAAGTTCCCTTCCACCC
AGGCAGAGAGGACAAGCCAGAGCCACCCCAGGAAGGACGTCTCCCTGATGCCACCA
AGGGTTGTGACCATTTGAGAGATGTCTTCATCAAGCAAATGGGTCTTACTGACCAGGA
CATCGTTGCTCTATCTGGAGGCCACACCTTGGGGAGATGCCACAAGGACCGCTCTGGT
TTTGAAGGTGCTTGGACTACCAACCCTTTGGTCTTCGACAACACCTACTTCAAGGAGC
TCCTGAGTGGTGAGAAGGAAGGTCTCTTGCAGCTGCCATCTGACAAGGCTCTTCTCTC
TGACCCTGTCTTCCGCCCACTGGTTGAGAAATATGCAGCTGATGAAGATGCATTCTTTG
CCGACTATGCTGAGGCGCACTTGAAACTTTCTGAACTCGGATTTGCTGATGCTTAA

>SoAPX6
ATGGATCAGATAAATGTTTTGGACCGTTCGCTGAAGATGACCACCCCCGGCGGCGAAT
CAACAGTAATATGCCGCTCTTTCGCCACCCAATCATCGACATCCTCCCTCAACAAATTC
AAATTTCCCGCCAAATATCCACCGTCAACCGTCCATTTTGGCGCCAAAGCAGCTCCGTT
TCTCACTCCCCCCTTCGCACTTCCACCCACTTCAGATAATTACAGCAATGTTGGTTTGT
GTGGAAGGAGAGGACTCATTTTTCTTTCTGTCACTCTACCTTTTCTTTTTCCTTCACAA
GAGTATGTCAAAGATTTCAATGTTGAAGCAGAAGAGCTCAGAGATCCTGATTACGTACT
TATAAAGGATGAGATAAGGAAGGTTTTGTCTAAAGGAAAGGCTGCTGGTGTACTTCGT
CTAGTGTTTCATGATGCAGGAACCTTTGAACTCAGGGACAATTCAGGAGGTATGAATG
GCTCTATAACATATGAACTTGAGAGGCCTGAAAATAAAGGGCTCAAAAAGTCTCTGAA
GATTATTGAGAAAGCCAAAAATGTGGTGGATGAGAGACAGTCAGTATCCTGGGCAGAC
ATGATTGCTGTAGCAGGAGCTGAGGCAGTTTCGATATGTGGAGGCCCAGATATTCCAGT
TCGGTTGGGGAGACTTGATTCACTGACTCCTGATCCTGAAGGGAAACTACCTGAAGAG
TCACTGGATGCACCAAGCTTAAAGAAGTGTTTCCAAACAAAAGGCTTTTCCACACAAG
AACTTGTTGCACTGTCTGGGGCTCATACTCTTGGAAGTAAAGGTTTTGGAAGCCCCATC
GAATTCGACAATTCATATTACAAAATACTCCTAGAGAGTCCGCTCTCGTCAGCTGGAAT
GTCTAGTATGGTTGGACTTCCTTCAGATCGTGCATTAGTCGAGGATGATGAATGCTTAA
GATGGATTACAAAGTACGCGAATGACCAGGCTTTATTCTTTGAGGATTTTAAGAATGTC
TATATCAAGTTAGTCAATTCTGGTGCAAGGTGGAAATCTTCATGA
>SoAPX1a
ATGGGAAAGAGCTACCCAACTGTCAGTGAGGACTACCAGAAATCTATTGAAAAGGCCA
GGAGAAAGCTCAAGGGTTTGATCGCACAGAAGCAATGTGCTCCTCTTATGCTTCGTCT
TGTATGGCACTCTGCTGGTACCTTTGATTGTACTTCAAAGACTGGAGGTCCCTTTGGTA
CAATGAAGCACCAGGCAAAGCTGGCTCATGGGGCTGACAATGGGCTTGATATTGTTGT
TAGGCTGTTGGAACCCATCAAGGAAAAATTTCCCGAAATTTCTTATGCTGACTTTTACC
AGCTGGCTGGAGTTGTGGCTGTTGAAGTTACTAGAGGACCTGAAGTTCCCTTCCACCC
AAGCAGAGAGGACAAGCCAGGGCCACCCCAGGAAGGACGTCTCCCTGATGCCACTAA
GGGTTGTGACCATTTAAGAGATGTCTTCATCTAG
>SoAPX9b
ATGCGAAGAACGAAAAAACAGTTGGTGAATGCAGAATATTTAAAGGAAATCGAGAGA
ACACGAAGAGATCTCCGTGCTCTCATCTCTACCAAGCAATGTGCTCCTATCATGCTTCG
CTTAGCATGGCACGATGCGGGTACATATGATGCAAAGACAAATTCAGGGGGTCCAAAT
GGTTCAATTAGGAACGAGCACGAGTTGAGATACAAAGCCAACAGTGGCCTCAAGATT
GCTCTTGAACTTTGTGAAGAGGTTAAGGCTAATCAACCAAAGATCACATATGTGGATCT
TTATCAGCTTGCTGGGGTTGTTGCGGTTGAAGTCACAGGAGGGCCAACCATCGACTTT
GTTCCAGGAAGAATGGATTGCGCAGATTCTCCTGATGAAGGACGCCTTCCTGATGCAA
ATCAAGGTGCCGGACATTTGAGAGACATCTTTTACAGAATGGGGCTTTCTGACAAGGA
TATTGTCGCGCTTTCAGGAGGCCACACTCTGGTATATTTATCACCATTTGTTGGGACATA
A
>SoAPX10b
ATGGCGATGCCGGTCGTCAACACAGAATACCTCAAGGAAATCGACAAAGCTCGTCGCG
ATCTTCGCGCTCTCATCTCTAATCGCAATTGCGCTCCTCTCATGCTCCGCCTCGCGTGGC
ATGATGCAGGGACTTATTGCGCGAAGACGAAGACCGGCGGCCCTAATGCTTCAATTCG
GAACGAGGAAGAGTGCGCTCATGGTGCCAATAATGGTTTGAAGAAAGCTATTGATTGG

TGTGAGGAGGTGAAGTCTAAACACCCAAAGATTACATATGCAGACCTCTATCAGCTTG
CTGGTGTTGTTGCAGTTGAGGTCACTGGAGGACCTACTGTTGACTTTGTTCCTGGGAG
AAAGGACTCAAATGCGTGTCCCAAGGAAGGGCGACTTCCAGATGCTAAACAAGGTGC
ACCACATCTGAGGGACATATTTTACAGGATGGGTCTCACTGACAAAGACATCGTAGCA
CTGTCAGGGGGTCATACCCTGGGAAGGGCACATCCAGAGAGATCAGGCTTTGATGGCC
CATGGACCCAGGAGCCACTCAAGTTTGATAATTCCTATTTTGTGGAACTCTTGAAAGGA
GAATCAGAAGGACTGTTGCAACTTCCTACTGATAAAACTTTGGTGGAGGACCCTGCAT
TTCGCCCTTTCGTGGAACTGTATGCTAAGGATGAGGATGTCTTCTTCCGTGACTATGCT
GTCTCACACAAGAAACTTTCGGAATTAGGATTTACTCCAAGCGGAGGCAAGTCAAGCT
GCAAAGACAGCACTATACTGGCTCAGAGTGCAGTCGGAGTTTTAGTTACTGCAGCAGT
GGTCATTTGTAGCTACATCTATGAAGTCCGCAAACGCTCAAAGTGA
>SoCAT
ATGGATCCTTACAAGTTTCGGCCATCAAGTGCGAACAACTCACCATACTGCACCACCA
ATTCTGGTGCGCCTGTTTGGAATAACAACTCTTCTTTAACTGTTGGTAACAGAGGCCCA
ATTCTTTTGGAAGACTATCATCTGGTGGAAAAGCTTGCCAACTTTGACAGAGAGCGTAT
ACCAGAACGTGTTGTTCATGCCAGGGGAGCGAGTGCTAAGGGATTTTTTGAAGTTACT
CATGATATTTCACAACTCACGTGTGCTGATTTCCTGCGCGCCCCTGGCGTCCAGACACC
TGTAATTGTTCGGTTCTCCACTGTTATTCATGAACGTGGCAGTCCGGAAACCATCAGGG
ACCCTCGTGGTTTTGCTGTGAAGTTCTACACCAGAGAGGGTAACTTCGATCTGGTAGG
AAACAATTTCCCTGTGTTTTTCATTCGAGATGGTATGAAATTCCCTGATATGGTGCATGC
CCTCAAGCCCAACCCTAAATCTCACATTCAGGAGAATTGGAGGGTCATGGATTTCTTCT
CGCATCATCCCGAGAGTTTACACATGTTTACTTTCCTCTTTGATGATATTGGAGTTCCTG
CAAACTATAGGCACATGGAAGGCTCTGGTGTTAACACATATACTCTGGTCAACAAGGCT
GGCAAAGCATATTATGTGAAGTTCCACTGGAAGCCAACATGTGGAGTGAAGTGCCTGT
TGGAGGATGAGTGCGTCAAAATTGGTGGAGCTAACCACAGCCATGCTACTCAGGATCT
TTATGACTCGATTGCAGCTGGAAACTATCCCGAATGGAAGCTGTTCATCCAGACAATAG
ACCCAGCTGATGAAGACAAATTTGACTTTGACCCCTTAGATGTTACAAAGACTTGGCC
TGAGGACATTTTGCCTCTTCAGCCAGTTGGCCGTATGGTCTTGAACAAGAACATTGAC
AACTTCTTTGCCGAGAACGAGCAGCTGGCATTTTGCCCTGCTATCGTTGTTCCTGGGGT
CCACTACTCAGATGACAAGCTGCTTCAGACTCGAATTTTCTCATATGCTGATACCCAAA
GACACCGTCTTGGACCTAACTACCTGCAACTTCCAGTTAATGCTCCCAAGTGTGCTCAT
CACAACAACCACTATGATGGTTCCATGAACTTTATGCACAGAGATGAGGAGGTTGACT
ACTTCCCATCAAGATATGATCCTGTTCGTCACGCTGAGAGATTTCCCATCCCTACAAAC
ATCTTGAATGGAAAGCGTGAAAGGCAAATCATTGTAAAGGAAAACAACTTCAAGCAG
CCTGGAGAGAGATACAGATCCATGGACCCAGCCAGGCAAGAGAGATTTATATGCAGAG
TGGTTGATGCTCTCTCTGATCCTCGCATCACTCATGAGATAAGAAGCATTTGGGTTTCAT
ACTGGTCTCAGGCTGACAAATCTCTGGGTATGAAAGTCGCAAGTCGACTTAATGTGAG
ACCAACCATGTAA
>SoCAT2
ATGGATCCATCTAAGTACCGCCCGTCAAGTGCTTATAACTCCCCGTACTTAACCAACAA
TAATGGTACACCTATTTGGAACGATACTTCTGCCTTAACTGTTGGCCCTAGAGGTCCAAT
TCTGCTGGAGGACTATCACCTGATTGAAAAGATAGCAACCTGGACACATGAGCGGATA
CCTGAACGTGTTGTACATGCTAGGGGAGCAAGTGCCAAGGGTTTTTTTGAAGTTACTC
ATGATATCACTAACCTCACTTGTGCTGATTTCCTGCGCGCCCCTGGCGTCCAGACGCCT

TGTATTGTCCGATTTTCCACTGTTATTCACGAACGTGGTAGTCCTGAAACACTCAGGGA
TCCTCGTGGTTTCGCCATCAAGTTCTACACTAGAGAGGGTAACTTTGATATTGTGGGGA
ACAACTTCCCAGTGTTTTTCACCCGTGATGCAATGCAGTTCTTCGATCTGATCCGAGCA
TTTAAGCCTAACCCAAAATCCCATATCCAAGAAAGCTGGAGGTACATGGATTTCTGCTC
TTACCTCCCAGAGAGTCTAAACACCTTCACTTTCTTCTTCGACGATGTGGGAATTCCTA
TCAATTACAGACACATGAATGGTTCTGGAGTCCACACCTTCACTCTAATCAACAAGGC
AGGAAAAGTAACATATGTCAAGTTCCATTGGCGTCCAACAGTTGGGGAAAAGAACTTG
CTCGAAGAAGAAGCCATTAAGGTAGGTGGCGCGAACCACAGTCACGCCACACAGGAC
TTGTATGAGTCTATTGCAAATGGGAATTTCCCAGAATGGAAACTCTACATCCAAACCAT
GGACCCTGCTGATGAAGACAAGTTTGATTTTGATCCACTTGATACCACTAAGACATGGC
CTGAAGATATCTTGCCATTACAGCCTGTAGGACGTCTTGTGTTGAACAAGAATGTCGAT
AATTTCTTCACTGAAAATGAACAACTGGCTTTTGATCCTGCACATATTGTTCCTGGGATT
TATTTCTCCAATGATAAGATGCTTCAAGCTCGTACTTTTGCATATGCTGATACACATAGAT
ACCGTTTGGGTGTCAACTATTTGCAACTTCCTGTTAATGCTCCTAAGTGTCCTCATACCA
ATATGCACCGCGATGGTGCTATGACCATGATACACAGGGATGAAGAGATTGATTACTTC
CCATCAAGATTTGATCCTCTTAGGGAAGCTGAGAGACATCCAATTCCAACAGCCATCAT
ACATGGAAGACGTGAAAAGCAAATCATTCCAAAGGAGAACAACTTCAAGGAGCCAGG
AGAGAGATACAGATCATGGGACCCAGCAAGGCAAGAGAGATTCATTGGGAGATTTGTT
CAAGCTCTATCCGATCCACGACTCACTTATGAGATCCGCAGCATTTGGGTGTCATACTT
GTCTCAGGCGGACAAATCTCTGGGTATGAAAGTAGCAAGTAGGCTCAACGTGAGACCA
ACCATGTAA

CDS sequence of SOD, APX and CAT gene family members in *Chenopodium pallidicaule*

>CpCSD1

ATGGGCAAAGCCGTGGTTGTTCTAAGCAGCAGTGAGGGTGTTGCTGGTACTGTTTACT
TCACCCAAGAAGGAGATGGTCCAACAACTGTAACTGGAAATGTCTCGGGTCTTAAGCC
TGGGCTCCATGGATTTCATGTTCATGCTCTTGGTGACACCACAAATGGCTGCATGTCAA
CTGGACCACACTTTAACCCAAATGGAAAAGAGCATGGAGCTCCCGAAGACGAAGTTC
GCCACGCTGGTGATCTTGGAAACATCACAGTTGGGGATGATGGTACCGCTACCTTTACC
ATCATTGATAACCAGATACCTCTTTCTGGTCCCAATTCTATTGTGGGGAGGGCTGTTGTT
GTCCATGCTGATCCCGATGATCTTGGAAGGGGTGGACATGAACTCAGCAAGACCACAG
GCAATGCTGGTGGAAGAATCGCTTGTGGTATTATTGGTCTTCAAGGTTAA

>CpCSD3

ATGAGCACAGTGAAAGGAGTGGCAGTCATCTCCGGCGCCAATAACACCATCAACGGCT
CTCTCCACTTCATCCAAACCTCCACAGGGATAACTCATGTTAAAGGAAGAATTACAGGC
CTCACTCCTGGTCTTCATGGCTTCCATATTCATTCTCTTGGTGATACCACTAATGGTTGC
AACTCCACTGGGCCTCATTTTAATCCGTTAAAGAAGGATCATGGAGCTCCATTTGATTC
AGAGAGACATGCGGGTGATCTGGGAAACATTGTTGCAAACAGGGATGGGGTTGCTGA
GGTTTCAATTATTGATTCCCAGATACCACTTAGTGGGCAACACTCAATTCTTGGGAGGG
CTGTTGTGGTGCATGCTGATCCAGATGATCTAGGAAGAGGTGGACATGAACTCAGCAA
AACAACAGGGAATGCCGGTGCTAGAGTAGGATGCGGTGTAATTGGGCTGCAATCGTCT
GTATAG

>CpCSD2

ATGGCCGCACACACCATCCTTGCATCTGCACCTTCTCATCCCACATTCTCTCTCCTCTCT
CCTTTCTCTTCCTCACCCACTACTTCTTCTCCTACCACCCTTTCATCTTCTCTTAAATCTT

CTTCCTTCAATGGTGTCTCTTTCAAGCTTTCTTCCCACACTCAATCCCTCTCTCTTTCCG
CTTCCGCTTCCGCCACCTTCAAACCTCTCACCATTGTTGCTGCTACCAAAAAAGCTGTT
GCTGTTCTAAAGGGTACTTCCAATGTTGAGGGTGTTGTTACTTTGACCCAGGAAGATGA
TGGTCCAACTACAGTCAATGTTCGTATTACTGGACTTACTCCGGGGAAGCATGGATTTC
ACTTGCATGAGTTTGGAGATACAACAAATGGGTGCATGTCTACAGGTCCACATTTTAAT
CCTAATAAAATGACACATGGTGCTCCGGAAGATGAAGTCCGGCATGCGGGTGACCTGG
GAAACATTGTTGCTAACACAGATGGAGTGGCTGAGGCTACCATTGTTGATAATCAGATT
CCATTGACTGGACCAAATTCAGTTGTCGGTAGAGCCCTTGTGGTACATGAACTAGAGG
ATGACCTTGGAAAGGGTGGACATGAACTTAGTCTGACCACTGGGAATGCTGGTGGAAG
ATTGGCTTGTGTTCTATTTGGTTTGTAA
>CpCSD4
ATGACGATTCTGAGGTCAATAGTCACAACAACAACCGCCATAGCAGCCACAGCTCTGC
CTGCTGCGTACGTCGCATCTTCTCTCTCCTCTTCATCTTCTTCACCATCTTCATCTTCTTC
TCCCTTCTCGAAGTCTCTGGACTTCCGCTTTCTCTCTCCTCAAGCACCATCGACTTCGG
GGGTTGCTGCTAGATTCGGCCTTATCAAGAGTTTCAGCTCTCCGCCTTCTGCTGTTCAA
ATGGAGAATCCTAAGTCTTCTTCTCATGAAGATTTGAAGTTGCCTGACTTAATGACAGA
ATTTATGGTGGATATGAAGTGTGAAGGTTGTGTTAGTGCTGTCAAGAGTAAACTAGAGA
CTCTATATGGAGTTAAGAGTGTTGAAGTAGACTTGAGCAATCAAGTTGTTAGGATTCTT
GGTTCTTCACCTGTGGTATCTATGGAAAATGCTTTGGAGCAAACTGGACGTAAAGCAC
GATTGATTGGTCAAGGAGTACCAGAAGTTGCCGAGTTTAAAGGTCCAGATATTTTTGGT
GTGGTTCGTTTTGCCCAGACACACATGGAGTTGGCCAGAATCGAGGCCAATATCAGTG
GGTTGCCACCTGGGAAACATGGCTGGTCTATAAATGAGTTTGGTGATCTGACTAAAGGT
CCAGTCAGCACGGGGAAAGTGTACAAGCCACCAATCCAAGGAAGTGAAAGTGAGCCA
CTGGGTGACCTGGGAACATTAGAAGTTGATGAGAAAGGCGAAGCTTATTATTCAGGAA
TCAAGAAGATGCTGAGGGTTGTTGATTTAATTGGACGGTCCATTGTCTTGTACGCATCT
GAGGACAAGACTGATCCTGGCCTTACAGCTGCAGTGATTGCTAGGAGTGCGGGAGTAG
GTGAGAACTATAAGAAGCTTTGTACATGCGATGGAACCACGATCTGGGAAGCAAAGCC
TGATATTGTCACTAGCAAAGTCTGA
>CpMSD1
ATGGCGCTTCGAACTCTGATGACCAGGAAAACCCTAGCTTCTGCATCTTCAAACCAAG
GTCTTCTTCATCAATGTCGATCGTTGCAGACTTTCTCTCTCCCTGACCTTCCGTACGACT
ATGGCGCTCTTGAGCCTGCCATTAGCGCCGAAATCATGCAGCTTCACCATCAGAAGCAT
CACCAGACTTACATCACTAACTATAACAAAGCTCTTGAGCAGCTCGATGATGCTATTGC
TAAAGGCGATGCTTCTAGTGTTGTTAAGCTTCAAAGCGCCATTAAATTCAATGGCGGAG
GTCATGTGAATCACTCAATTTTCTGGAAGAATCTTGCTCCAATTAGTGAAGGAGGTGGT
GAGCCTCCAAAAGCTTCACTGGGTTGGGCAATTGATTCAAACTTTGGCTCTCTGGAAG
CTCTTATCCAGAAAATGAATGCGGAAGGAGCTGCCTTACAAGGTTCTGGATGGGTGTG
GCTTGGACTTGACACACAATCCAAGAAGCTTCTTGTTGAAACCACTCCAAATCAGGAC
CCTCTGGTAACTAAAGGACCAAGTTTGGTTCCTATACTTGGTATAGACGTATGGGAGCA
TGCGTACTACCTGCAGTACAAGAATGTGAGACCTGATTATCTGAAGAACATCTGGAAG
ATTATGAACTGGAAACATGCAAGCGAGGTGTATGAAAAAGAATGCCCCAGCCACTAA
>CpFSD2
ATGGGAAGTTTTACTGCAATTACGCCTGTTAGCTCATCTCTTCATGCCAATGACTTGTCG
TTCAGGATTAAAAATTTTATCCTTAATTATCAGCAGAAAAGAAGACATGAAGGACCGAG

AAGAAAGTCCGGTGTTGTTGCATATTATGGGCTTAAAAAGCCTCCTTATAACCTTGATG
CACTAGAACCATATATGAGCCAGAAAACTCTGGAGTCACATTGGGGAGAGCATCATAA
AGGTTACCTGGATGCTTTGAATAGACATTTGAGTAAGAGTGATATACTATATGGCTATAC
TATGGATGAACTTGTGAAAGTAACGTACAATTATGGAAACCCCTTACCCGAATTCAATG
ATGCTGCACAGGTCTGGAATCACGACTTTTTCTGGGAATCCATGCAACCTGGAGGTGG
GGAGTCTCCAACACTCGGTCTACTTCAGCAGATTGAAAAAGATTTTGGTTCTTTTCCAA
ACTTTAAAGAGAAATTTATAGAAGCAGCTATGACACTCTTTGGTTCTGGTTGGGTTTGG
CTGGTTTTGAAGAAGAAGGAGAAGCAACTTGCAGTGGTTAAGACATCAAATGCAGTC
ACTCCTATTGTCTGGGATGATATCCCCATCTTATGCTTGGATGTGTGGGAGCATGCTTAC
TACTTGGATTATAAGAATGATAAAGCGAAGTATGTAAATGTGTTCATGGACCACCTTGTA
TCATGGCATGCTGCAACAGCACGCCTTGCTCGGGCACAGGCCTTTGTGAATCTTGGAG
AACCTAAAATTCCAGTTGCTTAA
>CpFSD1
ATGTCCGCAATTGCTGCAACTTCCTCAATTTCACCATCTTCCTCAATTTTCACTCATGAA
GTGATTTTTGCAGGGTTTAAAGGGGGCCTAAACACTATCCGCTTCTCACCTTGGAAGCT
TAAGGAAACAAGAGTAAGAAGAGCCGGTGCTGGTGTTGTTACAGCCAAGTTTGACTT
GAAGCCCCCACCTTATCCATTTGATGCCTTGGAGCCGCATATGAGCAAACAGACGTTTG
AGTATCATTGGGGGAAGCATCATAGAGCATATGTGGATAACTTAAACAAGCAAATAGTT
GGAACAGAATTAGATGGATTGCCACTTGAAGAAGTCGTACGAATCACATATAACAAAG
GGGACATGCTTCCTTCTTTTAATAATGCAGCACAGGCCTGGAACCATGAAATCTTTTGG
GAGTCCATGAAGCCTGGTGGTGGGGGAAAGCCTTCTGGCGAGCTTTTGGCACAGATTG
AAAAGGATTTTGGTTCATTTGAAGCATTTACTAATGAATTCAAGACTGCTGCTGCCACA
CAATTTGGTTCTGGCTGGGCTTGGCTTGTTTACAAAGCAAATAAATTGGATGTGGGAAA
TGCAGTAAACCCAAAACCATCTGAAGATGACAAGAAGCTTGTGGTTGTTAAGAGCCCA
AATGCTGTGAACCCTCTTGTTTGGGATTATTATCCGCTTCTTACTATCGATGTTTGGGAG
CATGCATATTACTTGGACTTCCAGAATCGAAGGCCTGATTACATCTCAATTTTCATGGAA
AATCTTGTTTCCTGGGATGCTGTCAATGCAAGATATGAGGCTGCTAAGTCTCTTGCTAC
GGAGAGAGAGAAAGAAGCAGTGCAGAATTAA
>CpAPX10a
ATGGCAGCCACAACAAAGAGCGTAGTTGATGTAGATTATCTGAAACAAATTGAGGGTG
CTCGTAAAGATCTTCTTGATCTTCTTCAGAAGAACAAGAATTTTTCACCCATTGTGCTT
CGTTTAGCATTTCATGATGCAGGAACTTACGATGCTAAAACGAAGACAGGTGGTCCAA
ATGGTTCGATTAGACGTGAGTTGAGCAACCCTCCTAACAATGGTATCAAAGTTGCTGTT
GATTTTTGCGAGCAAGTGAAAGCTAAATACCCCAAGGTTACTTATGCTGACTTTTACCA
GCTTGCTGGTGTGCTTGCGGTTGAAGTAACCGGAGGTCCTAAAATCCCATTTATTCCCG
GTCGTCCTGATGCTAGGGATCAACAAGACAGTGGTGCTCTTCCAAATCCCAATGGAGA
TGCCAAACATCTAAGAGACATATTTTACCGTATGGGTTTGAACGATAGAGATATCGTGG
TCTTATCGGGGGCTCACACACTGGGTCGAGCAAACAATGATAGATCTGGCTTTGATGGT
CCTTTCACAGAGGACCCTTTCAAATTCGACAATTCCTATTATGTAAACTTGAGGAAGGA
AGACAAACCTGGACTGGTGAAATTTCCCACTGACAAAGTTCTCGTCGAGGACCCTGCT
TTTAGCAAATATGTTCAATTGTATGCCAAGGATGAACAAGCTTTCTTCGCACACTATGCT
GAATCACACAAGAAGATGTCGGAGCTCGGGTTTCACCCACCCAGCCCTGGTGCTAAAG
CTTGA
>CpAPX10b

ATGGCACATCAACAACAATCGCGAGCAGGGCACATTGTGGATGCCCAATACTTGAAAC
AAATTGAAGGAGCTCGTAGAGACCTTCATCATCTTCTTCAAAATAACAAGAAATTTGCA
CCCATTGTGCTTCGATTAGCATTTCATGATGCAGGTACTTATGATGCCAAAACAAAAAC
TGGAGGTGTAAATGGCTCAATTAGGAATGAGTTGAACAACCCTCCCAACGGCGGTATT
AAGAATGCTATTGATTTTTGTGAACAAGTGAAGATCAAACACCCCAAAGTCACATATGC
TGACCTTTACCAGCTTGCTGGTGTTGTTGCTGTTGAAGTTACCGGAGGTCCTGTTATTC
CTTTTATCCCTGGTCGACCGGATGCCGTGGATCAACAAGACCGCGGAGACCTTCCAAA
TCCTAATGGAGGTTCCCAGCATCTGAAGGATATATTTTATCGAATGGGCTTGAACAATAG
GGATATTGTTGTTCTATCTGGAGCTCACACACAGGGTCGAGCACACAAGGATATATCGG
GTTTTGATGGTCCTTTCACCAAAGATCCTTTGAAATTTGACAATTCCTATTATGTAAACT
TGAGGAAGGGTGAAACTCCTGGACTGGTGCAATTCCCTACAGACAAAGTTCTTGCCGA
GGACCCTGATTTCCGTAAATATGTTCAACTGTATGCTAAGGATGAAAAGGTCTTCTTTG
CACACTACGCGGACTCACACAAGAAGATGTCAGAGCTCGGGTTTCACCCAGGGCACC
CATGCTGCCACTCTAAGAAAGCCTGA
>CpAPX10c
ATGTCCGATCCTTTATCAATGGCGTCATCCTTCTCTTCCACCGCCTCCGCCGCCGCCGCC
GCCGCCGCTGCAACCGCCGCTTCTCGTCTTCTTCCTCCGACTTCTATCTCTCGTCTTTCT
CTCTCCCCTTCTTCTTCTTCTTCGTCTTCCTTCAAATGTCTCCGATCATCTCCGCTTGTTT
CTCACCTTTTTCTGAGATAGAGAGAAGGTTCGGTTTATGTGACGAAGTCGAGGTTTAGT
ACGAAGTGTTATGCTTATGCTTCGGATCCTGCGCAGTTGAAGAATGCTAGGGAGGATAT
TAAAGAGCTTCTTCAGACTAAGTTTTGTCATCCGATTATGGTGCGCTTAGGTTGGCATG
ATGCTGGTACTTATAATAAGGATATTAAAGAATGGCCACAGAGAGGTGGAGCCAATGGC
AGTCTGAGGTTTGAGGCTGAACTCAAACACGGAGCTAATGCAGGTCTTATAAATGCAT
TGAAACTTCTCCAGCCCATAAAAGACAAGTACTCTGGAGTGACATATGCAGATCTATTC
CAGTTGGCTAGTGCTACTGCAATAGAGGAGGCTGGTGGTCCTAAGATACCAATGAAGT
ATGGAAGAGTGGATGTGACAGGTCCAGAGCAGTGCCCAGAAGAAGGAAGGCTTCCTG
ATGCTGGACCTCCTTCACCTGCTCAACATCTACGTGATGTTTTCTACAGAATGGGCTTG
AATGATAAGGATATAGTAGCATTATCTGGAGCACATACATTGGGAAGGTCTAGACCTGA
GCGCAGTGGTTGGGGCAAACCAGAGACTAAATACACGAAAGATGGACCGGGAGCTCC
AGGAGGGCAGTCCTGGACTGTGCAGTGGTTGAAGTTTGATAATTCCTATTTTAAGGAC
ATCAAAGAGAAGAGAGATGCAGATTTGCTTGTTTTGCCAACTGATGCTGCTCTTTTTGA
AGATCCATCTTTCAAGGTATATGCTGAGAAATACGCAGCTGATCAGGATGCATTTTTCA
AGGATTATGCTGAATCCCATGCCAAGCTGAGCAACCAAGGAGCCAAATTTGACCCTCC
TGAGGGTTTCTCTCTTGATGGAAGTCCAGCTGGAGCAGCGCCAGAGAAGTTTGAAGC
AGCCAAGTACTCATCTAACAAGGTTTGAGAGCTATCGGACTCCATGAAGCAAAAGATC
CGCGCTGAATACGAAGGTTTTGGTGGTAGCCCTGATAAGCCTCTCCCAACAAACTACTT
CCTAAACATTATGGTTGTGATTGGAGTTTTGGCAATTCTATCATATCTTGCAGGAAATTG
A
>CpAPX10d
ATGGCAGCCACAACAAAGAGCGTAGTTGATGTAGATTATCTGAAACAAATTGAGGGTG
CTCGTAAAGATCTTCTTGATCTTCTTCAGAAGAACAAGAATTTTTCACCCATTGTGCTT
CGTTTAGCATTTCATGATGCAGGAACTTACGATGCTAAAACGAAGACAGGTGGTCCAA
ATGGTTCGATTAGACGTGAGTTGAGCAACCCTCCTAACAATGGTATCAAAGTTGCTGTT
GATTTTTGCGAGCAAGTGAAAGCTAAATACCCCAAGGTTACTTATGCTGACTTTTACCA

GCTTGCTGGTGTGCTTGCGGTTGAAGTAACCGGAGGTCCTAAAATCCCATTTATTCCCG
GTCGTCCTGATGCTAGGGATCAACAAGACAGTGGTGCTCTTCCAAATCCCAATGGAGA
TGCCAAACATCTAAGAGACATATTTACCGTATGGGTTTGAACGATAGAGATATCGTGG
TCTTATCGGGGGCTCACACACTGGGTCGAGCAAACAATGATAGATCTGGCTTTGATGGT
CCTTTCACAGAGGACCCTTTCAAATTCGACAATTCCTATTATGTAAACTTGAGGAAGGA
AGACAAACCTGGACTGGTGAAATTTCCCACTGACAAAGTTCTCGTCGAGGACCCTGCT
TTTAGCAAATATGTTCAATTGTATGCCAAGGATGAACAAGCTTTCTTCGCACACTATGCT
GAATCACACAAGAAGATGTCGGAGCTCGGGTTTCACCCACCCAGCCCTGGTGCTAAAG
CTTGA
>CpAPX1
ATGGGAAAGAGCTACCCAACCGTCAGTGAGGATTACCAGAAATCTATTGAAAAGGCCA
AGAGGAAGCTCAGGGCTTTGATTGCAGAGAAGCAGTGTGCTCCTCTTATGCTCCGTCT
TGCATGGCACTCTGCTGGTACCTTTGATGTGAACTCAAAGACTGGAGGACCTTTCGGT
ACAATTAGGCACCAGGCAGAGCTGGCTCATGGGGCTAACAATGGGCTTGACATTGCCG
TTAGGTTGTTGGAACCCCTCAAGGAACAATTCCCCGAAATTTCTTACGCTGACTTTTAT
CAGTTGGCTGGTGTTGTGGCTGTTGAAGTCACTGGAGGACCTGAAATTCCCTTCCACC
CAGGCAGAGAGGACAAGCCAGAGCCACCCCAGGAAGGCCGACTCCCTGATGCCACC
AAGGGTTGTGACCATTTAAGAGATGTCTTCATCAAGCAAATGGGTCTAACTGACCAGG
ACATTGTTGCTCTCTCTGGAGGCCACACCTTGGGGAGGTGCCACAAGGAACGCTCTGG
GTTTGAGGGAGCTTGGACTGCCAACCCTTTGGTCTTCGACAATTCTTATTTCAAGGAGC
TCCTGAGTGGTGAGAAGGAAGGTCTCTTGCAGCTTCCATCTGACAAGGCTCTTCTCTC
TGACCCTGTCTTCCGCCCACTGGTTGAGAAATATGCAGCTGATGAGGATGCATTCTTTG
CCGACTATGCTGAGGCGCACCTGAAACTTTCTGAACTTGGATTTGCCGATGCTTAA
>CpAPX6
ATGACCACCTCCGGCGGCGAATCGTCGATTTTCCGCGGCTGCTTAGCCACCCAAAAAT
CATCAAATCCCCTCAATAAATTCAAATTTCCCGCCAAAACTTCGCCGTTAACCGTCCAA
TTCGGCGCCAAAGTAGCTCCCTTTCTCTCTCCTCCCTCTGCTCTTCCACCCACTTCAGA
TGGTTATGGCAATCTCGGCTCGTGTGGGAGGAGAGGACTCATTTTCCTTTCTGTCACTC
TACCTTCTTTATTCCCTTTGCAAGAATATGTCAAAGATTTCAATGCTGAAGCAGAAGAG
CTGGGAGCTCCTGATTACCAACTTATGAAGGATGAGATAAGGAAGGTTTTGTCCAAAG
GAAAGGCTGCTGGTGTACTTCGTCTCGTGTTTCATGATGCAGGAACCTTTGAGCTCAGT
GAGAATTCAGGAGGTATGAATGGCTCTATAACATACGAACTTGAAAGGCCTGAGAATA
AAGGGTTAAAAAAGTCTCTAAAGATCATTGAGAAAGCCAAAAATGTAGTGGATGAGA
GGCAATCAGTGTCCTGGGCAGACATGATTGCTGTAGCAGGAGCTGAGGCAGTCTCAAT
TTGTGGAGGTCCGGATATTCCAGTTCGTTTGGGAAGACTTGATTCGCTGACTCCAGATC
CTGAAGAAAAACTACCCGAGGAATCACTGGATGCACCAAGCTTAAAACAGTGCTTTCA
AAGAAAGGGCTTTTCCACACAAGAACTTGTTGCCCTTTCTGGGGCGCATACTCTTGGA
AGTAAAGGTTTTGGAAGCCCCATCGTATTTGACAATTCATACTACAAAATTCTCCTTGA
GAAACCATGGCTGTTGTCAGCTGGAATGTCAAGTATGGTTGGACTTCCTTCAGATCGTG
CATTAGTAGAGGATGATGAATGCTTAAGGTGGATTACAAATTACGCGAATGACCAAGAT
TTATTTTTTGAGGATTTTAAGAATGTCTATATCAAGTTAGTCAATTCGGGTGTAAGGTGG
AAATCTTCTTGA
>CpAPX1a
ATGGGAAAGAGCTACCCAACCGTCAGTGAGGATTACCAGAAATCTATTGAAAAGGCCA

AGAGGAAGCTCAGGGCTTTGATTGCAGAGAAGCAGTGTGCTCCTCTTATGCTCCGTCT
TGCATGGCACTCTGCTGGTACCTTTGATGTGAACTCAAAGACTGGAGGACCTTTCGGT
ACAATTAGGCACCAGGCAGAGCTGGCTCATGGGGCTAACAATGGGCTTGACATTGCCG
TTAGGTTGTTGGAACCCCTCAAGGAACAATTCCCCGAAATTTCTTACGCTGACTTTTAT
CAGTTGGCTGGTGTTGTGGCTGTTGAAGTCACTGGAGGACCTGAAATTCCCTTCCACC
CAGGCAGAGAGGACAAGCCAGAGCCACCCCAGGAAGGCCGACTCCCTGATGCCACC
AAGGGTTGTGACCATTTAAGAGATGTCTTCATCAAGCAAATGGGTCTAACTGACCAGG
ACATTGTTGCTCTCTCTGGAGGCCACACCTTGGGGAGGTGCCACAAGGAACGCTCTGG
GTTTGAGGGAGCTTGGACTGCCAACCCTTTGGTCTTCGACAATTCTTATTTCAAGGAGC
TCCTGAGTGGTGAGAAGGAAGGTCTCTTGCAGCTTCCATCTGACAAGGCTCTTCTCTC
TGACCCTGTCTTCCGCCCACTGGTTGAGAAATATGCAGCTGTATGTTCTTCCTGA
>CpAPX3
ATGGCGATGCCGGTCGTCGACACAGAATACCTCAAGGAAGTCGACAAAGCTCGTCGC
GATCTTCGCGCTCTCATCTCTGGCCGCAACTGCGCTCCTATCATGCTCCGCCTAGCGTG
GCATGATGCTGGGACCTATTGCGCGAAGACGAAGACCGGTGGTCCGAATGGTTCGATT
AGGAATGAGGAAGAGTGCGCTCATGCTGCCAATAGTGGTTTGAAGAAAGCTGTTGATT
GGTGTGAGGAGGTGAAGGCTAAACACCCAAAGATTACATATGCAGACCTCTATCAGCT
TGCTGGTGTTGTTGCAGTTGAGGTCACTGGAGGACCTACAATTGACTTTGTTCCTGGTA
GAAAGGACTCAAGTGTGTGTCCCAAGGAAGGGCGACTTCCAGATGCCAAACAAGGTG
CACCACATCTGAGGGACATATTTTACAGGATGGGTCTTTCTGACAAAGACATTGTGGCC
CTATCTGGGGCTCATACCCTGGGAAGGGCACATCCAGAGAGATCAGGCTTTGATGGCC
CGTGGACCCAGGAGCCACTCAAGTTTGATAACTCCTATTTTGTGGAGCTTTTGAAAGG
AGAATCAGAGGGACTGTTGAAACTTCCTACTGATACAACTTTGGTGGAGGATCCTGCA
TTTCGCCCTTTTGTGGAATTGTATGCTAAGGATGAGGATGCCTTCTTCCGTGACTATGCA
GCCTCGCACAAGAAACTTTCAGAATTAGGATTCACTCCAAGCGGGGCCAAGTCATGCT
GCCCCGACACCACTGTACTGGCTCAGAGTGCAGTTGGAATTGCAGTTGCTGCGGCAGT
GGTCATCTGTAGCTACATCTATGAAATTCGCAAACGCGCAAAGTAA
>CpAPX9
ATGCTAGGACGAGAAAAAAAACAGTTGGTGGATGGCGAATATTTGAAGGAAATTGAG
AGAGCTCGAAGAGACCTCCGCGCTCTCATCTCTGCCAACCATTGTGCTCCTAGTGTGCT
TCGATTAGCATGGCATGATGCAGGTACATATGATGCAAAGACGAAAACGGGAGGCCCG
AATGGCTCAATCAGAACCAAGGACGAAATGGAGCACAGAGCCAACCGTGGCCTCAAA
ACAGCTGTTGCTCTTTGTGAAGAAATAAAGGTTAAACACCCAAAGATCACTTATGCGG
ATCTTTATCAGCTTGCTGGAGTTGTTGCAGTTGAAGTCACAGGCGGTCCAACCATCGAC
TTTGTTCCTGGACGAGTGGACTCCACGGAATCTCCAGAGGAAGGACGCCTTCCTAATG
CAGATTTGGGTGCTGAACATTTGAGAGAAGTATTTTACAGAATGGGATTTACTGACAAG
GATATTGTCGCGTTATCAGGAGGCCACACACTGGGTGAGGCGCATAAGGAAACATCAG
GTTACGAAGGTCCTTGGACTAAAGACCCAATGAAATTCGACAATTCCTATTATGTAGAG
TTGTTGAATGGTGAACGACGAGGGTTATTGAAACTTCCTACTGATAAGGCTCTTGTAGA
GGATCCTAGATTTCGACGCTATGTTGAGCTCTATGCTAAGGATGAAGACGCGTTCTTTA
GAGATTATGCAGAATCACACAAGAAGCTCTCAGAGTTAGGGTTCAATCCACCCATGCC
TTGGAGAAAGATTGTTTCCAAGGCTGCACTTGGTGTAATTGTCTCTGTTACTGTAATCA
TCTTGTATGAATTAAACAAGAATGAATAG
>CpAPX10e

ATGGCAGCTACCACAAAGAGCGTTGTTGATGTAGATTATCTGAAACAAATTGAGGGTG
CTCGTAAAGATCTTCATGATCTTCTTCAGAAGAACAAGAAGTTCTCTCCCATTCTGCTT
CGCTTAGCATTTCATGATGCAGGTACTTATGACGCTAAAACGAAGACAGGGGGTCCAA
ATGGTTCAATTAGACGTGAGTTGAGCAACCCTCCCAACAATGGTCTCAAAGTTGCTATT
GATTTTTGCGAACAAGTGAAATCTAAATACCCCAAGATTACTTATGCTGACTTTTACCA
GCTTGCTGGTGTCCTCGCGATTGAAGTAACCGGAGGACCTAAAATCCCCTTTACTCCAG
GTCGTCCTGATTCTAGGGATCAACAAGACGGTGGTGCTCTTCCAAATCCTAATGGAGAT
GCCAAACATCTAAGAGATGTATTTAATCGAATGGGTTTTAAGGATAGGGATATCGTGGT
CTTATCGGGGGCTCACACATTGGGCCGAGCAAACAGAGATAGATCTGGCTTTGATGGT
CCTTTCACGGAGGAACCTTTCAAATTCGACAATTCCTATTTTGTAAACTTGAGGAAGGC
TGACAAACCTGGATTGGTGAAATTTCCCACGGACAAAGTTCTCGTCGAGGATCCTGCT
TTTAATAAATATGTTCAGCTATATGCCAAGGATGAAAAAGCTTTCTTTGCGCACTATGCT
GAATCACACAAGAAGATGTCGGAGCTCGGGTTTCACCCACCCAGCCCTGGTGCTAAAG
CTTGA
>CpCAT1
ATGGATCCTTACAAGTTTCGGCCATCAAGTGCGCACAATTCATCACACTTCACCACAAA
TTCTGGTGCTCCTGTTTGGAACAACAACTCTTCTTTGACTGTTGGCAACAGAGGCCCC
ATTCTTTTGGAGGACTATCATCTTGTGGAAAAGCTTGCTAACTTTGACCGAGAGCGTAT
ACCAGAACGTGTTGTTCATGCCAGGGGAGCGAGTGCTAAGGGATTTTTTGAAGTTACT
CATGATATTACACACCTCACATGTGCTGATTTCCTGCGTGCTCCTGGTGTCCAGACACC
TGTGATTGTTCGGTTCTCTACTGTTATTCATGAACGTGGGAGTCCTGAAACCCTCAGGG
ACCCTCGTGGTTTTGCTGTGAAGTTTTACACCAGAGAGGGTAACTTCGATCTAGTGGG
GAACAATTTCCCTGTGTTTTTCATTCGTGATGGTATGAAATTCCCTGATATGGTGCATTC
CCTCAAGCCCAATCCTAAAACCCACATTCAAGAGAACTGGAGGGTCATGGATTTCTTC
TCTCACCATCCCGAGAGTTTACACATGTTCACTTTCCTCTTTGACGATGTGGGTGTTCCT
GCAAACTACAGACACATGGAAGGCTCTGGTGTTAACACATATACTCTGGTCAACAAGG
CTGGCAAAGCATACTATGTGAAGTTCCACTGGAAGCCAACATGTGGAGTTAAGTGTCT
GTTGGAGGATGAATGCGTCAAAGTTGGAGGAGCTAACCACAGCCATGCTACTCAGGAT
CTTACTGACTCAATTGCAGCTGGAAACTATCCTGAATGGAAGCTGTTTATCCAGACAAT
TGACCCTGCTGATGAAGATAAATTTGACTTTGACCCCCTAGATGTTACCAAAACATGGC
CTGAGGACATTTTGCCTCTTCAGCCAGTGGGTCGTATGGTCTTGAACAAGAACATTGA
CAACTTCTTTGCTGAAAATGAGCAGCTGGCTTTTTGCCCTGCTATTGTTGTTCCTGGGG
TCCACTACTCAGATGACAAGCTGCTTCAGACTCGAATTTTCTCATATGCTGATACCCAA
AGGCATCGTCTTGGTCCCAACTACCTGCAACTTCCAGTCAACGCTCCCAAGTGTGCTC
ATCACAACAATCACTATGATGGTTCCATGAACTTTATGCACAGAGATGAGGAGGTGGAC
TACTTTCCATCAAGATATGATCCCGTTCGTCATGCTGAGAGATACCCCATTCCTGCAAAT
ATTTTGAATGGAAGGCGTGAAAGGCAAATCATTGCAAAGGAAAACAACTTTAAGCAG
CCTGGAGAGCGATACAGATCAATGGACCCAGCCAGGCAAGAGAGATTTATCTGCAGAG
TGGTTGATGCTTTAAGCGACCCTCGTGTCACCCATGAGATACGCAGTATCTGGGTTTCA
TACTGGACCCAGGCTGACAAATCTCTGGGTATGAAAGTTGCAAGCCGACTTAATGTGA
GACCAACCATGTAA
>CpCAT2
ATGGATCCATCTAAGGTTCGGCCAGCAAGTGCTTACAACGCCCCGTACTTGACTACCAA
TAATGGTACACCCGTTTGGAACGATACCTCAGCGTTAACTGTTGGCCCTAGAGGTCCAA

TTCTGTTGGAGGATTATCATCTAATTGAAAAGATAGCAACTTGGACACATGAACGGATA
CCTGAACGTGTTGTACATGCTAGGGGAGCAAGTGCTAAGGGTTTTTTGAAGTTACCC
ATGATATCACTCACCTCACTTGTGCTGATTTCCTCCGTGCCCCCGGTGTCCAGACCCCT
GTTATTGTTCGATTTTCCACGGTCATTCATGAACGTGGTAGTCCTGAAACACTCAGAGA
CCCTCGTGGTTTCGCCGTCAAGTTCTATACTAGAGAGGGTAACTTTGATATTGTGGGAA
ACAACTTCCCGGTGTTTTTCACCCGTGATGCAATGCAATTCTTCGACCTGATCCGAGCA
TTTAAGCCCAACCCCAAATCTCACATCCAAGAGAGTTGGAGGTACATGGACTTCTGCT
CATACCTCCCTGAGTCTCTCAACACCTTCACCTTCTTCTTCGATGATGTGGGCATCCCG
ATCAATTACAGACACATGAATGGCTCCGGAGTCCACACCTTCACTCTCATCAACAAAG
CAGGGAAAGTAACATATGTCAAGTTCCATTGGCGCCCAACTTGCGGCGAAAAGAATTT
GCTCGAGGAGGAGGCCATCAGGGTTGGTGGCTCGAACCACAGCCACGCGACTCAGGA
CTTGTACGAGTCTATTGCTAATGGGAACTTCCCTGAGTGGAAGCTCTACATCCAAACCA
TGGACCCTGCTGATGAGGATAAGTTTGATTTCGATCCACTCGATACTACTAAAACTTGG
CCTGAGGATATCCTGCCCTTACAGCCTGTAGGACGTCTTGTGTTGAACAAAAATGTTGA
CAACTTCTTTACAGAAAATGAGCAGCTGGCTTTTGATCCTGCTCATATTGTTCCTGGGA
TTTACTTCTCTAATGATAAGATGCTTCAGGCTCGAACTTTTGCCTATGCCGATACCCATA
GATATCGTTTGGGTGTCAACTACTTGCAGCTTCCTGTTAATGCTCCTAAGTGCCCTCATA
CCAACATGCACCGCGATGGTGCTATGACTATGGTGCACAGGGATGAAGAGGTTGATTAT
TTCCCATCAAGATTTGATCCTCTAAGAGAAGCCGAGAGACACCCTATTCCAACGGCAAT
CATACATGGAAGGCGTGAAAAGCAAATCATACCAAAGGAGAACAACTTCAAGGAGCC
AGGAGTGAGATATAGATCATGGGATCCAGCCAGGCAAGAGAGATTCATTGCGAGATTT
GTTCAAGCTCTGTCTGATCCACGCCTCACATATGAGATACGCAGCATTTGGGTTTCATAC
CTGTCTCAGGCGGATAAATCATTGGGCATGAAAGTAGCAAGCAGGCTCAATATAAGAC
CAACCATGTAA

CDS sequence of SOD, APX and CAT gene family members in *Silene uniflora*

>SuMSD1
ATGGCGCTTCGATCAATCTTAACCAGGAAAACCTTAGCTTCATCTGCAACTTCACCTCT
CCAATGGCGTTCCTTGACTACCTTCACTCTCCCTGACCTTCCCTACGATTACGGCGCAC
TTGAGCCCGCCATTGATGGTCAGATCATGCAGATTCACCACCAGAAACATCATCAGACT
TACGTCACTAACTACAACAAGGCTCTTGAGCAGCTTCATGATGCGTCCGAGAAAGGCG
ATGCTTCTGCTGTTGTTAAGCTTCAAAGCGCCATTAAATTCAATGGCGGAGGTCATATC
AATCACTCAATTTTCTGGAAGAACCTAGCTCCAATTAAGGAAGGAGGTGGTGAGCCTC
CAAAAGGCTCTCTGGGTTGGGCTATTGACTCAAATTTCGGCTCTGTTGAAGCTCTTATT
CAGAAAATGAACGCCGAAGGAGCTGCCTTGCAAGGTTCTGGTTGGGTGTGGCTAGGA
CTTGACAAGGAGTTGAAAAGGCTTGTGGTTGAAACGACTGGTAATCAGGATCCACTGG
TAACTAAAGGGGCAACTTTGGTTCCTTTACTTGGTATAGATGTCTGGGAGCATGCCTAC
TACTTACAGTACAAGAATGTGAGACCTGATTATCTGAAGAACATATGGAAGGTTATGAA
CTGGAAATATGCCAGTGATGTTTACGAGAAAGAGTGTCCTAGCCCCTAA

>SuMSD2
ATGGCGCTTCGATCAATCTTAACCAGGAAAACCCTAGCTTCATCTGCAATTTCACCTCT
CCAATGCCGTTCCTTGACTACCTTCACTCTCCCTGACCTTCCCTACGATTACGGCGCAC
TTGAACCCGCCATTGATGGTCAGATCATGCAGATTCACCACCAGAAACATCATCAGACT
TACGTCACTAACTACAACAAGGCTCTTGAGCAGCTTCAAGATGCGTCCGAGAAAGGCG
ATGCTTCTGCTGTTGTTAAGCTTCAAAGCGCCATCAAATTCAATGGCGGAGGTCATATT

AATCACTCAATTTTCTGGAAGAACCTAGCTCCAATTAAGGAAGGAGGTGGTGAGCCTC
CAAAAGGTTCTCTGGGTTGGGCTATTGATTCAAATTTCGGCTCTGTTGAAGCTCTTATT
CAGAAAATGAATGCCGAAGGAGCTGCCTTGCAAGGTTCTGGTTGGGTGTGGCTAGGA
CTTGACAAGGAGTTGAAAAGGCTTGTGGTTGAAACGACTGGTAATCAGGATCCACTGG
TAACTAAAGGAGCAACTTTGGTTCCTTTACTTGGTATAGATGTCTGGGAGCATGCCTAC
TACTTACAGTACAAGAATGTGAGACCTGATTATCTGAAGAACATATGGAAGGTTATGAA
CTGGAAATATGCCAGTGATGTTTACGAGAAAGAGTGTCCTAGCCCCTAA
>SuFSD2
ATGGGAAGTTTTACTACTACTACTACTACTCTCCATTGTAATGGTTTTTCTCCAACCAGT
AAAATCCCAAGCTTTATTCCTCAGAAGCGTATAGGATGTCTTGATCGGAGAAGAGGAC
CTAAAGTAGTTGCATATTATGGGCTAAAAAAGCCTCCTTATAACCTGGATGAATTGGAA
CCATATATGAGCAGAAAGGCACTCGAGTTACATTGGGGACAGCATCATAAAGGCTACCT
GGATGCTTTAAACAGGCACCTAAGTAAGAGTGATATACTATATGGCTATACCATGGATGA
GCTTGTTAAAGTGACATACAACTACGGAAACCCCTCACCGGAATTCAACGATGCTGCA
CAGGTTTGGAACCACGAATTTTTCTGGGACTCTATGCAACCCGGAGGTGGGGACTCAC
CAAAGCTTGGACTGCTTCTGCAGATTGAGAAAGATTTTGGTTCTTTCACTAATTTTAAA
GAGAAATTTACCGAAGCAGCCCTTACATTATTTGGTTCTGGCTGGGTTTGGCTTGTTTT
GAAGAAGCACGAAAAGCGTCTAGTGATAGTTAAAACATCAAATGCAATCACTCCCATT
GTATGGGACGATATACCCTTATTATGTTTAGATATGTGGGAGCATGCTTATTATTTGGATT
ACAAGAATGATAGAGCCAAGTATGTAAATGTATTCATGAACCACCTTGTATCATGGCAT
GCAGCCACTGCACGAATAGCTCGAGCTCAGTCGTTTGTTAACCTAGGCGAACCTAAAG
TCCCGGTTGCTTAA
>SuCSD1
ATGGGAAAGGCTGTCGTCGTTTTGAACAGCAGTGAGGGTGTCAAAGGAACTGTCCAC
TTCACTCAAGAAGGAGATGGTCCAACAACCGTGACTGGATCTATCTCGGGTCTCAAGG
CTGGTCTTCATGGATTCCATGTTCATGCCCTTGGTGACACCACAAACGGATGCATGTCA
ACAGGACCACACTTTAACCCTGCTGGAAAAGAGCATGGTGCTCCAGAAGATGAAGTT
CGCCATTCTGGTGACCTTGGAAACATCACAGCTGGGGATGATGGTATGGCAACCTTCA
CAATCACTGACTGTCAGATTCCTCTTTCTGGACCACACTCTATTGTTGGAAGGGCTGTT
GTTGTCCATGCTGATCCCGATGACCTTGGAAGAGGGGGCCATGAGCTCAGCAAGACCA
CTGGTAATGCTGGAGGAAGAATTGCTTGCGGTATTATCGGTCTTCAAGGCTAA
>SuFSD1
ATGTCTGCTGTTGCTGCGACTTGTACCATTTCCTTGTCTTCAACCTTTCTTTCTCATGGC
TCTATTTGGGGACCTAAATCTATCCCAGTTACACCTTTCAGATTTAAGGAGGTGCGTGTA
AGAAGAGCTGGAGCTGCTGTAGTAGCCAAGTTTGACTTGAAGCCTCCGCCATATCCAT
TTGATGCCTTGGAGCCTCACATGAGTAAACAGACTTTTGAGTTTCATTGGGGAAAGCAT
CATAGAGCATATGTGGATAACCTGAACAAACAAATTGTTGGAACAGAGTTAGATGGGA
TGTCACTTGAAGATGTAGTGAGCGTAACATATAACAAAGGTGACATGCTTCCATCTTTC
AATAATGCAGCACAGGCCTGGAACCACCAGTTCTTTTGGGAGTCAATGAAACCTGGTG
GCGGGGGGAAGCCATCTGGAGAACTTTTGGCTCAAATTGAGAAAGATTTTGGTTCTTT
TGAAGCATTTGTTACAGAATTCAAGTCTGCTGCTGCCACGCAATTTGGTTCTGGTTGGG
CTTGGCTTGCTTACAAAACGGATAAGTTGGATGTGGGTAATGCGGTAAACCCTAAACC
GTCTGAAGATGACAAAAAGCTTGTGATTGTTAAAACGCCCAATGCTGTAAACCCTCTT
GTCTGGGGTTATAATCCTCTTCTCACCATTGATGTTTGGGAGCATGCATATTACTTGGAC

TACCAGAATCGAAGGCCTGATTACATTTCAATTTTCATGGAGAATCTTGTTTCCTGGGAT
GCAGTTAGTGCGAGATATGAGACCGCCAAGGCACTTGCTGCTTAG

>SuCSD4

ATGACTTTGCCCGAATTAGTGACGGAATTCATGGTGTATATGAAGTGCGAAGGATGTGT
AGATTCTGTCACAAATAAATTAGAGACGGTTCAAGGAGTGAAGAGTATTGAAGCGGAC
TTGAGCAATCAAGTAGTTAGAATTCGAGGCTCTTCTCCTGTCAAAACCTTGGAAGACG
CTTTACTGCAAACAGGTCGTACTGCTCGGCTTATTGGTCAAGGACTGCCTGATGATTTT
CTCGTATCTGCGGCTGTGGCTGAATTCAAAGGCCCTGATATATTTGGAGTTGTTCGATTA
GCCCAGGCAAATATGGAATTAGCTAGAATTGAGGCCAATTTTAGTGGGTTGCCACCTGG
AAAGCATGGTTTCTCAATAAATGAATATGGTGATCTGACAAGAGGTCCAGCTAGTACCG
GGAAAATGTACAGTCCGACAATCCAAGGCAGTGAAACTGAGGTATTCTCCCCTTGTTC
AAATAAGATTGATCACCATTTCATTTAG

>SuAPX6

ATGACGACCACCACCTGCAACAAACCACCATTTCTAAGCTGCTGCTTCACCTCCGTATC
ATCTTCTTTCATCACCAATTTCAATTTTCCCGCCAAATTTCAACCGCCAACTATCAATTT
TGGCGCCAAATCAACTCCATTGCTCAAACCCTCTTGCGCGATTAAACCATCGTCTGGTG
ACTTTGGGAACCTTGAATCATGTGGAAGGAGAAGGCTTCTTTTGCTTTCTGCAACAGT
GCCTTTTCTTATTCATATGCAGGAATACACCAAAGATTTCTATGCTGAAGCGGAAGAGC
TGGAAAGTTACGGGGACCAAATTATAAGAGATAAAGTAAGGAAGGTTTTGACTAAAGG
GAAGGCAGCTGGTATTCTTCGTCTGGTTTTTCATGATGCAGGAACCTTTGAAAGAAAC
GACAATTCAGGTGGTATGAACGGCTCTGTAACATATGAACTTGAAAGACCAGAGAATA
AAGGGCTCAAAAAATCTTTGAAGGCATGCTTTCACACTTTTTCCTCTCGACCTCTTTCT
CCTTATAATATTCTTGAGAAAGCCAAGAATGAGTTGGATGTGCAAGAATTAGTGTCATG
GGCTGACCTGATTGCTGTAGCTGGAGCTGAAGCAGTCTCAATATGTGGAGGTCCAGTG
ATTCCTATTCGGTTAGGAAGACTTGATTCACTGACACCTGATCCTGAAGGGAAGCTGCC
TGAGGAATCACTAGATGCACCTGGACTGAAGCAGTGCTTTAAAAGAAAAGGCTTCTCG
ACACAAGAACTTGTAGCATTGTCTGGAGCCCATACAATTGGTAGTAAAGGTTTCGGAA
GTCCCATTGAATTTGATAATTCATACTACAAAATCCTACTTGAGAGGCCCTGGGTATCAG
CAGGTGGAATGTCAGGTATGGTTGGACTTCCTTCAGATCGTGCACTTGTTGAGGATGA
AGAATGCTTAAGATGGATTACAAAATACGCGAAAGATCAAAATCTGTTTTTCGATGATT
TTAAGAATGCTTACATCAAGTTGGTCGATTCAGGCGCAAGATGGAAATCTTCATGA

>SuAPX1

ATGGCAAAGAACTACCCAACTGTGAGCGAGGACTACCAAAAGGCCATTGAGAAGGCA
AAGAAGAAGCTCAGAGGTCTTATCGCTGAGAAGAGCTGTGCTCCACTTATGCTTCGTA
TTGCATGGCACTCTGCAGGTACTTTTGATTGCCAGTCAAAGACTGGAGGACCCTTCGG
TACAATGAGGTTCAAGGAAGAGCTGGCTCATGGAGCCAACAACGGGGTTGACATTGC
AGTCAGGTTGTTGGAGCCTATCAAGGAACAATTTCCCACCATCTCTTACGCTGACTTCT
ATCAGTTGGCCGGTGTTGTGGCTGTTGAGATTACTGGAGGACCTGAAGTTCCCTTCCA
CCCTGGCCGAGAGGACAAGCCAGAGCCTCCACAGGAGGGTCGTCTCCCTGATGCTAC
CAAGGGTTGTGACCATCTGAGAGACGTGTTCGTCAAGCAAATGGGTCTTTCTGACCAA
GACATTGTTGCTCTCTCTGGAGGCCACACCTTGGGAAGGTGCCACAAGGACCGTTCAG
GTTTTGAAGGAGCTTGGACCGCTAACCCTCTGATCTTTGACAACACCTACTTCACGGA
GCTTTTGAGCGGTGAGAAGGAAGGTCTCCTGCAACTACCATCTGACAAGGCTCTTCTG
GCCGACCCCGTCTTCCGCCCTCTCGTTGAGAAATACGCAGCGGATGAAGATGCGTTCT

TCGCCGACTATGCTGAGGCTCACATGAAGCTGTCCGAGCTTGGGTATGCTCTTGCTATG
TTCACACTTAA
>SuAPX1a
ATGGCAAAGAACTATCCAACTGTGAGCGAGGACTACCAAAAGACCATTGAGAAGGCA
AAGAAGAAGCTCAGAGGTCTTATCGCTGAGAAGGGCTGTGCTCCACTTATGCTTCGTA
TTGCATGGCACTCTGTAGGTACTTTTGATTGCCAGTCAAAGACTGGAGGACCCTTCGG
AACAATGAGGTTTAAGGAAGAGTTGGCTCACGGAGCCAACAACGGGATTGACATTGC
AGTCAGATTGTTGGAGCCTATCGAGGAACAATTCCCCACCATCTCTTACGCTGACTTCT
ATCAGAAATTTACCGTCTTTTACTTTTGCAGGACAAGCCAGAGCCTCCACAGGAGGG
TCGTCTCCCTGATGCTACAAAGGGTTGTGACCATCTGAGAGACGTGTTCGTCAAGCAA
ATGGGTCTTTTTGACCAAGACATTGTTGCTCTCTATGGAGGCCACACCTTGGAAAGGTG
CCACAAGGACCGTTCAGGTTTTGAAGGAGCTTGGACCGCTAACCCTCTGATCTTTGAC
AACACCTACTTCACGGAGCTTTTGAGCGGTGAGAAGGAAGGTCTCCTACAACTACCAT
CTGACAAGGCTCTTCTAGTCTACCCTGTCTTACGTCCTCTCGTTGAGAAATATGCAGCG
GTATGTTTTTACTTTAAGCTCTTATTTTTCCAATACCTTTTCCCCAATAGACCTCTTCCAC
CTATCCCTGATTTGGAAGTGCCTGAGAATTCGATTGAGTAG
>SuAPX9a
ATGACAAGCCGAAAAGGGCAGATGATCAATGATGCAGAATACTTGAAAGAAATCAATA
GGGCTCGTCGAGACCTACGTGCACTAATAGTTACCAAGCAATGTGCTCCTATCATGCTG
CGCCTAGCATGGCATGATGCAGGTACATATGACGCGAAAACAAATACTGGCGGTCCAA
ATGGTTCAATTAGGTACGAGCTGAGCCATGGTGCCAATAGAGGCCTCAAAGTTGCCATT
GATTTATGCGAAGAAATAAAGGCGAAACACCCCAGGATTACTTATGCCGACCTCTACCA
GCTTGCTGGAGTCGTTGCTGTTGAAGTCTCAGGTGGTCCAACCATCGACTTTGTTTCA
GGTCGAATGGATTGCCGGAGTTCTACTGATGAAGGCCGTCTTCCTGATGCTAATGGAGG
TAGTGAACATTTGAGAGAGATTTTTTATCGCATGGGTCTTTCTCACAAGGACATTGTTG
CACTTTCTGGAGCACATACACTGGGTAGAGCACATGTAGAGAGATCGGGTTTTGAAGG
CGCTTGGACACCGGAGCCTCTCAAGTTTGACAACTCCTACTTTGTTGAATTGCTGAAA
GGAGAAAAATCGGGGATGCTGAAATTACCTACAGACAAAGTTCTTGTACAAGATCCGG
TGTTCCTTCACTATGTAGAACTCTATGCTAAGGATGAAGAAGCCTTCTTCAGAGATTATG
CAGATTCTCACAAGAAGCTATCAGAGTTGGGATTTTCACCACCTATCTCTCGCCCCAAG
ATGGTTACCAAGGTTGTGGTTGTCATCGCATTTGCTGTTACCGTTGCTGTCTTCAGCTAT
TTCTGTGAAGTTTAA
>SuAPX9b
ATGACAAGTCGAAAAGGGCAGATGATCAATGATGCAGAATACTTGAAAGAAATCAATA
GGGCTCGTCGACACCTACGTGCACTAATAGTTACCAAGCAATGTGCTCCTATTATGCTG
CGCCTAGCATGGCATGATGCAGGTACATATGACGCGAAAACAAAGACTGGCGGTCCAA
ATGGTTCAATTAGGTACGAGTTGAGTCATGGTGCCAATAGAGGCCTCAAAGTTGCCATT
GATTTCTGTGAAGAAATAAAGGCGAAACACCCCAGGATTACTTATGCCGACCTCTACC
AGCTTGCTGGAGTCGTTGCTGTTGAAGTCTCAGGTGGTCCAACCATCGACTTTGTTTC
AGGTCGAATGGATTGCCGGAGTTCTACTGATGAAGGCCGTCTTCCTGATGCTAATGGAG
GTAGTGAACATTTGAGAGAGATTTTTTATCGCATGGGTCTTTCTCACAAGGACATTGTT
GCACTTTCTGGAGCACATACACTGGGTAGAGCACATGTAGAGAGATCGGGTTTTGAAG
GCGCTTGGACAACGGAGCCTCTCAAGTTTGACAACTCCTACTTTGTTGAATTGCTAAA
AGGAGAAAGATCAGGGATGCTGAAATTACCTACAGACAAAGTTCTTGTACAAGATCCG

GTGTTCCTTCACTATGTCGAACTCTATGCTAAGGATGAAGAAGCCTTCTTCAGAGATTA
TGCAGATTCTCACAAGAAGCTGTCAGAGTTGGGGTTTGCACCACCTAATAACTCTCGC
CTGAAGATGGTTACGAAGGTTGTGGTTGTCATCGCATTTGCTGTTACCGTTGCTGTCTT
CAGCTACTCGTGTGAAGTTTAA

>SuAPX9

ATGACAACTCGAAAAGGGCAGGTGAATGATGCAGAATACTTGAAAGAAATCGATAAG
GCTCGTCGAGACCTACGTGCACTAATATTTACCAAGCAATGTGCTCCTATCATGCTGCG
CCTAGCATGGCATGATGCAGGTACATATGATGTGAAAACAAAGACTGGCGGTCCAAAT
GGTTCAATTAGGTACGAGTTGAACCACGGTGCCAATAAAGGCCTCAAAGTTGCCATTG
ATTTCTGTGAAGAAATAAAGGCTAAACACCCAAGGATCACTTATGTAGACCTTTACCAG
CTTGCTGGAGTCGTTGCTGTGGAAGTCTCAGGTGGTCCAATAATCGACTTTATTCCAGG
TCGAATGGATTGCCCCGGTCCTACTGATGAAGGCCGTCTTCCTGATGCTAATGGAGGTG
GCGAACATTTGAGAGAGATTTTTTATCGCATGGGTTTTTCTGACAAGGACATCGTTGCA
CTTTCTGGAGCACATACACTGGGTACAGCACATGCAGATATATCAGGTTTCAAAGGAGC
TTGGACACCGGAGCCTCTCAAATTTGACAACTCGTACTTTGTTGAAATGCTGAAAGGA
GAAAGAGCAGGGATGCTGAAACTACCTACAGACAAAGTTCTTGTACAAGGTCCGGTG
TTCCGTCCCTATGTTGAACTCTATGCTAAGGACGAGGAAGCCTTCTTCAGAGATTATGC
AGACTCTCACAAGAAGCTATCAGAGTTGGGATTTGCACCACCTAACTCGCGCCGGAAA
ATGGTTACGAAGGTTGTGGTTGGTATTGCTATTGCTGTTACCGTTGCTGTCTTCAGCTAC
TTCTATGACATTTATAAGCAAGCTAAGTAA

>SuAPX3

ATGGCGATGCCGGTCGTTGACACGGAATACCTCAAACAAATCGACAAAGCTCGTCGCG
ATCTCCGCGCTCTCATCTCTAGTCGCAATTGCGCTCCTCTCATGCTTCGCCTCGCGTGGC
ATGATGCTGGAACTTATGATGCGAAGACGAAGACAGGTGGACCGAACGGTTCGATTAG
GAATGAGGAAGAGTTTGCTCATGGTGCTAATAACGGTCTCAAGAAAGCTATTGATTGGT
GTGAGGAGGTGAAAGCCAAGTATCCAAAGATTACATATGCCGACTTTTATCAGCTTGCT
GGTGTTGTTGCAGTTGAAGTCACAGGAGGCCCGACTGTTGATTTTGTTCCTGGTAGAA
AGGACTCCAAGGTATCTCCCAGAGAAGGGCGACTTCCAGATGCCAAACAAGGTGCAC
CACACCTGAAGGACATATTTTATCGGATGGGTCTTAGTGACAAAGATATCGTAGCCTTG
TCAGGCGGACACACCTTGGGAAGGGCACATCCAGAGAGATCAGGTTTTGATGGTGCAT
GGACCCAAGAGCCCCTCAAGTTTGATAACTCATATTTTGTAGAGCTGCTGAAAGGAGA
ATCAGAGGGACTGTTGCAGCTTCCGACTGACAAAGCTCTGTTAGATGATCCTGTATTTC
GCCCTTACGTTGAACTCTATGCTAAGGATGAGGAAGCTTTCTTCCGCGACTATGCTGTT
TCGCACAAGAAACTTTCTGAACTGGGCTTTACTCCTGGTGGGTCCAAGTCAGGGCCAA
AGGACAGCATTATACTGGCCCAAGGTGCGGTCGGGGTTGCAGTTGCTGCAGCTGTGGT
TATCCTGAGCTATATCTATGAAGTTCGCAAGAGAGTAAAGTAG

>SuAPX10b

ATGGCACAAATGACAAGATGTGGGCAAATGGTGGATACTGAATATATGAGACAGATTGT
AGAAGCTCGTATTGATCTTCACAATCTATTATCCCGTAATAAGGAATTCGCTCCTACCAT
TCTTCGCTTAGCATTTCATGATGCTGGAACATATGACGCCAAAACAAACACAGGTGGTG
TAAATGGTTCAGTTAGGAACGAAATGAGCACCGCGCCTAATGTTGGCATGCAAGCAGC
TCTTGATTTCTGCGAGCCGGTGAAAGTGAGGCACCCCAAGGTCACTTATGCCGATCTTT
ATCAGCTTGCCGGAGTTGTTGCAGTAGAAGTGACTGGCGGTCCAAAGATAGAATTCGT
CCCTGGTCGGAAGGATGTCTCGCCGCAGCAAGACCAAGGAACTCTTCCCGGTCCCAAT

GCAGATAGCAATCAATTGAGGAACATATTTCACAGGATGGGTCTCAATGACAAGGATAT
TGTGGTACTATCTGGGGCTCACACACTGGGTAGGGCACATCAGGATCGATCAGGCTTTG
ATGGTCCTTTCACCAGAGACCCGCTCACATTCGACAATTCCTATTATGTGCTTTTGACGC
AGGGTGACGCTCCAGGGCTAGTCAAATTTCCCAGTGACAAAACTCTCATGCAAGATCC
TACATTCCGTCGCTATGTTCAAATTTATGCCAAGGATCAGAATCGGTTCTTCAAACATTA
TGCAAAGTCACACAAAAAGATGTCAGAGCTTGGATTTCATTACCCAAATAAACGTAAC
TCTCGCAAACACCAGACCTCACACTCGCATGAGATACCCAACCACAACCAGAACCACT
GCCAACATAACCAAAACCACCATGCTCAGCCACAGCAACACGCTCAGTGCCGTCCAC
GCGCACCCTGTGGATACTGA
>SuAPX10a
ATGGCAAAGGTAGATGCAGACTATTTGAAGCAAATCGAGGCTGCTCGCAATGAGCTTC
GCAACATTATCCGCTCCCAAAATTGCGCTCCTATTATGCTCCGCTTAGCATTTCATGATG
CAGGGACATTTGATGCGAAAACGAAGACAGGAGGTCCAAATGGTACAATTAGGCTGG
AGTTAAGCGCCCCTCCAAACAATGGCATCAAAGTTGCTGTCGATCTTTGTGAACCAGT
GAAGGCTAAACACCCCAAAGTTACCTATGCTGACATTTATCAGGACGCGCCGCAACAA
GACAGCACTGGAGGTCTTCCAAATCCTGGTGGAGATGCTCAACATTTGAGAGATGTAT
TCCACCGAATGGGTCTTTCTGACAGGGATATTGTCGCGCTTTCCGGCTCTCATACACTG
GGTAGAGCACATCAGGATAGATCAGGCTTTGACGGTCCGTTTACCCCACAACCTTTGA
AATTCGACAATTCATACTTCGTAGAGCTGAGGAAAGGTGAAACACCCGGATTGGTGAA
ATTTCCAACTGATAATGTTTTGACAAAAGATCCAGTGTTCCGCAAATATGTCCAGATCTA
TGCTCAGGACGAGAAGACATTTTTCGCTCACTATGCAGAATCACACAAGAAGATGTCT
GAGCTTGGATTCCGCCCACCAGCCCGTGCTTAA
>SuAPX7
ATGGCAACCCTAACTGGCACCGCCGCCGCCGCCGCCGCAACAACCACCACTCGCCGAT
TCCTAATCCACTCATCTTCTTCATTATCATCATCATCTTCATCGTCGGTGAAATGCGTCAG
GCCGTCGCATACGATATCGAATTTGTTCATGAGAAAAAGGAGTGGTGAACGAGGTGGA
ATGCATTTGATTAGGTCAAATTCGAGTCGGAATTTTACGACGAAATGTTCTGCTTCTGAT
CCTCAGCAGTTGAAGAGCGCGAAAGAAGATATTAAATCGCTTCTTAATTCCACATTTTG
TCATCCTATTCTTGTACGATTAGGATGGCATGATTCAGGTACTTATAACAAGGATATCCA
AGATTGGCCTCAACGAGGTGGAGCTAATGGCAGTCTCCGCTTTGAGGTTGAACTTAAA
CATGGAGCTAATGCTGGTCTTGTGAATGCCTTGAAACTTATTCAGCCCATTAAGGACAA
GTATTCCGGAGTCACTTATGCAGATCTATTCCAGTTGGCCAGTGCCACAGCAATTGAGG
AGGCTGGTGGTCCGAAAATACCAATGAAGTATGGAAGAGTGGATGTGACAGGACCCG
ATCAGTGCCCAGAAGAAGGAAGACTTCCTGCATTGTTTCTTCCTCTGAAATTATGGCAG
AAAAAATTTGCATTATCGTCTATTCTTCTGGCATTTATGTTAAACACATCTTCATTGTGTA
CTTTTAGAGCTCGTAGTTGTTCTATTGCGACCTATGTTTTTCCTGCTCTTACTTCCCACTC
CTCAACTGATTCTTGGTATTTGGTTGCTCAGATGCTGGACCTCCTTCACCTGCTCAACAT
CTACGTGATGTCTTCTACAGAATGGGTCTCAGTGACAAGTAATCTGAGTTTCATCTTCTT
GTATGAGTTTCTGAAGCAGAAAGATGGTCCAGGAGCTCCTGGTGGACAGTCGTGGAC
AGCACAATGGTTGAAGTTTGATAATTCGTACTTCAAGGACATCAAAGAAAAGAGAGAT
GAAGATTTGCTCGTCTTGCCAACTGATGCTGCTCTTTTCGAAGACCCATCTTTCAAGGT
GTATGCTGAGAAATATGCAGCAGATCAGGAGGCGTTTTTCAAAGACTATGCTGAGGCA
CACGCAAAGCTCAGTAACCTTGGAGCCAAGTTCGACCCTCCTGAGGGTTTCTCATTGG
ACGAACCTGCTCCAGAGAAGTTTGTAGCAGCAAAGTACTCATCTGGCCAGGATTAA

>SuCAT1
ATGGATCCCTACAAGTTTCGTCCGTCAAGTGCACACAATTCGCCATTCTGGACTACTAA
TTCTGGTGCTCCTGTTTATAACAACAATTCCTCGTTGACCGTTGGTAACAGAGGTCCCA
TTCTTTTAGAGGATTACCATCTAGTGGAGAAACTTGCCAACTTTGACAGGGAGCGTATC
CCAGAACGTGTTGTTCATGCCAGGGGAGCGAGTGCTAAGGGATTTTTTGAAGTCACCC
ATGATGTTTCTCACCTTACATGTGCTGATTTCCTACGCGCCCCTGGTGTCCAGACGCCT
GTTATTGTTCGCTTCTCCACTGTTATTCATGAACGTGGGAGTCCTGAAACACTCAGGGA
CCCTCGTGGTTTTGCTGTGAAGTTCTACACCAGAGAGGGTAACTTTGATATGGTAGGGA
ACAATTTCCCTGTGTTTTTCATCCGTGATGGCATGAAGTTCCCTGATATGGTGCATGCAT
TGAAACCCAACCCCAAATCTCACATCCAAGAAAACTGGAGGATCCTTGATTTCTTCTCT
CACCACCCTGAGAGTTTACACATGTTTTCTTTTCTCTTTGATGATATTGGAATTCCTGCC
AACTACAGACACATGGAAGGGTCTGGTGTCAACACATATATGCTGATTAACAAGGCTG
GGAAAGCTCAATATGTCAAGTTTCATTGGAAACCAACCTGTGGAGTGAAATCTTTGTT
GGAAGATGAGGCCATAAAAGTTGGTGGGGCCAACCATAGTCATGCAACTCAGGACCTC
TTTGATTCGATCGCTGCCGGGAATTATCCCGAATGGAAATTGTTCATCCAAACAATTGA
CCCGGTTGATGAAGATAAGTTTGATTTTGACCCACTAGATGTTACCAAGACATGGCCTG
AGGATATTTTGCCATTACAGCCAGTTGGACGCCTAGTTTTGAACAAGAACATTGACAAC
TTCTTTGCAGAAAATGAGCAGCTGGCATTTTGCCCTGCAATTATTGTTCCTGGTATCTAC
TACTCAGATGACAAGCTACTCCAGACTCGAATTTTCTCATATGCTGATACCCAAAGACA
CCGTCTTGGACCCAACTACCTACAAATTCCTGTTAATGCACCAAAATCTGCTCATCATA
ACAATCACTATGATGGATTCATGAACTTTATGCACAGGGATGAGGAGGTTGATTACTTC
CCTTCTAGATATGATCCTGTTCGTCATGCTGAGAAATATCCCATTCCACCAAATGTCTTG
AGTGGTAGACGTGACAGGCAAATCATTGTAAAGGAGAACAACTTCAAGCAGCCTGGA
GAGAGATACCGGTCCTTTGACCCGGCCAGGCAAGAGAGATTTCTCCAAAGATGGGTTG
AGGCGCTATCTGATCCTCGTATCACTCACGAGATTCGCAGCATCTGGGTTTCATACTGGT
CACAGGCTGACAAGTCATTGGGCATGAAGCTAGCAAGTAGGCTTAATGTGAGGCCAAC
CATGTGA
>SuCAT2
ATGGATACATGCAAGTATCGACCATCAAGTGCTTACAACTCCCCTTACTTGACAACCAA
CACTGGCTCACCGGTTTATAACGACAACTCTGCTTTGACCGTTGGTGCCCGAGGACCA
ATTCTATTGGAAGATTACCATCTGATAGAAAAGATAACAACATGGACTCACGAGCGTAT
CCCGGAGCGTGTGGTACACGCTAGGGGTGCCAGTGCAAAGGGCTTCTTTGAAGTCACT
CATGACATAACTCATCTTACCTGTGCTGATTTCCTTCGTGCTCCCGGTGTTCAAACTCCT
GTCATTGTTCGTTTCTCGACTGTCATCCATGAGCGCGGTAGCCCTGAAACCATCCGTGA
CCCTCGTGGTTTTGCCACCAAGTTCTACACCCGTGAGGGTAACTTTGATTTGGTGGGG
AACAATTTCCCGGTATTTTTCACCCGTGATGCAATGCAGTTCTTTGATCTGATCCGGGCC
TTCAAACCAAACCCGAAATCCCACATCCAAGAGGACTGGAGGTTCTTGGACCACTGCT
CCCACCTCCCTGAGAGCCTCAACACCTTCACCTTCTTCTATGATGACATGGGTATCCCG
ATCAACTACCGACACATGATTGGGTCAGGGGTCCACACCTTCACTATGTTGAACAAGG
CAGGCAAGGTCACCTATGTCAAGTTCCACTGGAGGCCCACGTGTGGCGTCAAGAACAT
GCTTGAGGAGGAAGCTGCTAAAGTCGTTGCAGAAAACCACAGTCACGCGACAAAAGA
TCTTTATGATGCAATTGCCTCAGGAAACTACCCTGAGTGGAAGCTCTTCATCCAAACCA
TGGATCCAGCTGATGAGGATAAGTTTGATTTCGACCCGTTGGATACCACCAAGACTTGG
CCTGAGGATATTTTACCGTTGCAGCCCGTTGGACGGATGGTGTTGAACAAAAACATTG

ATAACTTTTTCAATGAGAACGAGATGCTTGCGTTTAACCCGGCTGCAATTGTTCCTGGG
ATCCATTTCTCTGATGATAAGATGCTTCAGGCTCGAACTTTTGCCTATGCTGATACTCAG
AGATATCGGCTTGGTCCTAATTTCATGCAGCTTCCTGTTAATGCTCCCAAGTGCCCTCAT
CATAACAATCATAGAGACGGGCCTATGAACATGGTTTTCAGGGACGAAGAGGTGGATT
ACTTCCCATCAAGATTTGATCCTGTTCGTGAGGCCGAGAGATTCCCCATCCCTCACTTT
GTAGTTCATGGAAGGCGCGAAAGGCAAATCATTCCAAAGGAGAACAACTTCAAGGAG
CCCGGTGACAGATACAGATCCTGGGACCCAGCCAGGCAAGAGAGGTTTATTGGAAGAT
TCGTCAAGGCTTTGTCCGATCCCCGCCTAACTTATGAGATTCGCAGCATTTGGGTTAATT
GGTTCACTCAGGCAGATAAATCTCTAGGCATGAAAGTGGCAAGCAGACTCAATGTGAG
ACCAACCATGTAA

Amino acid sequences of AsCAT1 and GpCAT1

>AsCAT1
MDPYKFRPSSAHNSPYWTTNSGAPVYNNNSSLTVGNRGPILLEDYHLVEKLANFDRERIP
ERVVHARGASAKGFFEVTHDISHLTCADFLRAPGVQTPVIVRFSTVIHERGSPETLRDPRGF
AVKFYTREGNFDLVGNNFPVFFIRDGMKFPDMVHALKPNPKSHIQENWRIMDFFSHHPES
LHMFTFLFDDIGVPANYRHMEGSGVNTYMLINKAGKAYYVKFHWKPTCGVKSLLEDEAI
KVGGANHSHATQDLYDSIAAGNYPEWKLFIQTIDPADEDKFDFDPLDVTKTWPEDILPLQP
VGRLVLNKNIDNFFAENEQLAFCPAIVVPGVYYSDDKLLQTRIFSYADTQRHRLGPNYLQL
PVNAPKCAHHNNHYDGFMNFMHRDEEVDYFPSRYDPVRHAEKYPIPSNVLNGKRDRQII
VKENNFKQPGERYRSFDPARQERFIQRVVDALSDPRITHEIRSIWVSYWSQADKSLGMKL
ASRLNVRPTM*

>GpCAT1
MDPYKFRPSSAYNSTYFTTNSGAPVYNNNSSLTVGTRGPILLEDYHLVEKLANFDRERIPE
RVVHARGASAKGFFEVTHDISHLSCADFLRAPGVQTPVIVRFSTVIHERGSPETLRDPRGF
AVKFYTREGNFDLVGNNFPVFFVRDGMKFPDMVHALKPNPKNHIQENWRIMDFFSHHPE
SLHMFTFLFDDIGVPADYRHMEGSGVNTYMLVNKAGKAYYVKFHWKPTCGVKSLLEDE
AIKVGGANHSHATQDLHDSIMAGNYPEWKLFIQTIDPADEDKFDFDPLDVTKTWPEDILPL
QPVGRLVLNKNIDNFFAENEQLAFCPGIVVPGVYYSDDKLLQTRIFSYSDTQRHRLGPNYL
QLPVNAPKCAHHNNHHDGFMNFMHRDEEVNYFPSRYDPVRHAEKYPIPSNVLNGKRDK
QVIVKENNFKQPGERYRSFDPARQERFIQRVIDALSDPRVTHEVRAIWVSYWTQADKSLG
MKLASRLNVRPTM*
