# Supplementary figures and images for: Molecular Mechanism of Response and Adaptation of Antioxidant Enzyme System to Salt Stress in Leaves of Gymnocarpos przewalskii
Source: Plants (Basel). 2023 Sep 25;12(19):3370. doi: 10.3390/plants12193370 (PMC10574792; doi:10.3390/plants12193370)

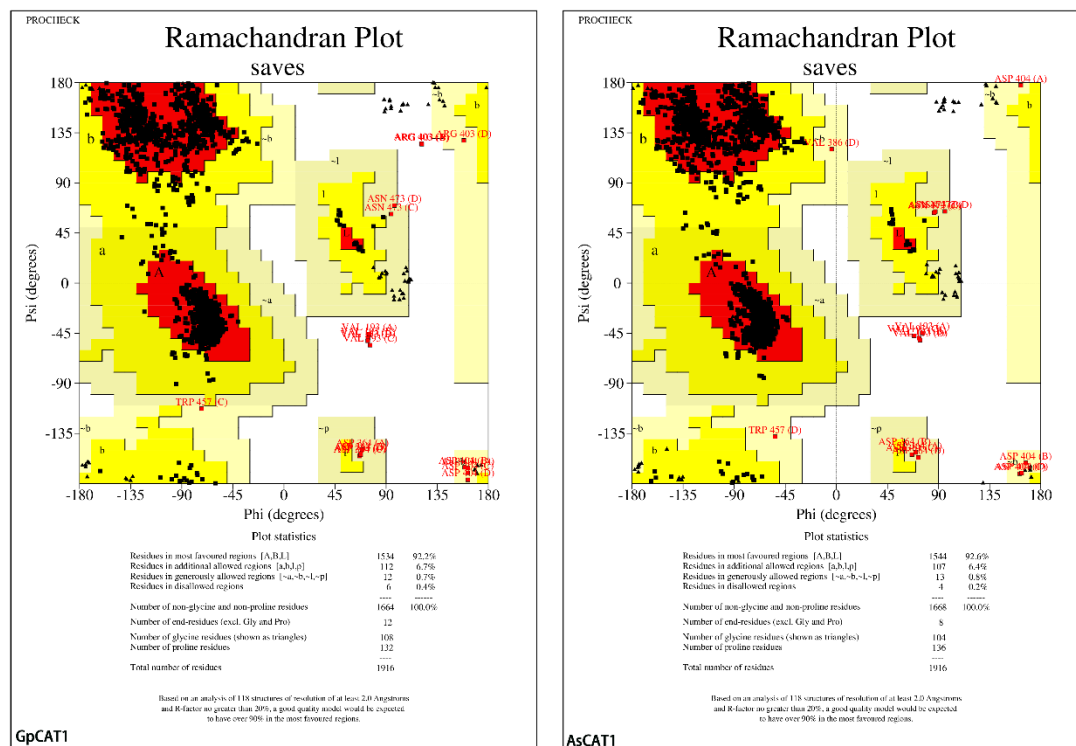

**Figure S1.** GpCAT1 and AsCAT1 protein 3D model quality evaluation results.

Supplement: Supplementary file 1 [file plants-12-03370-s001.zip › Figuer S1 Quality evaluation of protein model.pdf]
